# Supplementary material for: Use of Motivational Interviewing in Older Patients with Multiple Chronic Conditions and Their Informal Caregivers: A Scoping Review
Source: Healthcare (Basel). 2023 Jun 7;11(12):1681. doi: 10.3390/healthcare11121681 (PMC10297925; doi:10.3390/healthcare11121681)
Supplement: Supplementary file 1 [file healthcare-11-01681-s001.zip › Supplementary File 2.pdf]

Supplementary File 2. Database search

PubMed

#1	("Motivational Interviewing"[Mesh] OR ("Motivation"[Mesh] AND "Patient Education as Topic"[Mesh]) OR “motivational interview*”[tiab] OR “motivational technique*”[tiab] OR “motivational counsel*”[tiab] OR “motivational enhancement therap*”[tiab] OR “motivational therap*”[tiab] OR “motivational intervention*”[tiab] OR “motivational strateg*”[tiab] OR “motivational approach*”[tiab] OR “motivational communicat*”[tiab] OR “motivational language*”[tiab])
#2	("Polypharmacy"[Mesh] OR polypharma*[tiab] OR polymedicat*[tiab] OR poly-pharma*[tiab] OR poly-medicat*[tiab] OR multimedicat*[tiab] OR multi-medicat*[tiab] OR plurimedicat*[tiab] OR polypatholog*[tiab] OR poly-patholog*[tiab] OR pluripatholog*[tiab] OR pluri-patholog*[tiab] OR multipatholog*[tiab] OR multi-patholog*[tiab] OR "Comorbidity"[Mesh] OR comorbid*[tiab] OR multimorbid*[tiab] OR multi-morbid*[tiab] OR plurimorbid*[tiab] OR polymorbid*[tiab] OR poly-morbid*[tiab] OR concurrent*[tiab] OR concomitant*[tiab] OR coexist*[tiab] OR co-exist*[tiab] OR "Multiple Chronic Conditions"[Mesh] OR (“Chronic Disease”[Mesh:noexp] OR diseases[tiab] OR conditions[tiab] OR disorders[tiab] OR illnesses[tiab] OR “health problems”[tiab] AND multiple[tiab]))
#3	**("Heart Failure"[Mesh] OR “heart failure”[tiab] OR “heart failures”[tiab])** AND ("Pulmonary Disease, Chronic Obstructive"[Mesh] OR “chronic obstructive pulmonary disease”[tiab] OR “chronic obstructive lung disease”[tiab] OR “chronic obstructive pulmonary diseases”[tiab] OR “chronic obstructive lung diseases”[tiab] OR COPD[tiab] OR "Asthma"[Mesh] OR asthma[tiab] OR asthmatic[tiab] OR "Diabetes Mellitus"[Mesh] OR diabetes[tiab] OR diabetic[tiab] OR "Hypertension"[Mesh] OR hypertension[tiab] OR "Coronary Artery Disease"[Mesh] OR “coronary artery disease”[tiab] OR “coronary atherosclerosis”[tiab] OR “coronary arteriosclerosis”[tiab] OR “coronary artery diseases”[tiab] OR "Arthritis"[Mesh] OR arthritis[tiab] OR arthritic[tiab] OR osteoarthritis[tiab] OR osteoarthritic[tiab] OR periarthritis[tiab] OR periarthritic[tiab] OR "Osteoporosis"[Mesh] OR osteoporosis[tiab] OR osteoporotic[tiab] OR "Neoplasms"[Mesh] OR neoplas*[tiab] OR cancer*[tiab] OR tumor*[tiab] OR tumour*[tiab] OR carcinoma*[tiab] OR oncolog*[tiab] OR "Renal Insufficiency, Chronic"[Mesh] OR “chronic renal insufficiency”[tiab] OR “chronic kidney insufficiency”[tiab] OR “chronic renal disease”[tiab] OR “chronic kidney disease”[tiab] OR “chronic renal insufficiencies”[tiab] OR “chronic renal diseases”[tiab] OR “chronic kidney failure”[tiab] OR “chronic renal failure”[tiab] OR "Renal Dialysis"[Mesh] OR “renal dialysis”[tiab] OR “kidney dialysis”[tiab] OR hemodialysis[tiab] OR haemodialysis[tiab] OR "Hyperlipidemias"[Mesh] OR hyperlipidemia[tiab] OR hyperlipidemias[tiab] OR hyperlipidemic[tiab] OR hyperlipemia[tiab] OR hyperlipemias[tiab] OR hyperlipemic[tiab] OR lipidemia[tiab] OR lipidemias[tiab] OR lipidemic[tiab] OR lipemia[tiab] OR lipemias[tiab] OR lipemic[tiab] OR "Cholesterol"[Mesh] OR hypercholesterolemia[tiab] OR hyper-cholesterolemia[tiab] OR hypercholesterolemic[tiab] OR hyper-cholesterolemic[tiab] OR cholesterol[tiab] OR hypertriglyceridemia[tiab] OR hyper-triglyceridemia[tiab] OR hypertriglyceridemic[tiab] OR hyper-triglyceridemic[tiab] OR "Triglycerides"[Mesh] OR triglycerid[tiab] OR triglycerids[tiab])
#4	(**"Pulmonary Disease, Chronic Obstructive"[Mesh] OR “chronic obstructive pulmonary disease”[tiab] OR “chronic obstructive lung disease”[tiab] OR “chronic obstructive pulmonary diseases”[tiab] OR “chronic obstructive lung diseases”[tiab] OR COPD[tiab]**) AND ("Heart Failure"[Mesh] OR “heart failure”[tiab] OR “heart failures”[tiab] OR "Asthma"[Mesh] OR asthma[tiab] OR asthmatic[tiab] OR "Diabetes Mellitus"[Mesh] OR diabetes[tiab] OR diabetic[tiab] OR "Hypertension"[Mesh] OR

	hypertension[tiab] OR "Coronary Artery Disease"[Mesh] OR “coronary artery disease”[tiab] OR “coronary atherosclerosis”[tiab] OR “coronary arteriosclerosis”[tiab] OR “coronary artery diseases”[tiab] OR "Arthritis"[Mesh] OR arthritis[tiab] OR arthritic[tiab] OR osteoarthritis[tiab] OR osteoarthritic[tiab] OR periarthritis[tiab] OR periarthritic[tiab] OR "Osteoporosis"[Mesh] OR osteoporosis[tiab] OR osteoporotic[tiab] OR "Neoplasms"[Mesh] OR neoplas*[tiab] OR cancer*[tiab] OR tumor*[tiab] OR tumour*[tiab] OR carcinoma*[tiab] OR oncolog*[tiab] OR "Renal Insufficiency, Chronic"[Mesh] OR “chronic renal insufficiency”[tiab] OR “chronic kidney insufficiency”[tiab] OR “chronic renal disease”[tiab] OR “chronic kidney disease”[tiab] OR “chronic renal insufficiencies”[tiab] OR “chronic renal diseases”[tiab] OR “chronic kidney failure”[tiab] OR “chronic renal failure”[tiab] OR "Renal Dialysis"[Mesh] OR “renal dialysis”[tiab] OR “kidney dialysis”[tiab] OR hemodialysis[tiab] OR haemodialysis[tiab] OR "Hyperlipidemias"[Mesh] OR hyperlipidemia[tiab] OR hyperlipidemias[tiab] OR hyperlipidemic[tiab] OR hyperlipemia[tiab] OR hyperlipemias[tiab] OR hyperlipemic[tiab] OR lipidemia[tiab] OR lipidemias[tiab] OR lipidemic[tiab] OR lipemia[tiab] OR lipemias[tiab] OR lipemic[tiab] OR "Cholesterol"[Mesh] OR hypercholesterolemia[tiab] OR hyper-cholesterolemia[tiab] OR hypercholesterolemic[tiab] OR hyper-cholesterolemic[tiab] OR cholesterol[tiab] OR hypertriglyceridemia[tiab] OR hyper-triglyceridemia[tiab] OR hypertriglyceridemic[tiab] OR hyper-triglyceridemic[tiab] OR "Triglycerides"[Mesh] OR triglycerid[tiab] OR triglycerids[tiab])
#5	(**"Asthma"[Mesh] OR asthma[tiab] OR asthmatic[tiab]**) AND ("Heart Failure"[Mesh] OR “heart failure”[tiab] OR “heart failures”[tiab] OR "Pulmonary Disease, Chronic Obstructive"[Mesh] OR “chronic obstructive pulmonary disease”[tiab] OR “chronic obstructive lung disease”[tiab] OR “chronic obstructive pulmonary diseases”[tiab] OR “chronic obstructive lung diseases”[tiab] OR COPD[tiab] OR "Diabetes Mellitus"[Mesh] OR diabetes[tiab] OR diabetic[tiab] OR "Hypertension"[Mesh] OR hypertension[tiab] OR "Coronary Artery Disease"[Mesh] OR “coronary artery disease”[tiab] OR “coronary atherosclerosis”[tiab] OR “coronary arteriosclerosis”[tiab] OR “coronary artery diseases”[tiab] OR "Arthritis"[Mesh] OR arthritis[tiab] OR arthritic[tiab] OR osteoarthritis[tiab] OR osteoarthritic[tiab] OR periarthritis[tiab] OR periarthritic[tiab] OR "Osteoporosis"[Mesh] OR osteoporosis[tiab] OR osteoporotic[tiab] OR "Neoplasms"[Mesh] OR neoplas*[tiab] OR cancer*[tiab] OR tumor*[tiab] OR tumour*[tiab] OR carcinoma*[tiab] OR oncolog*[tiab] OR "Renal Insufficiency, Chronic"[Mesh] OR “chronic renal insufficiency”[tiab] OR “chronic kidney insufficiency”[tiab] OR “chronic renal disease”[tiab] OR “chronic kidney disease”[tiab] OR “chronic renal insufficiencies”[tiab] OR “chronic renal diseases”[tiab] OR “chronic kidney failure”[tiab] OR “chronic renal failure”[tiab] OR "Renal Dialysis"[Mesh] OR “renal dialysis”[tiab] OR “kidney dialysis”[tiab] OR hemodialysis[tiab] OR haemodialysis[tiab] OR "Hyperlipidemias"[Mesh] OR hyperlipidemia[tiab] OR hyperlipidemias[tiab] OR hyperlipidemic[tiab] OR hyperlipemia[tiab] OR hyperlipemias[tiab] OR hyperlipemic[tiab] OR lipidemia[tiab] OR lipidemias[tiab] OR lipidemic[tiab] OR lipemia[tiab] OR lipemias[tiab] OR lipemic[tiab] OR "Cholesterol"[Mesh] OR hypercholesterolemia[tiab] OR hyper-cholesterolemia[tiab] OR hypercholesterolemic[tiab] OR hyper-cholesterolemic[tiab] OR cholesterol[tiab] OR hypertriglyceridemia[tiab] OR hyper-triglyceridemia[tiab] OR hypertriglyceridemic[tiab] OR hyper-triglyceridemic[tiab] OR "Triglycerides"[Mesh] OR triglycerid[tiab] OR triglycerids[tiab])
#6	(**"Diabetes Mellitus"[Mesh] OR diabetes[tiab] OR diabetic[tiab]**) AND ("Heart Failure"[Mesh] OR “heart failure”[tiab] OR “heart failures”[tiab] OR "Pulmonary Disease, Chronic Obstructive"[Mesh] OR “chronic obstructive pulmonary disease”[tiab] OR “chronic obstructive lung disease”[tiab] OR “chronic obstructive pulmonary diseases”[tiab] OR “chronic obstructive lung diseases”[tiab] OR COPD[tiab] OR "Asthma"[Mesh] OR

	asthma[tiab] OR asthmatic[tiab] OR "Hypertension"[Mesh] OR hypertension[tiab] OR "Coronary Artery Disease"[Mesh] OR “coronary artery disease”[tiab] OR “coronary atherosclerosis”[tiab] OR “coronary arteriosclerosis”[tiab] OR “coronary artery diseases”[tiab] OR "Arthritis"[Mesh] OR arthritis[tiab] OR arthritic[tiab] OR osteoarthritis[tiab] OR osteoarthritic[tiab] OR periarthritis[tiab] OR periarthritic[tiab] OR "Osteoporosis"[Mesh] OR osteoporosis[tiab] OR osteoporotic[tiab] OR "Neoplasms"[Mesh] OR neoplas*[tiab] OR cancer*[tiab] OR tumor*[tiab] OR tumour*[tiab] OR carcinoma*[tiab] OR oncolog*[tiab] OR "Renal Insufficiency, Chronic"[Mesh] OR “chronic renal insufficiency”[tiab] OR “chronic kidney insufficiency”[tiab] OR “chronic renal disease”[tiab] OR “chronic kidney disease”[tiab] OR “chronic renal insufficiencies”[tiab] OR “chronic renal diseases”[tiab] OR “chronic kidney failure”[tiab] OR “chronic renal failure”[tiab] OR "Renal Dialysis"[Mesh] OR “renal dialysis”[tiab] OR “kidney dialysis”[tiab] OR hemodialysis[tiab] OR haemodialysis[tiab] OR "Hyperlipidemias"[Mesh] OR hyperlipidemia[tiab] OR hyperlipidemias[tiab] OR hyperlipidemic[tiab] OR hyperlipemia[tiab] OR hyperlipemias[tiab] OR hyperlipemic[tiab] OR lipidemia[tiab] OR lipidemias[tiab] OR lipidemic[tiab] OR lipemia[tiab] OR lipemias[tiab] OR lipemic[tiab] OR "Cholesterol"[Mesh] OR hypercholesterolemia[tiab] OR hyper-cholesterolemia[tiab] OR hypercholesterolemic[tiab] OR hyper-cholesterolemic[tiab] OR cholesterol[tiab] OR hypertriglyceridemia[tiab] OR hyper-triglyceridemia[tiab] OR hypertriglyceridemic[tiab] OR hyper-triglyceridemic[tiab] OR "Triglycerides"[Mesh] OR triglycerid[tiab] OR triglycerids[tiab])
#7	(**"Hypertension"[Mesh] OR hypertension[tiab]**) AND ("Heart Failure"[Mesh] OR “heart failure”[tiab] OR “heart failures”[tiab] OR “heart failures”[tiab] OR "Pulmonary Disease, Chronic Obstructive"[Mesh] OR “chronic obstructive pulmonary disease”[tiab] OR “chronic obstructive lung disease”[tiab] OR “chronic obstructive pulmonary diseases”[tiab] OR “chronic obstructive lung diseases”[tiab] OR COPD[tiab] OR "Asthma"[Mesh] OR asthma[tiab] OR asthmatic[tiab] OR "Diabetes Mellitus"[Mesh] OR diabetes[tiab] OR diabetic[tiab] OR "Coronary Artery Disease"[Mesh] OR “coronary artery disease”[tiab] OR “coronary atherosclerosis”[tiab] OR “coronary arteriosclerosis”[tiab] OR “coronary artery diseases”[tiab] OR "Arthritis"[Mesh] OR arthritis[tiab] OR arthritic[tiab] OR osteoarthritis[tiab] OR osteoarthritic[tiab] OR periarthritis[tiab] OR periarthritic[tiab] OR "Osteoporosis"[Mesh] OR osteoporosis[tiab] OR osteoporotic[tiab] OR "Neoplasms"[Mesh] OR neoplas*[tiab] OR cancer*[tiab] OR tumor*[tiab] OR tumour*[tiab] OR carcinoma*[tiab] OR oncolog*[tiab] OR "Renal Insufficiency, Chronic"[Mesh] OR “chronic renal insufficiency”[tiab] OR “chronic kidney insufficiency”[tiab] OR “chronic renal disease”[tiab] OR “chronic kidney disease”[tiab] OR “chronic renal insufficiencies”[tiab] OR “chronic renal diseases”[tiab] OR “chronic kidney failure”[tiab] OR “chronic renal failure”[tiab] OR "Renal Dialysis"[Mesh] OR “renal dialysis”[tiab] OR “kidney dialysis”[tiab] OR hemodialysis[tiab] OR haemodialysis[tiab] OR "Hyperlipidemias"[Mesh] OR hyperlipidemia[tiab] OR hyperlipidemias[tiab] OR hyperlipidemic[tiab] OR hyperlipemia[tiab] OR hyperlipemias[tiab] OR hyperlipemic[tiab] OR lipidemia[tiab] OR lipidemias[tiab] OR lipidemic[tiab] OR lipemia[tiab] OR lipemias[tiab] OR lipemic[tiab] OR "Cholesterol"[Mesh] OR hypercholesterolemia[tiab] OR hyper-cholesterolemia[tiab] OR hypercholesterolemic[tiab] OR hyper-cholesterolemic[tiab] OR cholesterol[tiab] OR hypertriglyceridemia[tiab] OR hyper-triglyceridemia[tiab] OR hypertriglyceridemic[tiab] OR hyper-triglyceridemic[tiab] OR "Triglycerides"[Mesh] OR triglycerid[tiab] OR triglycerids[tiab])
#8	(**"Coronary Artery Disease"[Mesh] OR “coronary artery disease”[tiab] OR “coronary atherosclerosis”[tiab] OR “coronary arteriosclerosis”[tiab] OR “coronary artery diseases”[tiab]**) AND ("Heart Failure"[Mesh] OR “heart failure”[tiab] OR “heart failures”[tiab] OR "Pulmonary Disease, Chronic Obstructive"[Mesh] OR “chronic

	obstructive pulmonary disease"[tiab] OR "chronic obstructive lung disease"[tiab] OR "chronic obstructive pulmonary diseases"[tiab] OR "chronic obstructive lung diseases"[tiab] OR COPD[tiab] OR "Asthma"[Mesh] OR asthma[tiab] OR asthmatic[tiab] OR "Diabetes Mellitus"[Mesh] OR diabetes[tiab] OR diabetic[tiab] OR "Hypertension"[Mesh] OR hypertension[tiab] OR "Arthritis"[Mesh] OR arthritis[tiab] OR arthritic[tiab] OR osteoarthritis[tiab] OR osteoarthritic[tiab] OR periarthritis[tiab] OR periarthritic[tiab] OR "Osteoporosis"[Mesh] OR osteoporosis[tiab] OR osteoporotic[tiab] OR "Neoplasms"[Mesh] OR neoplas*[tiab] OR cancer*[tiab] OR tumor*[tiab] OR tumour*[tiab] OR carcinoma*[tiab] OR oncolog*[tiab] OR "Renal Insufficiency, Chronic"[Mesh] OR "chronic renal insufficiency"[tiab] OR "chronic kidney insufficiency"[tiab] OR "chronic renal disease"[tiab] OR "chronic kidney disease"[tiab] OR "chronic renal insufficiencies"[tiab] OR "chronic renal diseases"[tiab] OR "chronic kidney failure"[tiab] OR "chronic renal failure"[tiab] OR "Renal Dialysis"[Mesh] OR "renal dialysis"[tiab] OR "kidney dialysis"[tiab] OR hemodialysis[tiab] OR haemodialysis[tiab] OR "Hyperlipidemias"[Mesh] OR hyperlipidemia[tiab] OR hyperlipidemias[tiab] OR hyperlipidemic[tiab] OR hyperlipemia[tiab] OR hyperlipemias[tiab] OR hyperlipemic[tiab] OR lipidemia[tiab] OR lipidemias[tiab] OR lipidemic[tiab] OR lipemia[tiab] OR lipemias[tiab] OR lipemic[tiab] OR "Cholesterol"[Mesh] OR hypercholesterolemia[tiab] OR hyper-cholesterolemia[tiab] OR hypercholesterolemic[tiab] OR hyper-cholesterolemic[tiab] OR cholesterol[tiab] OR hypertriglyceridemia[tiab] OR hyper-triglyceridemia[tiab] OR hypertriglyceridemic[tiab] OR hyper-triglyceridemic[tiab] OR "Triglycerides"[Mesh] OR triglycerid[tiab] OR triglycerids[tiab])
#9	(**"Arthritis"[Mesh] OR arthritis[tiab] OR arthritic[tiab] OR osteoarthritis[tiab] OR osteoarthritic[tiab] OR periarthritis[tiab] OR periarthritic[tiab]**) AND ("Heart Failure"[Mesh] OR "heart failure"[tiab] OR "heart failures"[tiab] OR "Pulmonary Disease, Chronic Obstructive"[Mesh] OR "chronic obstructive pulmonary disease"[tiab] OR "chronic obstructive lung disease"[tiab] OR "chronic obstructive pulmonary diseases"[tiab] OR "chronic obstructive lung diseases"[tiab] OR COPD[tiab] OR "Asthma"[Mesh] OR asthma[tiab] OR asthmatic[tiab] OR "Diabetes Mellitus"[Mesh] OR diabetes[tiab] OR diabetic[tiab] OR "Hypertension"[Mesh] OR hypertension[tiab] OR "Coronary Artery Disease"[Mesh] OR "coronary artery disease"[tiab] OR "coronary atherosclerosis"[tiab] OR "coronary arteriosclerosis"[tiab] OR "coronary artery diseases"[tiab] OR "Osteoporosis"[Mesh] OR osteoporosis[tiab] OR osteoporotic[tiab] OR "Neoplasms"[Mesh] OR neoplas*[tiab] OR cancer*[tiab] OR tumor*[tiab] OR tumour*[tiab] OR carcinoma*[tiab] OR oncolog*[tiab] OR "Renal Insufficiency, Chronic"[Mesh] OR "chronic renal insufficiency"[tiab] OR "chronic kidney insufficiency"[tiab] OR "chronic renal disease"[tiab] OR "chronic kidney disease"[tiab] OR "chronic renal insufficiencies"[tiab] OR "chronic renal diseases"[tiab] OR "chronic kidney failure"[tiab] OR "chronic renal failure"[tiab] OR "Renal Dialysis"[Mesh] OR "renal dialysis"[tiab] OR "kidney dialysis"[tiab] OR hemodialysis[tiab] OR haemodialysis[tiab] OR "Hyperlipidemias"[Mesh] OR hyperlipidemia[tiab] OR hyperlipidemias[tiab] OR hyperlipidemic[tiab] OR hyperlipemia[tiab] OR hyperlipemias[tiab] OR hyperlipemic[tiab] OR lipidemia[tiab] OR lipidemias[tiab] OR lipidemic[tiab] OR lipemia[tiab] OR lipemias[tiab] OR lipemic[tiab] OR "Cholesterol"[Mesh] OR hypercholesterolemia[tiab] OR hyper-cholesterolemia[tiab] OR hypercholesterolemic[tiab] OR hyper-cholesterolemic[tiab] OR cholesterol[tiab] OR hypertriglyceridemia[tiab] OR hyper-triglyceridemia[tiab] OR hypertriglyceridemic[tiab] OR hyper-triglyceridemic[tiab] OR "Triglycerides"[Mesh] OR triglycerid[tiab] OR triglycerids[tiab])
#10	(**"Osteoporosis"[Mesh] OR osteoporosis[tiab] OR osteoporotic[tiab]**) AND ("Heart Failure"[Mesh] OR "heart failure"[tiab] OR "heart failures"[tiab] OR "Pulmonary Disease, Chronic Obstructive"[Mesh] OR "chronic obstructive pulmonary disease"[tiab] OR "chronic

	obstructive lung disease”[tiab] OR “chronic obstructive pulmonary diseases”[tiab] OR “chronic obstructive lung diseases”[tiab] OR COPD[tiab] OR "Asthma"[Mesh] OR asthma[tiab] OR asthmatic[tiab] OR "Diabetes Mellitus"[Mesh] OR diabetes[tiab] OR diabetic[tiab] OR "Hypertension"[Mesh] OR hypertension[tiab] OR "Coronary Artery Disease"[Mesh] OR “coronary artery disease”[tiab] OR “coronary atherosclerosis”[tiab] OR “coronary arteriosclerosis”[tiab] OR “coronary artery diseases”[tiab] OR "Arthritis"[Mesh] OR arthritis[tiab] OR arthritic[tiab] OR osteoarthritis[tiab] OR osteoarthritic[tiab] OR periarthritis[tiab] OR periarthritic[tiab] OR "Neoplasms"[Mesh] OR neoplas*[tiab] OR cancer*[tiab] OR tumor*[tiab] OR tumour*[tiab] OR carcinoma*[tiab] OR oncolog*[tiab] OR "Renal Insufficiency, Chronic"[Mesh] OR “chronic renal insufficiency”[tiab] OR “chronic kidney insufficiency”[tiab] OR “chronic renal disease”[tiab] OR “chronic kidney disease”[tiab] OR “chronic renal insufficiencies”[tiab] OR “chronic renal diseases”[tiab] OR “chronic kidney failure”[tiab] OR “chronic renal failure”[tiab] OR "Renal Dialysis"[Mesh] OR “renal dialysis”[tiab] OR “kidney dialysis”[tiab] OR hemodialysis[tiab] OR haemodialysis[tiab] OR "Hyperlipidemias"[Mesh] OR hyperlipidemia[tiab] OR hyperlipidemias[tiab] OR hyperlipidemic[tiab] OR hyperlipemia[tiab] OR hyperlipemias[tiab] OR hyperlipemic[tiab] OR lipidemia[tiab] OR lipidemias[tiab] OR lipidemic[tiab] OR lipemia[tiab] OR lipemias[tiab] OR lipemic[tiab] OR "Cholesterol"[Mesh] OR hypercholesterolemia[tiab] OR hyper-cholesterolemia[tiab] OR hypercholesterolemic[tiab] OR hyper-cholesterolemic[tiab] OR cholesterol[tiab] OR hypertriglyceridemia[tiab] OR hyper-triglyceridemia[tiab] OR hypertriglyceridemic[tiab] OR hyper-triglyceridemic[tiab] OR "Triglycerides"[Mesh] OR triglycerid[tiab] OR triglycerids[tiab])
#11	(**"Neoplasms"[Mesh] OR neoplas*[tiab] OR cancer*[tiab] OR tumor*[tiab] OR tumour*[tiab] OR carcinoma*[tiab] OR oncolog*[tiab]**) AND ("Heart Failure"[Mesh] OR “heart failure”[tiab] OR “heart failures”[tiab] OR "Pulmonary Disease, Chronic Obstructive"[Mesh] OR “chronic obstructive pulmonary disease”[tiab] OR “chronic obstructive lung disease”[tiab] OR “chronic obstructive pulmonary diseases”[tiab] OR “chronic obstructive lung diseases”[tiab] OR COPD[tiab] OR "Asthma"[Mesh] OR asthma[tiab] OR asthmatic[tiab] OR "Diabetes Mellitus"[Mesh] OR diabetes[tiab] OR diabetic[tiab] OR "Hypertension"[Mesh] OR hypertension[tiab] OR "Coronary Artery Disease"[Mesh] OR “coronary artery disease”[tiab] OR “coronary atherosclerosis”[tiab] OR “coronary arteriosclerosis”[tiab] OR “coronary artery diseases”[tiab] OR "Arthritis"[Mesh] OR arthritis[tiab] OR arthritic[tiab] OR osteoarthritis[tiab] OR osteoarthritic[tiab] OR periarthritis[tiab] OR periarthritic[tiab] OR "Osteoporosis"[Mesh] OR osteoporosis[tiab] OR osteoporotic[tiab] OR "Renal Insufficiency, Chronic"[Mesh] OR “chronic renal insufficiency”[tiab] OR “chronic kidney insufficiency”[tiab] OR “chronic renal disease”[tiab] OR “chronic kidney disease”[tiab] OR “chronic renal insufficiencies”[tiab] OR “chronic renal diseases”[tiab] OR “chronic kidney failure”[tiab] OR “chronic renal failure”[tiab] OR "Renal Dialysis"[Mesh] OR “renal dialysis”[tiab] OR “kidney dialysis”[tiab] OR hemodialysis[tiab] OR haemodialysis[tiab] OR "Hyperlipidemias"[Mesh] OR hyperlipidemia[tiab] OR hyperlipidemias[tiab] OR hyperlipidemic[tiab] OR hyperlipemia[tiab] OR hyperlipemias[tiab] OR hyperlipemic[tiab] OR lipidemia[tiab] OR lipidemias[tiab] OR lipidemic[tiab] OR lipemia[tiab] OR lipemias[tiab] OR lipemic[tiab] OR "Cholesterol"[Mesh] OR hypercholesterolemia[tiab] OR hyper-cholesterolemia[tiab] OR hypercholesterolemic[tiab] OR hyper-cholesterolemic[tiab] OR cholesterol[tiab] OR hypertriglyceridemia[tiab] OR hyper-triglyceridemia[tiab] OR hypertriglyceridemic[tiab] OR hyper-triglyceridemic[tiab] OR "Triglycerides"[Mesh] OR triglycerid[tiab] OR triglycerids[tiab])
#12	(**"Renal Insufficiency, Chronic"[Mesh] OR “chronic renal insufficiency”[tiab] OR “chronic kidney insufficiency”[tiab] OR “chronic renal disease”[tiab] OR “chronic**

	**kidney disease"[tiab] OR "chronic renal insufficiencies"[tiab] OR "chronic renal diseases"[tiab] OR "chronic kidney diseases"[tiab] OR "chronic kidney failure"[tiab] OR "chronic renal failure"[tiab] OR "Renal Dialysis"[Mesh] OR "renal dialysis"[tiab] OR "kidney dialysis"[tiab] OR hemodialysis[tiab] OR haemodialysis[tiab]**) AND ("Heart Failure"[Mesh] OR "heart failure"[tiab] OR "heart failures"[tiab] OR "Pulmonary Disease, Chronic Obstructive"[Mesh] OR "chronic obstructive pulmonary disease"[tiab] OR "chronic obstructive lung disease"[tiab] OR "chronic obstructive pulmonary diseases"[tiab] OR "chronic obstructive lung diseases"[tiab] OR COPD[tiab] OR "Asthma"[Mesh] OR asthma[tiab] OR asthmatic[tiab] OR "Diabetes Mellitus"[Mesh] OR diabetes[tiab] OR diabetic[tiab] OR "Hypertension"[Mesh] OR hypertension[tiab] OR "Coronary Artery Disease"[Mesh] OR "coronary artery disease"[tiab] OR "coronary atherosclerosis"[tiab] OR "coronary arteriosclerosis"[tiab] OR "coronary artery diseases"[tiab] OR "Arthritis"[Mesh] OR arthritis[tiab] OR arthritic[tiab] OR osteoarthritis[tiab] OR osteoarthritic[tiab] OR periarthritis[tiab] OR periarthritic[tiab] OR "Osteoporosis"[Mesh] OR osteoporosis[tiab] OR osteoporotic[tiab] OR "Neoplasms"[Mesh] OR neoplas*[tiab] OR cancer*[tiab] OR tumor*[tiab] OR tumour*[tiab] OR carcinoma*[tiab] OR oncolog*[tiab] OR "Hyperlipidemias"[Mesh] OR hyperlipidemia[tiab] OR hyperlipidemias[tiab] OR hyperlipidemic[tiab] OR hyperlipemia[tiab] OR hyperlipemias[tiab] OR hyperlipemic[tiab] OR lipidemia[tiab] OR lipidemias[tiab] OR lipidemic[tiab] OR lipemia[tiab] OR lipemias[tiab] OR lipemic[tiab] OR "Cholesterol"[Mesh] OR hypercholesterolemia[tiab] OR hyper-cholesterolemia[tiab] OR hypercholesterolemic[tiab] OR hyper-cholesterolemic[tiab] OR cholesterol[tiab] OR hypertriglyceridemia[tiab] OR hyper-triglyceridemia[tiab] OR hypertriglyceridemic[tiab] OR hyper-triglyceridemic[tiab] OR "Triglycerides"[Mesh] OR triglycerid[tiab] OR triglycerids[tiab])
#13	(**"Hyperlipidemias"[Mesh] OR hyperlipidemia[tiab] OR hyperlipidemias[tiab] OR hyperlipidemic[tiab] OR hyperlipemia[tiab] OR hyperlipemias[tiab] OR hyperlipemic[tiab] OR lipidemia[tiab] OR lipidemias[tiab] OR lipidemic[tiab] OR lipemia[tiab] OR lipemias[tiab] OR lipemic[tiab] OR "Cholesterol"[Mesh] OR hypercholesterolemia[tiab] OR hyper-cholesterolemia[tiab] OR hypercholesterolemic[tiab] OR hyper-cholesterolemic[tiab] OR cholesterol[tiab] OR hypertriglyceridemia[tiab] OR hyper-triglyceridemia[tiab] OR hypertriglyceridemic[tiab] OR hyper-triglyceridemic[tiab] OR "Triglycerides"[Mesh] OR triglycerid[tiab] OR triglycerids[tiab]**) AND ("Heart Failure"[Mesh] OR "heart failure"[tiab] OR "heart failures"[tiab] OR "Pulmonary Disease, Chronic Obstructive"[Mesh] OR "chronic obstructive pulmonary disease"[tiab] OR "chronic obstructive lung disease"[tiab] OR "chronic obstructive pulmonary diseases"[tiab] OR "chronic obstructive lung diseases"[tiab] OR COPD[tiab] OR "Asthma"[Mesh] OR asthma[tiab] OR asthmatic[tiab] OR "Diabetes Mellitus"[Mesh] OR diabetes[tiab] OR diabetic[tiab] OR "Hypertension"[Mesh] OR hypertension[tiab] OR "Coronary Artery Disease"[Mesh] OR "coronary artery disease"[tiab] OR "coronary atherosclerosis"[tiab] OR "coronary arteriosclerosis"[tiab] OR "coronary artery diseases"[tiab] OR "Arthritis"[Mesh] OR arthritis[tiab] OR arthritic[tiab] OR osteoarthritis[tiab] OR osteoarthritic[tiab] OR periarthritis[tiab] OR periarthritic[tiab] OR "Osteoporosis"[Mesh] OR osteoporosis[tiab] OR osteoporotic[tiab] OR "Neoplasms"[Mesh] OR neoplas*[tiab] OR cancer*[tiab] OR tumor*[tiab] OR tumour*[tiab] OR carcinoma*[tiab] OR oncolog*[tiab] OR "Renal Insufficiency, Chronic"[Mesh] OR "chronic renal insufficiency"[tiab] OR "chronic kidney insufficiency"[tiab] OR "chronic renal disease"[tiab] OR "chronic kidney disease"[tiab] OR "chronic renal insufficiencies"[tiab] OR "chronic renal diseases"[tiab] OR "chronic kidney failure"[tiab] OR "chronic renal failure"[tiab] OR "Renal Dialysis"[Mesh] OR "renal dialysis"[tiab] OR "kidney dialysis"[tiab] OR hemodialysis[tiab] OR haemodialysis[tiab])
**#14**	**#2 OR #3 OR #4 OR #5 OR #6 OR #7 OR #8 OR #9 OR #10 OR #11 OR #12 OR #13**

#15	"Aged"[Mesh] OR aging[tiab] OR older[tiab] OR elder*[tiab] OR geriatr*[tiab]
**#16**	**#1 AND #14 AND #15**

EMBASE

#1	('motivational interviewing'/exp OR ('motivation'/exp AND 'patient education'/exp) OR ‘motivational interview*’:ti,ab,kw OR ‘motivational technique*’:ti,ab,kw OR ‘motivational counsel*’:ti,ab,kw OR ‘motivational enhancement therap*’:ti,ab,kw OR ‘motivational therap*’:ti,ab,kw OR ‘motivational intervention*’:ti,ab,kw OR ‘motivational strateg*’:ti,ab,kw OR ‘motivational approach*’:ti,ab,kw OR ‘motivational communicat*’:ti,ab,kw OR ‘motivational language*’:ti,ab,kw)
#2	('polypharmacy'/exp OR polypharma*:ti,ab,kw OR polymedicat*:ti,ab,kw OR poly-pharma*:ti,ab,kw OR poly-medicat*:ti,ab,kw OR multimedicat*:ti,ab,kw OR multi-medicat*:ti,ab,kw OR plurimedicat*:ti,ab,kw OR polypatholog*:ti,ab,kw OR poly-patholog*:ti,ab,kw OR pluripatholog*:ti,ab,kw OR pluri-patholog*:ti,ab,kw OR multipatholog*:ti,ab,kw OR multi-patholog*:ti,ab,kw OR 'comorbidity'/exp OR comorbid*:ti,ab,kw OR multimorbid*:ti,ab,kw OR multi-morbid*:ti,ab,kw OR plurimorbid*:ti,ab,kw OR polymorbid*:ti,ab,kw OR poly-morbid*:ti,ab,kw OR concurrent*:ti,ab,kw OR concomitant*:ti,ab,kw OR coexist*:ti,ab,kw OR co-exist*:ti,ab,kw OR 'multiple chronic conditions'/exp OR ('chronic disease'/de OR diseases:ti,ab,kw OR conditions:ti,ab,kw OR disorders:ti,ab,kw OR illnesses:ti,ab,kw OR ‘health problems’:ti,ab,kw AND multiple:ti,ab,kw))
**#3**	**(‘heart failure’/exp OR ‘heart failure’:ti,ab,kw OR ‘heart failures’:ti,ab,kw)** AND (‘chronic obstructive lung disease’/exp OR ‘chronic obstructive pulmonary disease’:ti,ab,kw OR ‘chronic obstructive lung disease’:ti,ab,kw OR ‘chronic obstructive pulmonary diseases’:ti,ab,kw OR ‘chronic obstructive lung diseases’:ti,ab,kw OR COPD:ti,ab,kw OR ‘asthma’/exp OR asthma:ti,ab,kw OR asthmatic:ti,ab,kw OR ‘diabetes mellitus’/exp OR diabetes:ti,ab,kw OR diabetic:ti,ab,kw OR ‘hypertension’/exp OR hypertension:ti,ab,kw OR ‘coronary artery disease’/exp OR ‘coronary artery disease’:ti,ab,kw OR ‘coronary atherosclerosis’:ti,ab,kw OR ‘coronary arteriosclerosis’:ti,ab,kw OR ‘coronary artery diseases’:ti,ab,kw OR ‘arthritis’/exp OR arthritis:ti,ab,kw OR arthritic:ti,ab,kw OR osteoarthritis:ti,ab,kw OR osteoarthritic:ti,ab,kw OR periarthritis:ti,ab,kw OR periarthritic:ti,ab,kw OR ‘osteoporosis’/exp OR osteoporosis:ti,ab,kw OR osteoporotic:ti,ab,kw OR ‘neoplasm’/exp OR neoplas*:ti,ab,kw OR cancer*:ti,ab,kw OR tumor*:ti,ab,kw OR tumour*:ti,ab,kw OR carcinoma*:ti,ab,kw OR oncolog*:ti,ab,kw OR ‘chronic kidney failure’/exp OR ‘chronic renal insufficiency’:ti,ab,kw OR ‘chronic kidney insufficiency’:ti,ab,kw OR ‘chronic renal disease’:ti,ab,kw OR ‘chronic kidney disease’:ti,ab,kw OR ‘chronic renal insufficiencies’:ti,ab,kw OR ‘chronic renal diseases’:ti,ab,kw OR ‘chronic kidney failure’:ti,ab,kw OR ‘chronic renal failure’:ti,ab,kw OR ‘hemodialysis’/exp OR ‘renal dialysis’:ti,ab,kw OR ‘kidney dialysis’:ti,ab,kw OR hemodialysis:ti,ab,kw OR haemodialysis:ti,ab,kw OR ‘hyperlipidemia’/exp OR hyperlipidemia:ti,ab,kw OR hyperlipidemias:ti,ab,kw OR hyperlipidemic:ti,ab,kw OR hyperlipemia:ti,ab,kw OR hyperlipemias:ti,ab,kw OR hyperlipemic:ti,ab,kw OR lipidemia:ti,ab,kw OR lipidemias:ti,ab,kw OR lipidemic:ti,ab,kw OR lipemia:ti,ab,kw OR lipemias:ti,ab,kw OR lipemic:ti,ab,kw OR ‘cholesterol’/exp OR hypercholesterolemia:ti,ab,kw OR hyper-cholesterolemia:ti,ab,kw OR hypercholesterolemic:ti,ab,kw OR hyper-cholesterolemic:ti,ab,kw OR cholesterol:ti,ab,kw OR hypertriglyceridemia:ti,ab,kw OR hyper-triglyceridemia:ti,ab,kw OR hypertriglyceridemic:ti,ab,kw OR hyper-triglyceridemic:ti,ab,kw OR 'triacylglycerol'/exp OR triglycerid:ti,ab,kw OR triglycerids:ti,ab,kw)
#4	**(‘chronic obstructive lung disease’/exp OR ‘chronic obstructive pulmonary disease’:ti,ab,kw OR ‘chronic obstructive lung disease’:ti,ab,kw OR ‘chronic obstructive pulmonary diseases’:ti,ab,kw OR ‘chronic obstructive lung diseases’:ti,ab,kw OR COPD:ti,ab,kw)** AND (‘heart failure’/exp OR ‘heart

	failure':ti,ab,kw OR 'heart failures':ti,ab,kw OR 'asthma'/exp OR asthma:ti,ab,kw OR asthmatic:ti,ab,kw OR 'diabetes mellitus'/exp OR diabetes:ti,ab,kw OR diabetic:ti,ab,kw OR 'hypertension'/exp OR hypertension:ti,ab,kw OR 'coronary artery disease'/exp OR 'coronary artery disease':ti,ab,kw OR 'coronary atherosclerosis':ti,ab,kw OR 'coronary arteriosclerosis':ti,ab,kw OR 'coronary artery diseases':ti,ab,kw OR 'arthritis'/exp OR arthritis:ti,ab,kw OR arthritic:ti,ab,kw OR osteoarthritis:ti,ab,kw OR osteoarthritic:ti,ab,kw OR periarthritis:ti,ab,kw OR periarthritic:ti,ab,kw OR 'osteoporosis'/exp OR osteoporosis:ti,ab,kw OR osteoporotic:ti,ab,kw OR 'neoplasm'/exp OR neoplas*:ti,ab,kw OR cancer*:ti,ab,kw OR tumor*:ti,ab,kw OR tumour*:ti,ab,kw OR carcinoma*:ti,ab,kw OR oncolog*:ti,ab,kw OR 'chronic kidney failure'/exp OR 'chronic renal insufficiency':ti,ab,kw OR 'chronic kidney insufficiency':ti,ab,kw OR 'chronic renal disease':ti,ab,kw OR 'chronic kidney disease':ti,ab,kw OR 'chronic renal insufficiencies':ti,ab,kw OR 'chronic renal diseases':ti,ab,kw OR 'chronic kidney failure':ti,ab,kw OR 'chronic renal failure':ti,ab,kw OR 'hemodialysis'/exp OR 'renal dialysis':ti,ab,kw OR 'kidney dialysis':ti,ab,kw OR hemodialysis:ti,ab,kw OR haemodialysis:ti,ab,kw OR 'hyperlipidemia'/exp OR hyperlipidemia:ti,ab,kw OR hyperlipidemias:ti,ab,kw OR hyperlipidemic:ti,ab,kw OR hyperlipemia:ti,ab,kw OR hyperlipemias:ti,ab,kw OR hyperlipemic:ti,ab,kw OR lipidemia:ti,ab,kw OR lipidemias:ti,ab,kw OR lipidemic:ti,ab,kw OR lipemia:ti,ab,kw OR lipemias:ti,ab,kw OR lipemic:ti,ab,kw OR 'cholesterol'/exp OR hypercholesterolemia:ti,ab,kw OR hyper-cholesterolemia:ti,ab,kw OR hypercholesterolemic:ti,ab,kw OR hyper-cholesterolemic:ti,ab,kw OR cholesterol:ti,ab,kw OR hypertriglyceridemia:ti,ab,kw OR hyper-triglyceridemia:ti,ab,kw OR hypertriglyceridemic:ti,ab,kw OR hyper-triglyceridemic:ti,ab,kw OR 'triacylglycerol'/exp OR triglycerid:ti,ab,kw OR triglycerids:ti,ab,kw)
#5	(**'asthma'/exp OR asthma:ti,ab,kw OR asthmatic:ti,ab,kw**) AND ('heart failure'/exp OR 'heart failure':ti,ab,kw OR 'heart failures':ti,ab,kw OR 'chronic obstructive lung disease'/exp OR 'chronic obstructive pulmonary disease':ti,ab,kw OR 'chronic obstructive lung disease':ti,ab,kw OR 'chronic obstructive pulmonary diseases':ti,ab,kw OR 'chronic obstructive lung diseases':ti,ab,kw OR COPD:ti,ab,kw OR 'diabetes mellitus'/exp OR diabetes:ti,ab,kw OR diabetic:ti,ab,kw OR 'hypertension'/exp OR hypertension:ti,ab,kw OR 'coronary artery disease'/exp OR 'coronary artery disease':ti,ab,kw OR 'coronary atherosclerosis':ti,ab,kw OR 'coronary arteriosclerosis':ti,ab,kw OR 'coronary artery diseases':ti,ab,kw OR 'arthritis'/exp OR arthritis:ti,ab,kw OR arthritic:ti,ab,kw OR osteoarthritis:ti,ab,kw OR osteoarthritic:ti,ab,kw OR periarthritis:ti,ab,kw OR periarthritic:ti,ab,kw OR 'osteoporosis'/exp OR osteoporosis:ti,ab,kw OR osteoporotic:ti,ab,kw OR 'neoplasm'/exp OR neoplas*:ti,ab,kw OR cancer*:ti,ab,kw OR tumor*:ti,ab,kw OR tumour*:ti,ab,kw OR carcinoma*:ti,ab,kw OR oncolog*:ti,ab,kw OR 'chronic kidney failure'/exp OR 'chronic renal insufficiency':ti,ab,kw OR 'chronic kidney insufficiency':ti,ab,kw OR 'chronic renal disease':ti,ab,kw OR 'chronic kidney disease':ti,ab,kw OR 'chronic renal insufficiencies':ti,ab,kw OR 'chronic renal diseases':ti,ab,kw OR 'chronic kidney failure':ti,ab,kw OR 'chronic renal failure':ti,ab,kw OR 'hemodialysis'/exp OR 'renal dialysis':ti,ab,kw OR 'kidney dialysis':ti,ab,kw OR hemodialysis:ti,ab,kw OR haemodialysis:ti,ab,kw OR 'hyperlipidemia'/exp OR hyperlipidemia:ti,ab,kw OR hyperlipidemias:ti,ab,kw OR hyperlipidemic:ti,ab,kw OR hyperlipemia:ti,ab,kw OR hyperlipemias:ti,ab,kw OR hyperlipemic:ti,ab,kw OR lipidemia:ti,ab,kw OR lipidemias:ti,ab,kw OR lipidemic:ti,ab,kw OR lipemia:ti,ab,kw OR lipemias:ti,ab,kw OR lipemic:ti,ab,kw OR 'cholesterol'/exp OR hypercholesterolemia:ti,ab,kw OR hyper-cholesterolemia:ti,ab,kw OR hypercholesterolemic:ti,ab,kw OR hyper-cholesterolemic:ti,ab,kw OR cholesterol:ti,ab,kw OR hypertriglyceridemia:ti,ab,kw OR hyper-triglyceridemia:ti,ab,kw OR

	hypertriglyceridemic:ti,ab,kw OR hyper-triglyceridemic:ti,ab,kw OR 'triacylglycerol'/exp OR triglycerid:ti,ab,kw OR triglycerids:ti,ab,kw)
#6	(**‘diabetes mellitus’/exp OR diabetes:ti,ab,kw OR diabetic:ti,ab,kw**) AND (‘heart failure’/exp OR ‘heart failure’:ti,ab,kw OR ‘heart failures’:ti,ab,kw OR ‘chronic obstructive lung disease’/exp OR ‘chronic obstructive pulmonary disease’:ti,ab,kw OR ‘chronic obstructive lung disease’:ti,ab,kw OR ‘chronic obstructive pulmonary diseases’:ti,ab,kw OR ‘chronic obstructive lung diseases’:ti,ab,kw OR COPD:ti,ab,kw OR ‘asthma’/exp OR asthma:ti,ab,kw OR asthmatic:ti,ab,kw OR ‘hypertension’/exp OR hypertension:ti,ab,kw OR ‘coronary artery disease’/exp OR ‘coronary artery disease’:ti,ab,kw OR ‘coronary atherosclerosis’:ti,ab,kw OR ‘coronary arteriosclerosis’:ti,ab,kw OR ‘coronary artery diseases’:ti,ab,kw OR ‘arthritis’/exp OR arthritis:ti,ab,kw OR arthritic:ti,ab,kw OR osteoarthritis:ti,ab,kw OR osteoarthritic:ti,ab,kw OR periarthritis:ti,ab,kw OR periarthritic:ti,ab,kw OR ‘osteoporosis’/exp OR osteoporosis:ti,ab,kw OR osteoporotic:ti,ab,kw OR ‘neoplasm’/exp OR neoplas*:ti,ab,kw OR cancer*:ti,ab,kw OR tumor*:ti,ab,kw OR tumour*:ti,ab,kw OR carcinoma*:ti,ab,kw OR oncolog*:ti,ab,kw OR ‘chronic kidney failure’/exp OR ‘chronic renal insufficiency’:ti,ab,kw OR ‘chronic kidney insufficiency’:ti,ab,kw OR ‘chronic renal disease’:ti,ab,kw OR ‘chronic kidney disease’:ti,ab,kw OR ‘chronic renal insufficiencies’:ti,ab,kw OR ‘chronic renal diseases’:ti,ab,kw OR ‘chronic kidney failure’:ti,ab,kw OR ‘chronic renal failure’:ti,ab,kw OR ‘hemodialysis’/exp OR ‘renal dialysis’:ti,ab,kw OR ‘kidney dialysis’:ti,ab,kw OR hemodialysis:ti,ab,kw OR haemodialysis:ti,ab,kw OR ‘hyperlipidemia’/exp OR hyperlipidemia:ti,ab,kw OR hyperlipidemias:ti,ab,kw OR hyperlipidemic:ti,ab,kw OR hyperlipemia:ti,ab,kw OR hyperlipemias:ti,ab,kw OR hyperlipemic:ti,ab,kw OR lipidemia:ti,ab,kw OR lipidemias:ti,ab,kw OR lipidemic:ti,ab,kw OR lipemia:ti,ab,kw OR lipemias:ti,ab,kw OR lipemic:ti,ab,kw OR ‘cholesterol’/exp OR hypercholesterolemia:ti,ab,kw OR hyper-cholesterolemia:ti,ab,kw OR hypercholesterolemic:ti,ab,kw OR hyper-cholesterolemic:ti,ab,kw OR cholesterol:ti,ab,kw OR hypertriglyceridemia:ti,ab,kw OR hyper-triglyceridemia:ti,ab,kw OR hypertriglyceridemic:ti,ab,kw OR hyper-triglyceridemic:ti,ab,kw OR 'triacylglycerol'/exp OR triglycerid:ti,ab,kw OR triglycerids:ti,ab,kw)
#7	(**‘hypertension’/exp OR hypertension:ti,ab,kw**) AND (‘heart failure’/exp OR ‘heart failure’:ti,ab,kw OR ‘heart failures’:ti,ab,kw OR ‘heart failures’:ti,ab,kw OR ‘chronic obstructive lung disease’/exp OR ‘chronic obstructive pulmonary disease’:ti,ab,kw OR ‘chronic obstructive lung disease’:ti,ab,kw OR ‘chronic obstructive pulmonary diseases’:ti,ab,kw OR ‘chronic obstructive lung diseases’:ti,ab,kw OR COPD:ti,ab,kw OR ‘asthma’/exp OR asthma:ti,ab,kw OR asthmatic:ti,ab,kw OR ‘diabetes mellitus’/exp OR diabetes:ti,ab,kw OR diabetic:ti,ab,kw OR ‘coronary artery disease’/exp OR ‘coronary artery disease’:ti,ab,kw OR ‘coronary atherosclerosis’:ti,ab,kw OR ‘coronary arteriosclerosis’:ti,ab,kw OR ‘coronary artery diseases’:ti,ab,kw OR ‘arthritis’/exp OR arthritis:ti,ab,kw OR arthritic:ti,ab,kw OR osteoarthritis:ti,ab,kw OR osteoarthritic:ti,ab,kw OR periarthritis:ti,ab,kw OR periarthritic:ti,ab,kw OR ‘osteoporosis’/exp OR osteoporosis:ti,ab,kw OR osteoporotic:ti,ab,kw OR ‘neoplasm’/exp OR neoplas*:ti,ab,kw OR cancer*:ti,ab,kw OR tumor*:ti,ab,kw OR tumour*:ti,ab,kw OR carcinoma*:ti,ab,kw OR oncolog*:ti,ab,kw OR ‘chronic kidney failure’/exp OR ‘chronic renal insufficiency’:ti,ab,kw OR ‘chronic kidney insufficiency’:ti,ab,kw OR ‘chronic renal disease’:ti,ab,kw OR ‘chronic kidney disease’:ti,ab,kw OR ‘chronic renal insufficiencies’:ti,ab,kw OR ‘chronic renal diseases’:ti,ab,kw OR ‘chronic kidney failure’:ti,ab,kw OR ‘chronic renal failure’:ti,ab,kw OR ‘hemodialysis’/exp OR ‘renal dialysis’:ti,ab,kw OR ‘kidney dialysis’:ti,ab,kw OR hemodialysis:ti,ab,kw OR haemodialysis:ti,ab,kw OR ‘hyperlipidemia’/exp OR hyperlipidemia:ti,ab,kw OR hyperlipidemias:ti,ab,kw OR hyperlipidemic:ti,ab,kw OR hyperlipemia:ti,ab,kw OR hyperlipemias:ti,ab,kw OR hyperlipemic:ti,ab,kw OR

	lipidemia:ti,ab,kw OR lipidemias:ti,ab,kw OR lipidemic:ti,ab,kw OR lipemia:ti,ab,kw OR lipemias:ti,ab,kw OR lipemic:ti,ab,kw OR ‘cholesterol’/exp OR hypercholesterolemia:ti,ab,kw OR hyper-cholesterolemia:ti,ab,kw OR hypercholesterolemic:ti,ab,kw OR hyper-cholesterolemic:ti,ab,kw OR cholesterol:ti,ab,kw OR hypertriglyceridemia:ti,ab,kw OR hyper-triglyceridemia:ti,ab,kw OR hypertriglyceridemic:ti,ab,kw OR hyper-triglyceridemic:ti,ab,kw OR 'triacylglycerol'/exp OR triglycerid:ti,ab,kw OR triglycerids:ti,ab,kw)
#8	(**‘coronary artery disease’/exp OR ‘coronary artery disease’:ti,ab,kw OR ‘coronary atherosclerosis’:ti,ab,kw OR ‘coronary arteriosclerosis’:ti,ab,kw OR ‘coronary artery diseases’:ti,ab,kw**) AND (‘heart failure’/exp OR ‘heart failure’:ti,ab,kw OR ‘heart failures’:ti,ab,kw OR ‘chronic obstructive lung disease’/exp OR ‘chronic obstructive pulmonary disease’:ti,ab,kw OR ‘chronic obstructive lung disease’:ti,ab,kw OR ‘chronic obstructive pulmonary diseases’:ti,ab,kw OR ‘chronic obstructive lung diseases’:ti,ab,kw OR COPD:ti,ab,kw OR ‘asthma’/exp OR asthma:ti,ab,kw OR asthmatic:ti,ab,kw OR ‘diabetes mellitus’/exp OR diabetes:ti,ab,kw OR diabetic:ti,ab,kw OR ‘hypertension’/exp OR hypertension:ti,ab,kw OR ‘arthritis’/exp OR arthritis:ti,ab,kw OR arthritic:ti,ab,kw OR osteoarthritis:ti,ab,kw OR osteoarthritic:ti,ab,kw OR periarthritis:ti,ab,kw OR periarthritic:ti,ab,kw OR ‘osteoporosis’/exp OR osteoporosis:ti,ab,kw OR osteoporotic:ti,ab,kw OR ‘neoplasm’/exp OR neoplas*:ti,ab,kw OR cancer*:ti,ab,kw OR tumor*:ti,ab,kw OR tumour*:ti,ab,kw OR carcinoma*:ti,ab,kw OR oncolog*:ti,ab,kw OR ‘chronic kidney failure’/exp OR ‘chronic renal insufficiency’:ti,ab,kw OR ‘chronic kidney insufficiency’:ti,ab,kw OR ‘chronic renal disease’:ti,ab,kw OR ‘chronic kidney disease’:ti,ab,kw OR ‘chronic renal insufficiencies’:ti,ab,kw OR ‘chronic renal diseases’:ti,ab,kw OR ‘chronic kidney failure’:ti,ab,kw OR ‘chronic renal failure’:ti,ab,kw OR ‘hemodialysis’/exp OR ‘renal dialysis’:ti,ab,kw OR ‘kidney dialysis’:ti,ab,kw OR hemodialysis:ti,ab,kw OR haemodialysis:ti,ab,kw OR ‘hyperlipidemia’/exp OR hyperlipidemia:ti,ab,kw OR hyperlipidemias:ti,ab,kw OR hyperlipidemic:ti,ab,kw OR hyperlipemia:ti,ab,kw OR hyperlipemias:ti,ab,kw OR hyperlipemic:ti,ab,kw OR lipidemia:ti,ab,kw OR lipidemias:ti,ab,kw OR lipidemic:ti,ab,kw OR lipemia:ti,ab,kw OR lipemias:ti,ab,kw OR lipemic:ti,ab,kw OR ‘cholesterol’/exp OR hypercholesterolemia:ti,ab,kw OR hyper-cholesterolemia:ti,ab,kw OR hypercholesterolemic:ti,ab,kw OR hyper-cholesterolemic:ti,ab,kw OR cholesterol:ti,ab,kw OR hypertriglyceridemia:ti,ab,kw OR hyper-triglyceridemia:ti,ab,kw OR hypertriglyceridemic:ti,ab,kw OR hyper-triglyceridemic:ti,ab,kw OR 'triacylglycerol'/exp OR triglycerid:ti,ab,kw OR triglycerids:ti,ab,kw)
#9	(**‘arthritis’/exp OR arthritis:ti,ab,kw OR arthritic:ti,ab,kw OR osteoarthritis:ti,ab,kw OR osteoarthritic:ti,ab,kw OR periarthritis:ti,ab,kw OR periarthritic:ti,ab,kw**) AND (‘heart failure’/exp OR ‘heart failure’:ti,ab,kw OR ‘heart failures’:ti,ab,kw OR ‘chronic obstructive lung disease’/exp OR ‘chronic obstructive pulmonary disease’:ti,ab,kw OR ‘chronic obstructive lung disease’:ti,ab,kw OR ‘chronic obstructive pulmonary diseases’:ti,ab,kw OR ‘chronic obstructive lung diseases’:ti,ab,kw OR COPD:ti,ab,kw OR ‘asthma’/exp OR asthma:ti,ab,kw OR asthmatic:ti,ab,kw OR ‘diabetes mellitus’/exp OR diabetes:ti,ab,kw OR diabetic:ti,ab,kw OR ‘hypertension’/exp OR hypertension:ti,ab,kw OR ‘coronary artery disease’/exp OR ‘coronary artery disease’:ti,ab,kw OR ‘coronary atherosclerosis’:ti,ab,kw OR ‘coronary arteriosclerosis’:ti,ab,kw OR ‘coronary artery diseases’:ti,ab,kw OR ‘osteoporosis’/exp OR osteoporosis:ti,ab,kw OR osteoporotic:ti,ab,kw OR ‘neoplasm’/exp OR neoplas*:ti,ab,kw OR cancer*:ti,ab,kw OR tumor*:ti,ab,kw OR tumour*:ti,ab,kw OR carcinoma*:ti,ab,kw OR oncolog*:ti,ab,kw OR ‘chronic kidney failure’/exp OR ‘chronic renal insufficiency’:ti,ab,kw OR ‘chronic kidney insufficiency’:ti,ab,kw OR ‘chronic renal disease’:ti,ab,kw OR ‘chronic kidney disease’:ti,ab,kw OR ‘chronic renal insufficiencies’:ti,ab,kw OR ‘chronic renal

	diseases':ti,ab,kw OR 'chronic kidney failure':ti,ab,kw OR 'chronic renal failure':ti,ab,kw OR 'hemodialysis'/exp OR 'renal dialysis':ti,ab,kw OR 'kidney dialysis':ti,ab,kw OR hemodialysis:ti,ab,kw OR haemodialysis:ti,ab,kw OR 'hyperlipidemia'/exp OR hyperlipidemia:ti,ab,kw OR hyperlipidemias:ti,ab,kw OR hyperlipidemic:ti,ab,kw OR hyperlipemia:ti,ab,kw OR hyperlipemias:ti,ab,kw OR hyperlipemic:ti,ab,kw OR lipidemia:ti,ab,kw OR lipidemias:ti,ab,kw OR lipidemic:ti,ab,kw OR lipemia:ti,ab,kw OR lipemias:ti,ab,kw OR lipemic:ti,ab,kw OR 'cholesterol'/exp OR hypercholesterolemia:ti,ab,kw OR hyper-cholesterolemia:ti,ab,kw OR hypercholesterolemic:ti,ab,kw OR hyper-cholesterolemic:ti,ab,kw OR cholesterol:ti,ab,kw OR hypertriglyceridemia:ti,ab,kw OR hyper-triglyceridemia:ti,ab,kw OR hypertriglyceridemic:ti,ab,kw OR hyper-triglyceridemic:ti,ab,kw OR 'triacylglycerol'/exp OR triglycerid:ti,ab,kw OR triglycerids:ti,ab,kw)
#10	(**'osteoporosis'/exp OR osteoporosis:ti,ab,kw OR osteoporotic:ti,ab,kw**) AND ('heart failure'/exp OR 'heart failure':ti,ab,kw OR 'heart failures':ti,ab,kw OR 'chronic obstructive lung disease'/exp OR 'chronic obstructive pulmonary disease':ti,ab,kw OR 'chronic obstructive lung disease':ti,ab,kw OR 'chronic obstructive pulmonary diseases':ti,ab,kw OR 'chronic obstructive lung diseases':ti,ab,kw OR COPD:ti,ab,kw OR 'asthma'/exp OR asthma:ti,ab,kw OR asthmatic:ti,ab,kw OR 'diabetes mellitus'/exp OR diabetes:ti,ab,kw OR diabetic:ti,ab,kw OR 'hypertension'/exp OR hypertension:ti,ab,kw OR 'coronary artery disease'/exp OR 'coronary artery disease':ti,ab,kw OR 'coronary atherosclerosis':ti,ab,kw OR 'coronary arteriosclerosis':ti,ab,kw OR 'coronary artery diseases':ti,ab,kw OR 'arthritis'/exp OR arthritis:ti,ab,kw OR arthritic:ti,ab,kw OR osteoarthritis:ti,ab,kw OR osteoarthritic:ti,ab,kw OR periarthritis:ti,ab,kw OR periarthritic:ti,ab,kw OR 'neoplasm'/exp OR neoplas*:ti,ab,kw OR cancer*:ti,ab,kw OR tumor*:ti,ab,kw OR tumour*:ti,ab,kw OR carcinoma*:ti,ab,kw OR oncolog*:ti,ab,kw OR 'chronic kidney failure'/exp OR 'chronic renal insufficiency':ti,ab,kw OR 'chronic kidney insufficiency':ti,ab,kw OR 'chronic renal disease':ti,ab,kw OR 'chronic kidney disease':ti,ab,kw OR 'chronic renal insufficiencies':ti,ab,kw OR 'chronic renal diseases':ti,ab,kw OR 'chronic kidney failure':ti,ab,kw OR 'chronic renal failure':ti,ab,kw OR 'hemodialysis'/exp OR 'renal dialysis':ti,ab,kw OR 'kidney dialysis':ti,ab,kw OR hemodialysis:ti,ab,kw OR haemodialysis:ti,ab,kw OR 'hyperlipidemia'/exp OR hyperlipidemia:ti,ab,kw OR hyperlipidemias:ti,ab,kw OR hyperlipidemic:ti,ab,kw OR hyperlipemia:ti,ab,kw OR hyperlipemias:ti,ab,kw OR hyperlipemic:ti,ab,kw OR lipidemia:ti,ab,kw OR lipidemias:ti,ab,kw OR lipidemic:ti,ab,kw OR lipemia:ti,ab,kw OR lipemias:ti,ab,kw OR lipemic:ti,ab,kw OR 'cholesterol'/exp OR hypercholesterolemia:ti,ab,kw OR hyper-cholesterolemia:ti,ab,kw OR hypercholesterolemic:ti,ab,kw OR hyper-cholesterolemic:ti,ab,kw OR cholesterol:ti,ab,kw OR hypertriglyceridemia:ti,ab,kw OR hyper-triglyceridemia:ti,ab,kw OR hypertriglyceridemic:ti,ab,kw OR hyper-triglyceridemic:ti,ab,kw OR 'triacylglycerol'/exp OR triglycerid:ti,ab,kw OR triglycerids:ti,ab,kw)
#11	(**'neoplasm'/exp OR neoplas*:ti,ab,kw OR cancer*:ti,ab,kw OR tumor*:ti,ab,kw OR tumour*:ti,ab,kw OR carcinoma*:ti,ab,kw OR oncolog*:ti,ab,kw**) AND ('heart failure'/exp OR 'heart failure':ti,ab,kw OR 'heart failures':ti,ab,kw OR 'chronic obstructive lung disease'/exp OR 'chronic obstructive pulmonary disease':ti,ab,kw OR 'chronic obstructive lung disease':ti,ab,kw OR 'chronic obstructive pulmonary diseases':ti,ab,kw OR 'chronic obstructive lung diseases':ti,ab,kw OR COPD:ti,ab,kw OR 'asthma'/exp OR asthma:ti,ab,kw OR asthmatic:ti,ab,kw OR 'diabetes mellitus'/exp OR diabetes:ti,ab,kw OR diabetic:ti,ab,kw OR 'hypertension'/exp OR hypertension:ti,ab,kw OR 'coronary artery disease'/exp OR 'coronary artery disease':ti,ab,kw OR 'coronary atherosclerosis':ti,ab,kw OR 'coronary arteriosclerosis':ti,ab,kw OR 'coronary artery diseases':ti,ab,kw OR 'arthritis'/exp OR arthritis:ti,ab,kw OR arthritic:ti,ab,kw OR osteoarthritis:ti,ab,kw OR

	osteoarthritic:ti,ab,kw OR periarthritis:ti,ab,kw OR periarthritic:ti,ab,kw OR 'osteoporosis'/exp OR osteoporosis:ti,ab,kw OR osteoporotic:ti,ab,kw OR 'chronic kidney failure'/exp OR 'chronic renal insufficiency':ti,ab,kw OR 'chronic kidney insufficiency':ti,ab,kw OR 'chronic renal disease':ti,ab,kw OR 'chronic kidney disease':ti,ab,kw OR 'chronic renal insufficiencies':ti,ab,kw OR 'chronic renal diseases':ti,ab,kw OR 'chronic kidney failure':ti,ab,kw OR 'chronic renal failure':ti,ab,kw OR 'hemodialysis'/exp OR 'renal dialysis':ti,ab,kw OR 'kidney dialysis':ti,ab,kw OR hemodialysis:ti,ab,kw OR haemodialysis:ti,ab,kw OR 'hyperlipidemia'/exp OR hyperlipidemia:ti,ab,kw OR hyperlipidemias:ti,ab,kw OR hyperlipidemic:ti,ab,kw OR hyperlipemia:ti,ab,kw OR hyperlipemias:ti,ab,kw OR hyperlipemic:ti,ab,kw OR lipidemia:ti,ab,kw OR lipidemias:ti,ab,kw OR lipidemic:ti,ab,kw OR lipemia:ti,ab,kw OR lipemias:ti,ab,kw OR lipemic:ti,ab,kw OR 'cholesterol'/exp OR hypercholesterolemia:ti,ab,kw OR hyper-cholesterolemia:ti,ab,kw OR hypercholesterolemic:ti,ab,kw OR hyper-cholesterolemic:ti,ab,kw OR cholesterol:ti,ab,kw OR hypertriglyceridemia:ti,ab,kw OR hyper-triglyceridemia:ti,ab,kw OR hypertriglyceridemic:ti,ab,kw OR hyper-triglyceridemic:ti,ab,kw OR 'triacylglycerol'/exp OR triglycerid:ti,ab,kw OR triglycerids:ti,ab,kw)
#12	(**'chronic kidney failure'/exp OR 'chronic renal insufficiency':ti,ab,kw OR 'chronic kidney insufficiency':ti,ab,kw OR 'chronic renal disease':ti,ab,kw OR 'chronic kidney disease':ti,ab,kw OR 'chronic renal insufficiencies':ti,ab,kw OR 'chronic renal diseases':ti,ab,kw OR 'chronic kidney diseases':ti,ab,kw OR 'chronic kidney failure':ti,ab,kw OR 'chronic renal failure':ti,ab,kw OR 'hemodialysis'/exp OR 'renal dialysis':ti,ab,kw OR 'kidney dialysis':ti,ab,kw OR hemodialysis:ti,ab,kw OR haemodialysis:ti,ab,kw**) AND ('heart failure'/exp OR 'heart failure':ti,ab,kw OR 'heart failures':ti,ab,kw OR 'chronic obstructive lung disease'/exp OR 'chronic obstructive pulmonary disease':ti,ab,kw OR 'chronic obstructive lung disease':ti,ab,kw OR 'chronic obstructive pulmonary diseases':ti,ab,kw OR 'chronic obstructive lung diseases':ti,ab,kw OR COPD:ti,ab,kw OR 'asthma'/exp OR asthma:ti,ab,kw OR asthmatic:ti,ab,kw OR 'diabetes mellitus'/exp OR diabetes:ti,ab,kw OR diabetic:ti,ab,kw OR 'hypertension'/exp OR hypertension:ti,ab,kw OR 'coronary artery disease'/exp OR 'coronary artery disease':ti,ab,kw OR 'coronary atherosclerosis':ti,ab,kw OR 'coronary arteriosclerosis':ti,ab,kw OR 'coronary artery diseases':ti,ab,kw OR 'arthritis'/exp OR arthritis:ti,ab,kw OR arthritic:ti,ab,kw OR osteoarthritis:ti,ab,kw OR osteoarthritic:ti,ab,kw OR periarthritis:ti,ab,kw OR periarthritic:ti,ab,kw OR 'osteoporosis'/exp OR osteoporosis:ti,ab,kw OR osteoporotic:ti,ab,kw OR 'neoplasm'/exp OR neoplas*:ti,ab,kw OR cancer*:ti,ab,kw OR tumor*:ti,ab,kw OR tumour*:ti,ab,kw OR carcinoma*:ti,ab,kw OR oncolog*:ti,ab,kw OR 'hyperlipidemia'/exp OR hyperlipidemia:ti,ab,kw OR hyperlipidemias:ti,ab,kw OR hyperlipidemic:ti,ab,kw OR hyperlipemia:ti,ab,kw OR hyperlipemias:ti,ab,kw OR hyperlipemic:ti,ab,kw OR lipidemia:ti,ab,kw OR lipidemias:ti,ab,kw OR lipidemic:ti,ab,kw OR lipemia:ti,ab,kw OR lipemias:ti,ab,kw OR lipemic:ti,ab,kw OR 'cholesterol'/exp OR hypercholesterolemia:ti,ab,kw OR hyper-cholesterolemia:ti,ab,kw OR hypercholesterolemic:ti,ab,kw OR hyper-cholesterolemic:ti,ab,kw OR cholesterol:ti,ab,kw OR hypertriglyceridemia:ti,ab,kw OR hyper-triglyceridemia:ti,ab,kw OR hypertriglyceridemic:ti,ab,kw OR hyper-triglyceridemic:ti,ab,kw OR 'triacylglycerol'/exp OR triglycerid:ti,ab,kw OR triglycerids:ti,ab,kw)
#13	(**'hyperlipidemia'/exp OR hyperlipidemia:ti,ab,kw OR hyperlipidemias:ti,ab,kw OR hyperlipidemic:ti,ab,kw OR hyperlipemia:ti,ab,kw OR hyperlipemias:ti,ab,kw OR hyperlipemic:ti,ab,kw OR lipidemia:ti,ab,kw OR lipidemias:ti,ab,kw OR lipidemic:ti,ab,kw OR lipemia:ti,ab,kw OR lipemias:ti,ab,kw OR lipemic:ti,ab,kw OR 'cholesterol'/exp OR hypercholesterolemia:ti,ab,kw OR hyper-cholesterolemia:ti,ab,kw**

	**OR hypercholesterolemic:ti,ab,kw OR hyper-cholesterolemic:ti,ab,kw OR cholesterol:ti,ab,kw OR hypertriglyceridemia:ti,ab,kw OR hyper-triglyceridemia:ti,ab,kw OR hypertriglyceridemic:ti,ab,kw OR hyper-triglyceridemic:ti,ab,kw OR 'triacylglycerol'/exp OR triglycerid:ti,ab,kw OR triglycerids:ti,ab,kw**) AND (‘heart failure’/exp OR ‘heart failure’:ti,ab,kw OR ‘heart failures’:ti,ab,kw OR ‘chronic obstructive lung disease’/exp OR ‘chronic obstructive pulmonary disease’:ti,ab,kw OR ‘chronic obstructive lung disease’:ti,ab,kw OR ‘chronic obstructive pulmonary diseases’:ti,ab,kw OR ‘chronic obstructive lung diseases’:ti,ab,kw OR COPD:ti,ab,kw OR ‘asthma’/exp OR asthma:ti,ab,kw OR asthmatic:ti,ab,kw OR ‘diabetes mellitus’/exp OR diabetes:ti,ab,kw OR diabetic:ti,ab,kw OR ‘hypertension’/exp OR hypertension:ti,ab,kw OR ‘coronary artery disease’/exp OR ‘coronary artery disease’:ti,ab,kw OR ‘coronary atherosclerosis’:ti,ab,kw OR ‘coronary arteriosclerosis’:ti,ab,kw OR ‘coronary artery diseases’:ti,ab,kw OR ‘arthritis’/exp OR arthritis:ti,ab,kw OR arthritic:ti,ab,kw OR osteoarthritis:ti,ab,kw OR osteoarthritic:ti,ab,kw OR periarthritis:ti,ab,kw OR periarthritic:ti,ab,kw OR ‘osteoporosis’/exp OR osteoporosis:ti,ab,kw OR osteoporotic:ti,ab,kw OR ‘neoplasm’/exp OR neoplas*:ti,ab,kw OR cancer*:ti,ab,kw OR tumor*:ti,ab,kw OR tumour*:ti,ab,kw OR carcinoma*:ti,ab,kw OR oncolog*:ti,ab,kw OR ‘chronic kidney failure’/exp OR ‘chronic renal insufficiency’:ti,ab,kw OR ‘chronic kidney insufficiency’:ti,ab,kw OR ‘chronic renal disease’:ti,ab,kw OR ‘chronic kidney disease’:ti,ab,kw OR ‘chronic renal insufficiencies’:ti,ab,kw OR ‘chronic renal diseases’:ti,ab,kw OR ‘chronic kidney failure’:ti,ab,kw OR ‘chronic renal failure’:ti,ab,kw OR ‘hemodialysis’/exp OR ‘renal dialysis’:ti,ab,kw OR ‘kidney dialysis’:ti,ab,kw OR hemodialysis:ti,ab,kw OR haemodialysis:ti,ab,kw)
#14	#2 OR #3 OR #4 OR #5 OR #6 OR #7 OR #8 OR #9 OR #10 OR #11 OR #12 OR #13
#15	'aged'/exp OR 'geriatric patient'/exp OR aging:ti,ab,kw OR older:ti,ab,kw OR elder*:ti,ab,kw OR geriatr*:ti,ab,kw
#16	#1 AND #14 AND #15

CINAHL

S1	(MH "Motivational Interviewing" OR (MH "Motivation+" AND MH "Patient Education+") OR TI “motivational interview*” OR TI “motivational technique*” OR TI “motivational counsel*” OR TI “motivational enhancement therap*” OR TI “motivational therap*” OR TI “motivational intervention*” OR TI “motivational strateg*” OR TI “motivational approach*” OR TI “motivational communicat*” OR TI “motivational language*” OR AB “motivational interview*” OR AB “motivational technique*” OR AB “motivational counsel*” OR AB “motivational enhancement therap*” OR AB “motivational therap*” OR AB “motivational intervention*” OR AB “motivational strateg*” OR AB “motivational approach*” OR AB “motivational communicat*” OR AB “motivational language*”)
S2	(MH "Polypharmacy+" OR TI polypharma* OR TI polymedicat* OR TI poly-pharma* OR TI poly-medicat* OR TI multimedicat* OR TI multi-medicat* OR TI plurimedicat* OR TI polypatholog* OR TI poly-patholog* OR TI pluripatholog* OR TI pluri-patholog* OR TI multipatholog* OR TI multi-patholog* OR AB polypharma* OR AB polymedicat* OR AB poly-pharma* OR AB poly-medicat* OR AB multimedicat* OR AB multi-medicat* OR AB plurimedicat* OR AB polypatholog* OR AB poly-patholog* OR AB pluripatholog* OR AB pluri-patholog* OR AB multipatholog* OR AB multi-patholog* OR MH "Comorbidity" OR TI comorbid* OR TI multimorbid* OR TI multi-morbid* OR TI plurimorbid* OR TI polymorbid* OR TI poly-morbid* OR TI concurrent* OR TI concomitant* OR TI coexist* OR TI co-exist* OR AB comorbid* OR AB multimorbid* OR AB multi-morbid* OR AB plurimorbid* OR AB polymorbid* OR AB poly-morbid* OR AB concurrent* OR AB concomitant* OR AB coexist* OR AB co-exist* OR ((MH "Chronic Disease+" OR TI diseases OR TI conditions OR TI disorders OR TI illnesses OR TI “health problems” OR AB diseases OR AB conditions OR AB disorders OR AB illnesses OR AB “health problems”) AND (TI multiple OR AB multiple)))
S3	**(MH "Heart Failure+" OR TI “heart failure” OR TI “heart failures” OR AB “heart failure” OR AB “heart failures”)** AND (MH "Pulmonary Disease, Chronic Obstructive+" OR TI “chronic obstructive pulmonary disease” OR TI “chronic obstructive lung disease” OR TI “chronic obstructive pulmonary diseases” OR TI “chronic obstructive lung diseases” OR TI "COPD" OR AB “chronic obstructive pulmonary disease” OR AB “chronic obstructive lung disease” OR AB “chronic obstructive pulmonary diseases” OR AB “chronic obstructive lung diseases” OR AB "COPD" OR MH "Asthma+" OR TI asthma OR TI asthmatic OR AB asthma OR AB asthmatic OR MH “Diabetes Mellitus+” OR TI diabetes OR TI diabetic OR AB diabetes OR AB diabetic OR MH “Hypertension+” OR TI hypertension OR AB hypertension OR MH “Coronary Arteriosclerosis” OR TI “coronary artery disease” OR TI “coronary atherosclerosis” OR TI “coronary arteriosclerosis” OR TI “coronary artery diseases” OR AB “coronary artery disease” OR AB “coronary atherosclerosis” OR AB “coronary arteriosclerosis” OR AB “coronary artery diseases” OR MH “Arthritis+” OR TI arthritis OR TI arthritic OR TI osteoarthritis OR TI osteoarthritic OR TI periarthritis OR TI periarthritic OR AB arthritis OR AB arthritic OR AB osteoarthritis OR AB osteoarthritic OR AB periarthritis OR AB periarthritic OR MH “Osteoporosis+” OR TI osteoporosis OR TI osteoporotic OR AB osteoporosis OR AB osteoporotic OR MH “Neoplasms+” OR TI neoplas* OR TI cancer* OR TI tumor* OR TI tumour* OR TI carcinoma* OR TI oncolog* OR AB neoplas* OR AB cancer* OR AB tumor* OR AB tumour* OR AB carcinoma* OR AB oncolog* OR MH “Renal Insufficiency, Chronic+” OR TI “chronic renal insufficiency” OR TI “chronic kidney insufficiency” OR TI “chronic renal disease” OR TI “chronic kidney disease” OR TI “chronic renal insufficiencies” OR TI “chronic renal diseases” OR TI “chronic kidney failure” OR TI “chronic renal failure” OR AB “chronic renal insufficiency” OR AB “chronic kidney insufficiency” OR AB “chronic renal disease” OR AB “chronic kidney disease” OR AB “chronic renal insufficiencies” OR AB “chronic renal diseases” OR AB “chronic kidney failure” OR AB “chronic renal failure”

	OR MH “Hemodialysis+” OR TI “renal dialysis” OR TI “kidney dialysis” OR TI hemodialysis OR TI haemodialysis OR AB “renal dialysis” OR AB “kidney dialysis” OR AB hemodialysis OR AB haemodialysis OR MH “Hyperlipidemia+” OR TI hyperlipidemia OR TI hyperlipidemias OR TI hyperlipidemic OR TI hyperlipemia OR TI hyperlipemias OR TI hyperlipemic OR TI lipidemia OR TI lipidemias OR TI lipidemic OR TI lipemia OR TI lipemias OR TI lipemic OR AB hyperlipidemia OR AB hyperlipidemias OR AB hyperlipidemic OR AB hyperlipemia OR AB hyperlipemias OR AB hyperlipemic OR AB lipidemia OR AB lipidemias OR AB lipidemic OR AB lipemia OR AB lipemias OR AB lipemic OR MH "Cholesterol+" OR TI hypercholesterolemia OR TI hyper-cholesterolemia OR TI hypercholesterolemic OR TI hyper-cholesterolemic OR TI cholesterol OR TI hypertriglyceridemia OR TI hyper-triglyceridemia OR TI hypertriglyceridemic OR TI hyper-triglyceridemic OR AB hypercholesterolemia OR AB hyper-cholesterolemia OR AB hypercholesterolemic OR AB hyper-cholesterolemic OR AB cholesterol OR AB hypertriglyceridemia OR AB hyper-triglyceridemia OR AB hypertriglyceridemic OR AB hyper-triglyceridemic OR MH "Triglycerides" OR TI triglycerid OR TI triglycerids OR AB triglycerid OR AB triglycerids)
S4	(**MH "Pulmonary Disease, Chronic Obstructive+" OR TI “chronic obstructive pulmonary disease” OR TI “chronic obstructive lung disease” OR TI “chronic obstructive pulmonary diseases” OR TI “chronic obstructive lung diseases” OR TI "COPD" OR AB “chronic obstructive pulmonary disease” OR AB “chronic obstructive lung disease” OR AB “chronic obstructive pulmonary diseases” OR AB “chronic obstructive lung diseases” OR AB "COPD"**) AND (MH "Heart Failure+" OR TI “heart failure” OR TI “heart failures” OR AB “heart failure” OR AB “heart failures” OR MH "Asthma+" OR TI asthma OR TI asthmatic OR AB asthma OR AB asthmatic OR MH “Diabetes Mellitus+” OR TI diabetes OR TI diabetic OR AB diabetes OR AB diabetic OR MH “Hypertension+” OR TI hypertension OR AB hypertension OR MH “Coronary Arteriosclerosis” OR TI “coronary artery disease” OR TI “coronary atherosclerosis” OR TI “coronary arteriosclerosis” OR TI “coronary artery diseases” OR AB “coronary artery disease” OR AB “coronary atherosclerosis” OR AB “coronary arteriosclerosis” OR AB “coronary artery diseases” OR MH “Arthritis+” OR TI arthritis OR TI arthritic OR TI osteoarthritis OR TI osteoarthritic OR TI periarthritis OR TI periarthritic OR AB arthritis OR AB arthritic OR AB osteoarthritis OR AB osteoarthritic OR AB periarthritis OR AB periarthritic OR MH “Osteoporosis+” OR TI osteoporosis OR TI osteoporotic OR AB osteoporosis OR AB osteoporotic OR MH “Neoplasms+” OR TI neoplas* OR TI cancer* OR TI tumor* OR TI tumour* OR TI carcinoma* OR TI oncolog* OR AB neoplas* OR AB cancer* OR AB tumor* OR AB tumour* OR AB carcinoma* OR AB oncolog* OR MH “Renal Insufficiency, Chronic+” OR TI “chronic renal insufficiency” OR TI “chronic kidney insufficiency” OR TI “chronic renal disease” OR TI “chronic kidney disease” OR TI “chronic renal insufficiencies” OR TI “chronic renal diseases” OR TI “chronic kidney failure” OR TI “chronic renal failure” OR AB “chronic renal insufficiency” OR AB “chronic kidney insufficiency” OR AB “chronic renal disease” OR AB “chronic kidney disease” OR AB “chronic renal insufficiencies” OR AB “chronic renal diseases” OR AB “chronic kidney failure” OR AB “chronic renal failure” OR MH “Hemodialysis+” OR TI “renal dialysis” OR TI “kidney dialysis” OR TI hemodialysis OR TI haemodialysis OR AB “renal dialysis” OR AB “kidney dialysis” OR AB hemodialysis OR AB haemodialysis OR MH “Hyperlipidemia+” OR TI hyperlipidemia OR TI hyperlipidemias OR TI hyperlipidemic OR TI hyperlipemia OR TI hyperlipemias OR TI hyperlipemic OR TI lipidemia OR TI lipidemias OR TI lipidemic OR TI lipemia OR TI lipemias OR TI lipemic OR AB hyperlipidemia OR AB hyperlipidemias OR AB hyperlipidemic OR AB hyperlipemia OR AB hyperlipemias OR AB hyperlipemic OR AB lipidemia OR AB lipidemias OR AB lipidemic OR AB lipemia OR AB lipemias OR AB lipemic OR MH "Cholesterol+" OR TI hypercholesterolemia OR TI hyper-cholesterolemia OR TI hypercholesterolemic OR TI

	hyper-cholesterolemic OR TI cholesterol OR TI hypertriglyceridemia OR TI hyper-triglyceridemia OR TI hypertriglyceridemic OR TI hyper-triglyceridemic OR AB hypercholesterolemia OR AB hyper-cholesterolemia OR AB hypercholesterolemic OR AB hyper-cholesterolemic OR AB cholesterol OR AB hypertriglyceridemia OR AB hyper-triglyceridemia OR AB hypertriglyceridemic OR AB hyper-triglyceridemic OR MH "Triglycerides" OR TI triglycerid OR TI triglycerids OR AB triglycerid OR AB triglycerids)
S5	(**MH "Asthma+" OR TI asthma OR TI asthmatic OR AB asthma OR AB asthmatic**) AND (MH "Heart Failure+" OR TI “heart failure” OR TI “heart failures” OR AB “heart failure” OR AB “heart failures” OR MH "Pulmonary Disease, Chronic Obstructive+" OR TI “chronic obstructive pulmonary disease” OR TI “chronic obstructive lung disease” OR TI “chronic obstructive pulmonary diseases” OR TI “chronic obstructive lung diseases” OR TI "COPD" OR AB “chronic obstructive pulmonary disease” OR AB “chronic obstructive lung disease” OR AB “chronic obstructive pulmonary diseases” OR AB “chronic obstructive lung diseases” OR AB "COPD" OR MH “Diabetes Mellitus+” OR TI diabetes OR TI diabetic OR AB diabetes OR AB diabetic OR MH “Hypertension+” OR TI hypertension OR AB hypertension OR MH “Coronary Arteriosclerosis” OR TI “coronary artery disease” OR TI “coronary atherosclerosis” OR TI “coronary arteriosclerosis” OR TI “coronary artery diseases” OR AB “coronary artery disease” OR AB “coronary atherosclerosis” OR AB “coronary arteriosclerosis” OR AB “coronary artery diseases” OR MH “Arthritis+” OR TI arthritis OR TI arthritic OR TI osteoarthritis OR TI osteoarthritic OR TI periarthritis OR TI periarthritic OR AB arthritis OR AB arthritic OR AB osteoarthritis OR AB osteoarthritic OR AB periarthritis OR AB periarthritic OR MH “Osteoporosis+” OR TI osteoporosis OR TI osteoporotic OR AB osteoporosis OR AB osteoporotic OR MH “Neoplasms+” OR TI neoplas* OR TI cancer* OR TI tumor* OR TI tumour* OR TI carcinoma* OR TI oncolog* OR AB neoplas* OR AB cancer* OR AB tumor* OR AB tumour* OR AB carcinoma* OR AB oncolog* OR MH “Renal Insufficiency, Chronic+” OR TI “chronic renal insufficiency” OR TI “chronic kidney insufficiency” OR TI “chronic renal disease” OR TI “chronic kidney disease” OR TI “chronic renal insufficiencies” OR TI “chronic renal diseases” OR TI “chronic kidney failure” OR TI “chronic renal failure” OR AB “chronic renal insufficiency” OR AB “chronic kidney insufficiency” OR AB “chronic renal disease” OR AB “chronic kidney disease” OR AB “chronic renal insufficiencies” OR AB “chronic renal diseases” OR AB “chronic kidney failure” OR AB “chronic renal failure” OR MH “Hemodialysis+” OR TI “renal dialysis” OR TI “kidney dialysis” OR TI hemodialysis OR TI haemodialysis OR AB “renal dialysis” OR AB “kidney dialysis” OR AB hemodialysis OR AB haemodialysis OR MH “Hyperlipidemia+” OR TI hyperlipidemia OR TI hyperlipidemias OR TI hyperlipidemic OR TI hyperlipemia OR TI hyperlipemias OR TI hyperlipemic OR TI lipidemia OR TI lipidemias OR TI lipidemic OR TI lipemia OR TI lipemias OR TI lipemic OR AB hyperlipidemia OR AB hyperlipidemias OR AB hyperlipidemic OR AB hyperlipemia OR AB hyperlipemias OR AB hyperlipemic OR AB lipidemia OR AB lipidemias OR AB lipidemic OR AB lipemia OR AB lipemias OR AB lipemic OR MH "Cholesterol+" OR TI hypercholesterolemia OR TI hyper-cholesterolemia OR TI hypercholesterolemic OR TI hyper-cholesterolemic OR TI cholesterol OR TI hypertriglyceridemia OR TI hyper-triglyceridemia OR TI hypertriglyceridemic OR TI hyper-triglyceridemic OR AB hypercholesterolemia OR AB hyper-cholesterolemia OR AB hypercholesterolemic OR AB hyper-cholesterolemic OR AB cholesterol OR AB hypertriglyceridemia OR AB hyper-triglyceridemia OR AB hypertriglyceridemic OR AB hyper-triglyceridemic OR MH "Triglycerides" OR TI triglycerid OR TI triglycerids OR AB triglycerid OR AB triglycerids)
S6	(**MH “Diabetes Mellitus+” OR TI diabetes OR TI diabetic OR AB diabetes OR AB diabetic**) AND (MH "Heart Failure+" OR TI “heart failure” OR TI “heart failures” OR AB “heart failure” OR AB “heart failures” OR MH "Pulmonary Disease, Chronic Obstructive+"

	OR TI “chronic obstructive pulmonary disease” OR TI “chronic obstructive lung disease” OR TI “chronic obstructive pulmonary diseases” OR TI “chronic obstructive lung diseases” OR TI "COPD" OR AB “chronic obstructive pulmonary disease” OR AB “chronic obstructive lung disease” OR AB “chronic obstructive pulmonary diseases” OR AB “chronic obstructive lung diseases” OR AB "COPD" OR MH "Asthma+" OR TI asthma OR TI asthmatic OR AB asthma OR AB asthmatic OR MH “Hypertension+” OR TI hypertension OR AB hypertension OR MH “Coronary Arteriosclerosis” OR TI “coronary artery disease” OR TI “coronary atherosclerosis” OR TI “coronary arteriosclerosis” OR TI “coronary artery diseases” OR AB “coronary artery disease” OR AB “coronary atherosclerosis” OR AB “coronary arteriosclerosis” OR AB “coronary artery diseases” OR MH “Arthritis+” OR TI arthritis OR TI arthritic OR TI osteoarthritis OR TI osteoarthritic OR TI periarthritis OR TI periarthritic OR AB arthritis OR AB arthritic OR AB osteoarthritis OR AB osteoarthritic OR AB periarthritis OR AB periarthritic OR MH “Osteoporosis+” OR TI osteoporosis OR TI osteoporotic OR AB osteoporosis OR AB osteoporotic OR MH “Neoplasms+” OR TI neoplas* OR TI cancer* OR TI tumor* OR TI tumour* OR TI carcinoma* OR TI oncolog* OR AB neoplas* OR AB cancer* OR AB tumor* OR AB tumour* OR AB carcinoma* OR AB oncolog* OR MH “Renal Insufficiency, Chronic+” OR TI “chronic renal insufficiency” OR TI “chronic kidney insufficiency” OR TI “chronic renal disease” OR TI “chronic kidney disease” OR TI “chronic renal insufficiencies” OR TI “chronic renal diseases” OR TI “chronic kidney failure” OR TI “chronic renal failure” OR AB “chronic renal insufficiency” OR AB “chronic kidney insufficiency” OR AB “chronic renal disease” OR AB “chronic kidney disease” OR AB “chronic renal insufficiencies” OR AB “chronic renal diseases” OR AB “chronic kidney failure” OR AB “chronic renal failure” OR MH “Hemodialysis+” OR TI “renal dialysis” OR TI “kidney dialysis” OR TI hemodialysis OR TI haemodialysis OR AB “renal dialysis” OR AB “kidney dialysis” OR AB hemodialysis OR AB haemodialysis OR MH “Hyperlipidemia+” OR TI hyperlipidemia OR TI hyperlipidemias OR TI hyperlipidemic OR TI hyperlipemia OR TI hyperlipemias OR TI hyperlipemic OR TI lipidemia OR TI lipidemias OR TI lipidemic OR TI lipemia OR TI lipemias OR TI lipemic OR AB hyperlipidemia OR AB hyperlipidemias OR AB hyperlipidemic OR AB hyperlipemia OR AB hyperlipemias OR AB hyperlipemic OR AB lipidemia OR AB lipidemias OR AB lipidemic OR AB lipemia OR AB lipemias OR AB lipemic OR MH "Cholesterol+" OR TI hypercholesterolemia OR TI hyper-cholesterolemia OR TI hypercholesterolemic OR TI hyper-cholesterolemic OR TI cholesterol OR TI hypertriglyceridemia OR TI hyper-triglyceridemia OR TI hypertriglyceridemic OR TI hyper-triglyceridemic OR AB hypercholesterolemia OR AB hyper-cholesterolemia OR AB hypercholesterolemic OR AB hyper-cholesterolemic OR AB cholesterol OR AB hypertriglyceridemia OR AB hyper-triglyceridemia OR AB hypertriglyceridemic OR AB hyper-triglyceridemic OR MH "Triglycerides" OR TI triglycerid OR TI triglycerids OR AB triglycerid OR AB triglycerids)
S7	(**MH “Hypertension+” OR TI hypertension OR AB hypertension**) AND (MH "Heart Failure+" OR TI “heart failure” OR TI “heart failures” OR AB “heart failure” OR AB “heart failures” OR MH "Pulmonary Disease, Chronic Obstructive+" OR TI “chronic obstructive pulmonary disease” OR TI “chronic obstructive lung disease” OR TI “chronic obstructive pulmonary diseases” OR TI “chronic obstructive lung diseases” OR TI "COPD" OR AB “chronic obstructive pulmonary disease” OR AB “chronic obstructive lung disease” OR AB “chronic obstructive pulmonary diseases” OR AB “chronic obstructive lung diseases” OR AB "COPD" OR MH "Asthma+" OR TI asthma OR TI asthmatic OR AB asthma OR AB asthmatic OR MH “Diabetes Mellitus+” OR TI diabetes OR TI diabetic OR AB diabetes OR AB diabetic OR MH “Coronary Arteriosclerosis” OR TI “coronary artery disease” OR TI “coronary atherosclerosis” OR TI “coronary arteriosclerosis” OR TI “coronary artery diseases” OR AB “coronary artery disease” OR AB “coronary atherosclerosis” OR AB “coronary arteriosclerosis” OR AB “coronary artery diseases” OR MH “Arthritis+” OR TI

	arthritis OR TI arthritic OR TI osteoarthritis OR TI osteoarthritic OR TI periarthritis OR TI periarthritic OR AB arthritis OR AB arthritic OR AB osteoarthritis OR AB osteoarthritic OR AB periarthritis OR AB periarthritic OR MH “Osteoporosis+” OR TI osteoporosis OR TI osteoporotic OR AB osteoporosis OR AB osteoporotic OR MH “Neoplasms+” OR TI neoplas* OR TI cancer* OR TI tumor* OR TI tumour* OR TI carcinoma* OR TI oncolog* OR AB neoplas* OR AB cancer* OR AB tumor* OR AB tumour* OR AB carcinoma* OR AB oncolog* OR MH “Renal Insufficiency, Chronic+” OR TI “chronic renal insufficiency” OR TI “chronic kidney insufficiency” OR TI “chronic renal disease” OR TI “chronic kidney disease” OR TI “chronic renal insufficiencies” OR TI “chronic renal diseases” OR TI “chronic kidney failure” OR TI “chronic renal failure” OR AB “chronic renal insufficiency” OR AB “chronic kidney insufficiency” OR AB “chronic renal disease” OR AB “chronic kidney disease” OR AB “chronic renal insufficiencies” OR AB “chronic renal diseases” OR AB “chronic kidney failure” OR AB “chronic renal failure” OR MH “Hemodialysis+” OR TI “renal dialysis” OR TI “kidney dialysis” OR TI hemodialysis OR TI haemodialysis OR AB “renal dialysis” OR AB “kidney dialysis” OR AB hemodialysis OR AB haemodialysis OR MH “Hyperlipidemia+” OR TI hyperlipidemia OR TI hyperlipidemias OR TI hyperlipidemic OR TI hyperlipemia OR TI hyperlipemias OR TI hyperlipemic OR TI lipidemia OR TI lipidemias OR TI lipidemic OR TI lipemia OR TI lipemias OR TI lipemic OR AB hyperlipidemia OR AB hyperlipidemias OR AB hyperlipidemic OR AB hyperlipemia OR AB hyperlipemias OR AB hyperlipemic OR AB lipidemia OR AB lipidemias OR AB lipidemic OR AB lipemia OR AB lipemias OR AB lipemic OR MH "Cholesterol+" OR TI hypercholesterolemia OR TI hyper-cholesterolemia OR TI hypercholesterolemic OR TI hyper-cholesterolemic OR TI cholesterol OR TI hypertriglyceridemia OR TI hyper-triglyceridemia OR TI hypertriglyceridemic OR TI hyper-triglyceridemic OR AB hypercholesterolemia OR AB hyper-cholesterolemia OR AB hypercholesterolemic OR AB hyper-cholesterolemic OR AB cholesterol OR AB hypertriglyceridemia OR AB hyper-triglyceridemia OR AB hypertriglyceridemic OR AB hyper-triglyceridemic OR MH "Triglycerides" OR TI triglycerid OR TI triglycerids OR AB triglycerid OR AB triglycerids)
S8	(**MH “Coronary Arteriosclerosis” OR TI “coronary artery disease” OR TI “coronary atherosclerosis” OR TI “coronary arteriosclerosis” OR TI “coronary artery diseases” OR AB “coronary artery disease” OR AB “coronary atherosclerosis” OR AB “coronary arteriosclerosis” OR AB “coronary artery diseases”**) AND (MH "Heart Failure+" OR TI “heart failure” OR TI “heart failures” OR AB “heart failure” OR AB “heart failures” OR MH "Pulmonary Disease, Chronic Obstructive+" OR TI “chronic obstructive pulmonary disease” OR TI “chronic obstructive lung disease” OR TI “chronic obstructive pulmonary diseases” OR TI “chronic obstructive lung diseases” OR TI "COPD" OR AB “chronic obstructive pulmonary disease” OR AB “chronic obstructive lung disease” OR AB “chronic obstructive pulmonary diseases” OR AB “chronic obstructive lung diseases” OR AB "COPD" OR MH "Asthma+" OR TI asthma OR TI asthmatic OR AB asthma OR AB asthmatic OR MH “Diabetes Mellitus+” OR TI diabetes OR TI diabetic OR AB diabetes OR AB diabetic OR MH “Hypertension+” OR TI hypertension OR AB hypertension OR MH “Arthritis+” OR TI arthritis OR TI arthritic OR TI osteoarthritis OR TI osteoarthritic OR TI periarthritis OR TI periarthritic OR AB arthritis OR AB arthritic OR AB osteoarthritis OR AB osteoarthritic OR AB periarthritis OR AB periarthritic OR MH “Osteoporosis+” OR TI osteoporosis OR TI osteoporotic OR AB osteoporosis OR AB osteoporotic OR MH “Neoplasms+” OR TI neoplas* OR TI cancer* OR TI tumor* OR TI tumour* OR TI carcinoma* OR TI oncolog* OR AB neoplas* OR AB cancer* OR AB tumor* OR AB tumour* OR AB carcinoma* OR AB oncolog* OR MH “Renal Insufficiency, Chronic+” OR TI “chronic renal insufficiency” OR TI “chronic kidney insufficiency” OR TI “chronic renal disease” OR TI “chronic kidney disease” OR TI “chronic renal insufficiencies” OR TI “chronic renal diseases” OR TI “chronic kidney failure” OR TI “chronic renal failure” OR

	AB “chronic renal insufficiency” OR AB “chronic kidney insufficiency” OR AB “chronic renal disease” OR AB “chronic kidney disease” OR AB “chronic renal insufficiencies” OR AB “chronic renal diseases” OR AB “chronic kidney failure” OR AB “chronic renal failure” OR MH “Hemodialysis+” OR TI “renal dialysis” OR TI “kidney dialysis” OR TI hemodialysis OR TI haemodialysis OR AB “renal dialysis” OR AB “kidney dialysis” OR AB hemodialysis OR AB haemodialysis OR MH “Hyperlipidemia+” OR TI hyperlipidemia OR TI hyperlipidemias OR TI hyperlipidemic OR TI hyperlipemia OR TI hyperlipemias OR TI hyperlipemic OR TI lipidemia OR TI lipidemias OR TI lipidemic OR TI lipemia OR TI lipemias OR TI lipemic OR AB hyperlipidemia OR AB hyperlipidemias OR AB hyperlipidemic OR AB hyperlipemia OR AB hyperlipemias OR AB hyperlipemic OR AB lipidemia OR AB lipidemias OR AB lipidemic OR AB lipemia OR AB lipemias OR AB lipemic OR MH "Cholesterol+" OR TI hypercholesterolemia OR TI hyper-cholesterolemia OR TI hypercholesterolemic OR TI hyper-cholesterolemic OR TI cholesterol OR TI hypertriglyceridemia OR TI hyper-triglyceridemia OR TI hypertriglyceridemic OR TI hyper-triglyceridemic OR AB hypercholesterolemia OR AB hyper-cholesterolemia OR AB hypercholesterolemic OR AB hyper-cholesterolemic OR AB cholesterol OR AB hypertriglyceridemia OR AB hyper-triglyceridemia OR AB hypertriglyceridemic OR AB hyper-triglyceridemic OR MH "Triglycerides" OR TI triglycerid OR TI triglycerids OR AB triglycerid OR AB triglycerids)
S9	(**MH “Arthritis+” OR TI arthritis OR TI arthritic OR TI osteoarthritis OR TI osteoarthritic OR TI periarthritis OR TI periarthritic OR AB arthritis OR AB arthritic OR AB osteoarthritis OR AB osteoarthritic OR AB periarthritis OR AB periarthritic**) AND (MH "Heart Failure+" OR TI “heart failure” OR TI “heart failures” OR AB “heart failure” OR AB “heart failures” OR MH "Pulmonary Disease, Chronic Obstructive+" OR TI “chronic obstructive pulmonary disease” OR TI “chronic obstructive lung disease” OR TI “chronic obstructive pulmonary diseases” OR TI “chronic obstructive lung diseases” OR TI "COPD" OR AB “chronic obstructive pulmonary disease” OR AB “chronic obstructive lung disease” OR AB “chronic obstructive pulmonary diseases” OR AB “chronic obstructive lung diseases” OR AB "COPD" OR MH "Asthma+" OR TI asthma OR TI asthmatic OR AB asthma OR AB asthmatic OR MH “Diabetes Mellitus+” OR TI diabetes OR TI diabetic OR AB diabetes OR AB diabetic OR MH “Hypertension+” OR TI hypertension OR AB hypertension OR MH “Coronary Arteriosclerosis” OR TI “coronary artery disease” OR TI “coronary atherosclerosis” OR TI “coronary arteriosclerosis” OR TI “coronary artery diseases” OR AB “coronary artery disease” OR AB “coronary atherosclerosis” OR AB “coronary arteriosclerosis” OR AB “coronary artery diseases” OR MH “Osteoporosis+” OR TI osteoporosis OR TI osteoporotic OR AB osteoporosis OR AB osteoporotic OR MH “Neoplasms+” OR TI neoplas* OR TI cancer* OR TI tumor* OR TI tumour* OR TI carcinoma* OR TI oncolog* OR AB neoplas* OR AB cancer* OR AB tumor* OR AB tumour* OR AB carcinoma* OR AB oncolog* OR MH “Renal Insufficiency, Chronic+” OR TI “chronic renal insufficiency” OR TI “chronic kidney insufficiency” OR TI “chronic renal disease” OR TI “chronic kidney disease” OR TI “chronic renal insufficiencies” OR TI “chronic renal diseases” OR TI “chronic kidney failure” OR TI “chronic renal failure” OR AB “chronic renal insufficiency” OR AB “chronic kidney insufficiency” OR AB “chronic renal disease” OR AB “chronic kidney disease” OR AB “chronic renal insufficiencies” OR AB “chronic renal diseases” OR AB “chronic kidney failure” OR AB “chronic renal failure” OR MH “Hemodialysis+” OR TI “renal dialysis” OR TI “kidney dialysis” OR TI hemodialysis OR TI haemodialysis OR AB “renal dialysis” OR AB “kidney dialysis” OR AB hemodialysis OR AB haemodialysis OR MH “Hyperlipidemia+” OR TI hyperlipidemia OR TI hyperlipidemias OR TI hyperlipidemic OR TI hyperlipemia OR TI hyperlipemias OR TI hyperlipemic OR TI lipidemia OR TI lipidemias OR TI lipidemic OR TI lipemia OR TI lipemias OR TI lipemic OR AB hyperlipidemia OR AB hyperlipidemias OR AB hyperlipidemic OR AB hyperlipemia OR

	AB hyperlipemias OR AB hyperlipemic OR AB lipidemia OR AB lipidemias OR AB lipidemic OR AB lipemia OR AB lipemias OR AB lipemic OR MH "Cholesterol+" OR TI hypercholesterolemia OR TI hyper-cholesterolemia OR TI hypercholesterolemic OR TI hyper-cholesterolemic OR TI cholesterol OR TI hypertriglyceridemia OR TI hyper-triglyceridemia OR TI hypertriglyceridemic OR TI hyper-triglyceridemic OR AB hypercholesterolemia OR AB hyper-cholesterolemia OR AB hypercholesterolemic OR AB hyper-cholesterolemic OR AB cholesterol OR AB hypertriglyceridemia OR AB hyper-triglyceridemia OR AB hypertriglyceridemic OR AB hyper-triglyceridemic OR MH "Triglycerides" OR TI triglycerid OR TI triglycerids OR AB triglycerid OR AB triglycerids)
S10	(**MH "Osteoporosis+" OR TI osteoporosis OR TI osteoporotic OR AB osteoporosis OR AB osteoporotic**) AND (MH "Heart Failure+" OR TI "heart failure" OR TI "heart failures" OR AB "heart failure" OR AB "heart failures" OR MH "Pulmonary Disease, Chronic Obstructive+" OR TI "chronic obstructive pulmonary disease" OR TI "chronic obstructive lung disease" OR TI "chronic obstructive pulmonary diseases" OR TI "chronic obstructive lung diseases" OR TI "COPD" OR AB "chronic obstructive pulmonary disease" OR AB "chronic obstructive lung disease" OR AB "chronic obstructive pulmonary diseases" OR AB "chronic obstructive lung diseases" OR AB "COPD" OR MH "Asthma+" OR TI asthma OR TI asthmatic OR AB asthma OR AB asthmatic OR MH "Diabetes Mellitus+" OR TI diabetes OR TI diabetic OR AB diabetes OR AB diabetic OR MH "Hypertension+" OR TI hypertension OR AB hypertension OR MH "Coronary Arteriosclerosis" OR TI "coronary artery disease" OR TI "coronary atherosclerosis" OR TI "coronary arteriosclerosis" OR TI "coronary artery diseases" OR AB "coronary artery disease" OR AB "coronary atherosclerosis" OR AB "coronary arteriosclerosis" OR AB "coronary artery diseases" OR MH "Arthritis+" OR TI arthritis OR TI arthritic OR TI osteoarthritis OR TI osteoarthritic OR TI periarthritis OR TI periarthritic OR AB arthritis OR AB arthritic OR AB osteoarthritis OR AB osteoarthritic OR AB periarthritis OR AB periarthritic OR MH "Neoplasms+" OR TI neoplas* OR TI cancer* OR TI tumor* OR TI tumour* OR TI carcinoma* OR TI oncolog* OR AB neoplas* OR AB cancer* OR AB tumor* OR AB tumour* OR AB carcinoma* OR AB oncolog* OR MH "Renal Insufficiency, Chronic+" OR TI "chronic renal insufficiency" OR TI "chronic kidney insufficiency" OR TI "chronic renal disease" OR TI "chronic kidney disease" OR TI "chronic renal insufficiencies" OR TI "chronic renal diseases" OR TI "chronic kidney failure" OR TI "chronic renal failure" OR AB "chronic renal insufficiency" OR AB "chronic kidney insufficiency" OR AB "chronic renal disease" OR AB "chronic kidney disease" OR AB "chronic renal insufficiencies" OR AB "chronic renal diseases" OR AB "chronic kidney failure" OR AB "chronic renal failure" OR MH "Hemodialysis+" OR TI "renal dialysis" OR TI "kidney dialysis" OR TI hemodialysis OR TI haemodialysis OR AB "renal dialysis" OR AB "kidney dialysis" OR AB hemodialysis OR AB haemodialysis OR MH "Hyperlipidemia+" OR TI hyperlipidemia OR TI hyperlipidemias OR TI hyperlipidemic OR TI hyperlipemia OR TI hyperlipemias OR TI hyperlipemic OR TI lipidemia OR TI lipidemias OR TI lipidemic OR TI lipemia OR TI lipemias OR TI lipemic OR AB hyperlipidemia OR AB hyperlipidemias OR AB hyperlipidemic OR AB hyperlipemia OR AB hyperlipemias OR AB hyperlipemic OR AB lipidemia OR AB lipidemias OR AB lipidemic OR AB lipemia OR AB lipemias OR AB lipemic OR MH "Cholesterol+" OR TI hypercholesterolemia OR TI hyper-cholesterolemia OR TI hypercholesterolemic OR TI hyper-cholesterolemic OR TI cholesterol OR TI hypertriglyceridemia OR TI hyper-triglyceridemia OR TI hypertriglyceridemic OR TI hyper-triglyceridemic OR AB hypercholesterolemia OR AB hyper-cholesterolemia OR AB hypercholesterolemic OR AB hyper-cholesterolemic OR AB cholesterol OR AB hypertriglyceridemia OR AB hyper-triglyceridemia OR AB hypertriglyceridemic OR AB hyper-triglyceridemic OR MH "Triglycerides" OR TI triglycerid OR TI triglycerids OR AB triglycerid OR AB triglycerids)

S11	(**MH “Neoplasms+” OR TI neoplas* OR TI cancer* OR TI tumor* OR TI tumour* OR TI carcinoma* OR TI oncolog* OR AB neoplas* OR AB cancer* OR AB tumor* OR AB tumour* OR AB carcinoma* OR AB oncolog***) AND (MH "Heart Failure+" OR TI “heart failure” OR TI “heart failures” OR AB “heart failure” OR AB “heart failures” OR MH "Pulmonary Disease, Chronic Obstructive+" OR TI “chronic obstructive pulmonary disease” OR TI “chronic obstructive lung disease” OR TI “chronic obstructive pulmonary diseases” OR TI “chronic obstructive lung diseases” OR TI "COPD" OR AB “chronic obstructive pulmonary disease” OR AB “chronic obstructive lung disease” OR AB “chronic obstructive pulmonary diseases” OR AB “chronic obstructive lung diseases” OR AB "COPD" OR MH "Asthma+" OR TI asthma OR TI asthmatic OR AB asthma OR AB asthmatic OR MH “Diabetes Mellitus+” OR TI diabetes OR TI diabetic OR AB diabetes OR AB diabetic OR MH “Hypertension+” OR TI hypertension OR AB hypertension OR MH “Coronary Arteriosclerosis” OR TI “coronary artery disease” OR TI “coronary atherosclerosis” OR TI “coronary arteriosclerosis” OR TI “coronary artery diseases” OR AB “coronary artery disease” OR AB “coronary atherosclerosis” OR AB “coronary arteriosclerosis” OR AB “coronary artery diseases” OR MH “Arthritis+” OR TI arthritis OR TI arthritic OR TI osteoarthritis OR TI osteoarthritic OR TI periarthritis OR TI periarthritic OR AB arthritis OR AB arthritic OR AB osteoarthritis OR AB osteoarthritic OR AB periarthritis OR AB periarthritic OR MH “Osteoporosis+” OR TI osteoporosis OR TI osteoporotic OR AB osteoporosis OR AB osteoporotic OR MH “Renal Insufficiency, Chronic+” OR TI “chronic renal insufficiency” OR TI “chronic kidney insufficiency” OR TI “chronic renal disease” OR TI “chronic kidney disease” OR TI “chronic renal insufficiencies” OR TI “chronic renal diseases” OR TI “chronic kidney failure” OR TI “chronic renal failure” OR AB “chronic renal insufficiency” OR AB “chronic kidney insufficiency” OR AB “chronic renal disease” OR AB “chronic kidney disease” OR AB “chronic renal insufficiencies” OR AB “chronic renal diseases” OR AB “chronic kidney failure” OR AB “chronic renal failure” OR MH “Hemodialysis+” OR TI “renal dialysis” OR TI “kidney dialysis” OR TI hemodialysis OR TI haemodialysis OR AB “renal dialysis” OR AB “kidney dialysis” OR AB hemodialysis OR AB haemodialysis OR MH “Hyperlipidemia+” OR TI hyperlipidemia OR TI hyperlipidemias OR TI hyperlipidemic OR TI hyperlipemia OR TI hyperlipemias OR TI hyperlipemic OR TI lipidemia OR TI lipidemias OR TI lipidemic OR TI lipemia OR TI lipemias OR TI lipemic OR AB hyperlipidemia OR AB hyperlipidemias OR AB hyperlipidemic OR AB hyperlipemia OR AB hyperlipemias OR AB hyperlipemic OR AB lipidemia OR AB lipidemias OR AB lipidemic OR AB lipemia OR AB lipemias OR AB lipemic OR MH "Cholesterol+" OR TI hypercholesterolemia OR TI hyper-cholesterolemia OR TI hypercholesterolemic OR TI hyper-cholesterolemic OR TI cholesterol OR TI hypertriglyceridemia OR TI hyper-triglyceridemia OR TI hypertriglyceridemic OR TI hyper-triglyceridemic OR AB hypercholesterolemia OR AB hyper-cholesterolemia OR AB hypercholesterolemic OR AB hyper-cholesterolemic OR AB cholesterol OR AB hypertriglyceridemia OR AB hyper-triglyceridemia OR AB hypertriglyceridemic OR AB hyper-triglyceridemic OR MH "Triglycerides" OR TI triglycerid OR TI triglycerids OR AB triglycerid OR AB triglycerids)
S12	(**MH “Renal Insufficiency, Chronic+” OR TI “chronic renal insufficiency” OR TI “chronic kidney insufficiency” OR TI “chronic renal disease” OR TI “chronic kidney disease” OR TI “chronic renal insufficiencies” OR TI “chronic renal diseases” OR TI “chronic kidney failure” OR TI “chronic renal failure” OR AB “chronic renal insufficiency” OR AB “chronic kidney insufficiency” OR AB “chronic renal disease” OR AB “chronic kidney disease” OR AB “chronic renal insufficiencies” OR AB “chronic renal diseases” OR AB “chronic kidney failure” OR AB “chronic renal failure” OR MH “Hemodialysis+” OR TI “renal dialysis” OR TI “kidney dialysis” OR TI hemodialysis OR TI haemodialysis OR AB “renal dialysis” OR AB “kidney dialysis” OR AB hemodialysis OR AB haemodialysis**) AND (MH "Heart Failure+" OR TI “heart

	failure" OR TI "heart failures" OR AB "heart failure" OR AB "heart failures" OR MH "Pulmonary Disease, Chronic Obstructive+" OR TI "chronic obstructive pulmonary disease" OR TI "chronic obstructive lung disease" OR TI "chronic obstructive pulmonary diseases" OR TI "chronic obstructive lung diseases" OR TI "COPD" OR AB "chronic obstructive pulmonary disease" OR AB "chronic obstructive lung disease" OR AB "chronic obstructive pulmonary diseases" OR AB "chronic obstructive lung diseases" OR AB "COPD" OR MH "Asthma+" OR TI asthma OR TI asthmatic OR AB asthma OR AB asthmatic OR MH "Diabetes Mellitus+" OR TI diabetes OR TI diabetic OR AB diabetes OR AB diabetic OR MH "Hypertension+" OR TI hypertension OR AB hypertension OR MH "Coronary Arteriosclerosis" OR TI "coronary artery disease" OR TI "coronary atherosclerosis" OR TI "coronary arteriosclerosis" OR TI "coronary artery diseases" OR AB "coronary artery disease" OR AB "coronary atherosclerosis" OR AB "coronary arteriosclerosis" OR AB "coronary artery diseases" OR MH "Arthritis+" OR TI arthritis OR TI arthritic OR TI osteoarthritis OR TI osteoarthritic OR TI periarthritis OR TI periarthritic OR AB arthritis OR AB arthritic OR AB osteoarthritis OR AB osteoarthritic OR AB periarthritis OR AB periarthritic OR MH "Osteoporosis+" OR TI osteoporosis OR TI osteoporotic OR AB osteoporosis OR AB osteoporotic OR MH "Neoplasms+" OR TI neoplas* OR TI cancer* OR TI tumor* OR TI tumour* OR TI carcinoma* OR TI oncolog* OR AB neoplas* OR AB cancer* OR AB tumor* OR AB tumour* OR AB carcinoma* OR AB oncolog* OR MH "Hyperlipidemia+" OR TI hyperlipidemia OR TI hyperlipidemias OR TI hyperlipidemic OR TI hyperlipemia OR TI hyperlipemias OR TI hyperlipemic OR TI lipidemia OR TI lipidemias OR TI lipidemic OR TI lipemia OR TI lipemias OR TI lipemic OR AB hyperlipidemia OR AB hyperlipidemias OR AB hyperlipidemic OR AB hyperlipemia OR AB hyperlipemias OR AB hyperlipemic OR AB lipidemia OR AB lipidemias OR AB lipidemic OR AB lipemia OR AB lipemias OR AB lipemic OR MH "Cholesterol+" OR TI hypercholesterolemia OR TI hyper-cholesterolemia OR TI hypercholesterolemic OR TI hyper-cholesterolemic OR TI cholesterol OR TI hypertriglyceridemia OR TI hyper-triglyceridemia OR TI hypertriglyceridemic OR TI hyper-triglyceridemic OR AB hypercholesterolemia OR AB hyper-cholesterolemia OR AB hypercholesterolemic OR AB hyper-cholesterolemic OR AB cholesterol OR AB hypertriglyceridemia OR AB hyper-triglyceridemia OR AB hypertriglyceridemic OR AB hyper-triglyceridemic OR MH "Triglycerides" OR TI triglycerid OR TI triglycerids OR AB triglycerid OR AB triglycerids)
S13	(**MH "Hyperlipidemia+" OR TI hyperlipidemia OR TI hyperlipidemias OR TI hyperlipidemic OR TI hyperlipemia OR TI hyperlipemias OR TI hyperlipemic OR TI lipidemia OR TI lipidemias OR TI lipidemic OR TI lipemia OR TI lipemias OR TI lipemic OR AB hyperlipidemia OR AB hyperlipidemias OR AB hyperlipidemic OR AB hyperlipemia OR AB hyperlipemias OR AB hyperlipemic OR AB lipidemia OR AB lipidemias OR AB lipidemic OR AB lipemia OR AB lipemias OR AB lipemic OR MH "Cholesterol+" OR TI hypercholesterolemia OR TI hyper-cholesterolemia OR TI hypercholesterolemic OR TI hyper-cholesterolemic OR TI cholesterol OR TI hypertriglyceridemia OR TI hyper-triglyceridemia OR TI hypertriglyceridemic OR TI hyper-triglyceridemic OR AB hypercholesterolemia OR AB hyper-cholesterolemia OR AB hypercholesterolemic OR AB hyper-cholesterolemic OR AB cholesterol OR AB hypertriglyceridemia OR AB hyper-triglyceridemia OR AB hypertriglyceridemic OR AB hyper-triglyceridemic OR MH "Triglycerides" OR TI triglycerid OR TI triglycerids OR AB triglycerid OR AB triglycerids**) AND (MH "Heart Failure+" OR TI "heart failure" OR TI "heart failures" OR AB "heart failure" OR AB "heart failures" OR MH "Pulmonary Disease, Chronic Obstructive+" OR TI "chronic obstructive pulmonary disease" OR TI "chronic obstructive lung disease" OR TI "chronic obstructive pulmonary diseases" OR TI "chronic obstructive lung diseases" OR TI "COPD" OR AB "chronic obstructive pulmonary disease" OR AB "chronic obstructive lung disease" OR AB "chronic obstructive pulmonary diseases" OR AB "chronic obstructive lung diseases" OR AB

	"COPD" OR MH "Asthma+" OR TI asthma OR TI asthmatic OR AB asthma OR AB asthmatic OR MH “Diabetes Mellitus+” OR TI diabetes OR TI diabetic OR AB diabetes OR AB diabetic OR MH “Hypertension+” OR TI hypertension OR AB hypertension OR MH “Coronary Arteriosclerosis” OR TI “coronary artery disease” OR TI “coronary atherosclerosis” OR TI “coronary arteriosclerosis” OR TI “coronary artery diseases” OR AB “coronary artery disease” OR AB “coronary atherosclerosis” OR AB “coronary arteriosclerosis” OR AB “coronary artery diseases” OR MH “Arthritis+” OR TI arthritis OR TI arthritic OR TI osteoarthritis OR TI osteoarthritic OR TI periarthritis OR TI periarthritic OR AB arthritis OR AB arthritic OR AB osteoarthritis OR AB osteoarthritic OR AB periarthritis OR AB periarthriticOR MH “Osteoporosis+” OR TI osteoporosis OR TI osteoporotic OR AB osteoporosis OR AB osteoporotic OR MH “Neoplasms+” OR TI neoplas* OR TI cancer* OR TI tumor* OR TI tumour* OR TI carcinoma* OR TI oncolog* OR AB neoplas* OR AB cancer* OR AB tumor* OR AB tumour* OR AB carcinoma* OR AB oncolog* OR MH “Renal Insufficiency, Chronic+” OR TI “chronic renal insufficiency” OR TI “chronic kidney insufficiency” OR TI “chronic renal disease” OR TI “chronic kidney disease” OR TI “chronic renal insufficiencies” OR TI “chronic renal diseases” OR TI “chronic kidney failure” OR TI “chronic renal failure” OR AB “chronic renal insufficiency” OR AB “chronic kidney insufficiency” OR AB “chronic renal disease” OR AB “chronic kidney disease” OR AB “chronic renal insufficiencies” OR AB “chronic renal diseases” OR AB “chronic kidney failure” OR AB “chronic renal failure” OR MH “Hemodialysis+” OR TI “renal dialysis” OR TI “kidney dialysis” OR TI hemodialysis OR TI haemodialysis OR AB “renal dialysis” OR AB “kidney dialysis” OR AB hemodialysis OR AB haemodialysis)
**S14**	**S2 OR S3 OR S4 OR S5 OR S6 OR S7 OR S8 OR S9 OR S10 OR S11 OR S12 OR S13**
S15	MH "Aged+" OR TI aging OR TI older OR TI elder* OR TI geriatr* OR AB aging OR AB older OR AB elder* OR AB geriatr*
**S16**	**S1 AND S14 AND S15**

PsycINFO (EBSCOhost)

S1	(DE "Motivational Interviewing" OR (DE "Motivation" OR DE "Motivation Training" AND DE "Client Education") OR TI "motivational interview*" OR TI "motivational technique*" OR TI "motivational counsel*" OR TI "motivational enhancement therap*" OR TI "motivational therap*" OR TI "motivational intervention*" OR TI "motivational strateg*" OR TI "motivational approach*" OR TI "motivational communicat*" OR TI "motivational language*" OR AB "motivational interview*" OR AB "motivational technique*" OR AB "motivational counsel*" OR AB "motivational enhancement therap*" OR AB "motivational therap*" OR AB "motivational intervention*" OR AB "motivational strateg*" OR AB "motivational approach*" OR AB "motivational communicat*" OR AB "motivational language*")
S2	(DE "Polypharmacy" OR TI polypharma* OR TI polymedicat* OR TI poly-pharma* OR TI poly-medicat* OR TI multimedicat* OR TI multi-medicat* OR TI plurimedicat* OR TI polypatholog* OR TI poly-patholog* OR TI pluripatholog* OR TI pluri-patholog* OR TI multipatholog* OR TI multi-patholog* OR AB polypharma* OR AB polymedicat* OR AB poly-pharma* OR AB poly-medicat* OR AB multimedicat* OR AB multi-medicat* OR AB plurimedicat* OR AB polypatholog* OR AB poly-patholog* OR AB pluripatholog* OR AB pluri-patholog* OR AB multipatholog* OR AB multi-patholog* OR DE "Comorbidity" OR TI comorbid* OR TI multimorbid* OR TI multi-morbid* OR TI plurimorbid* OR TI polymorbid* OR TI poly-morbid* OR TI concurrent* OR TI concomitant* OR TI coexist* OR TI co-exist* OR AB comorbid* OR AB multimorbid* OR AB multi-morbid* OR AB plurimorbid* OR AB polymorbid* OR AB poly-morbid* OR AB concurrent* OR AB concomitant* OR AB coexist* OR AB co-exist* OR ((DE "Chronic Illness" OR DE "Chronicity (Disorders)" OR TI diseases OR TI conditions OR TI disorders OR TI illnesses OR TI "health problems" OR AB diseases OR AB conditions OR AB disorders OR AB illnesses OR AB "health problems") AND (TI multiple OR AB multiple)))
S3	**(TI "heart failure" OR TI "heart failures" OR AB "heart failure" OR AB "heart failures")** AND (DE "Chronic Obstructive Pulmonary Disease" OR TI "chronic obstructive pulmonary disease" OR TI "chronic obstructive lung disease" OR TI "chronic obstructive pulmonary diseases" OR TI "chronic obstructive lung diseases" OR TI "COPD" OR AB "chronic obstructive pulmonary disease" OR AB "chronic obstructive lung disease" OR AB "chronic obstructive pulmonary diseases" OR AB "chronic obstructive lung diseases" OR AB "COPD" OR DE "Asthma" OR TI asthma OR TI asthmatic OR AB asthma OR AB asthmatic OR DE "Diabetes Mellitus" OR DE "Type 2 Diabetes" OR TI diabetes OR TI diabetic OR AB diabetes OR AB diabetic OR DE "Hypertension" OR TI hypertension OR AB hypertension OR DE "Coronary Heart Disease" OR TI "coronary artery disease" OR TI "coronary atherosclerosis" OR TI "coronary arteriosclerosis" OR TI "coronary artery diseases" OR AB "coronary artery disease" OR AB "coronary atherosclerosis" OR AB "coronary arteriosclerosis" OR AB "coronary artery diseases" OR DE "Arthritis" OR DE "Rheumatoid Arthritis" OR TI arthritis OR TI arthritic OR TI osteoarthritis OR TI osteoarthritic OR TI periarthritis OR TI periarthritic OR AB arthritis OR AB arthritic OR AB osteoarthritis OR AB osteoarthritic OR AB periarthritis OR AB periarthritic OR DE "Osteoporosis" OR TI osteoporosis OR TI osteoporotic OR AB osteoporosis OR AB osteoporotic OR DE "Neoplasms" OR DE "Breast Neoplasms" OR DE "Endocrine Neoplasms" OR DE "Leukemias" OR DE "Melanoma" OR DE "Metastasis" OR DE "Nervous System Neoplasms" OR TI neoplas* OR TI cancer* OR TI tumor* OR TI tumour* OR TI carcinoma* OR TI oncolog* OR AB neoplas* OR AB cancer* OR AB tumor* OR AB tumour* OR AB carcinoma* OR AB oncolog* OR TI "chronic renal insufficiency" OR TI "chronic kidney insufficiency" OR TI "chronic renal disease" OR TI "chronic kidney disease" OR TI "chronic renal insufficiencies" OR TI "chronic renal diseases" OR TI "chronic kidney failure" OR TI "chronic renal failure" OR AB "chronic renal insufficiency"

	OR AB “chronic kidney insufficiency” OR AB “chronic renal disease” OR AB “chronic kidney disease” OR AB “chronic renal insufficiencies” OR AB “chronic renal diseases” OR AB “chronic kidney failure” OR AB “chronic renal failure” OR DE "Hemodialysis" OR TI “renal dialysis” OR TI “kidney dialysis” OR TI hemodialysis OR TI haemodialysis OR AB “renal dialysis” OR AB “kidney dialysis” OR AB hemodialysis OR AB haemodialysis OR TI hyperlipidemia OR TI hyperlipidemias OR TI hyperlipidemic OR TI hyperlipemia OR TI hyperlipemias OR TI hyperlipemic OR TI lipidemia OR TI lipidemias OR TI lipidemic OR TI lipemia OR TI lipemias OR TI lipemic OR AB hyperlipidemia OR AB hyperlipidemias OR AB hyperlipidemic OR AB hyperlipemia OR AB hyperlipemias OR AB hyperlipemic OR AB lipidemia OR AB lipidemias OR AB lipidemic OR AB lipemia OR AB lipemias OR AB lipemic OR DE "Cholesterol" OR TI hypercholesterolemia OR TI hyper-cholesterolemia OR TI hypercholesterolemic OR TI hyper-cholesterolemic OR TI cholesterol OR TI hypertriglyceridemia OR TI hyper-triglyceridemia OR TI hypertriglyceridemic OR TI hyper-triglyceridemic OR AB hypercholesterolemia OR AB hyper-cholesterolemia OR AB hypercholesterolemic OR AB hyper-cholesterolemic OR AB cholesterol OR AB hypertriglyceridemia OR AB hyper-triglyceridemia OR AB hypertriglyceridemic OR AB hyper-triglyceridemic OR TI triglycerid OR TI triglycerids OR AB triglycerid OR AB triglycerids)
S4	(**DE "Chronic Obstructive Pulmonary Disease" OR TI “chronic obstructive pulmonary disease” OR TI “chronic obstructive lung disease” OR TI “chronic obstructive pulmonary diseases” OR TI “chronic obstructive lung diseases” OR TI "COPD" OR AB “chronic obstructive pulmonary disease” OR AB “chronic obstructive lung disease” OR AB “chronic obstructive pulmonary diseases” OR AB “chronic obstructive lung diseases” OR AB "COPD"**) AND (TI “heart failure” OR TI “heart failures” OR AB “heart failure” OR AB “heart failures” OR DE "Asthma" OR TI asthma OR TI asthmatic OR AB asthma OR AB asthmatic OR DE "Diabetes Mellitus" OR DE "Type 2 Diabetes" OR TI diabetes OR TI diabetic OR AB diabetes OR AB diabetic OR DE "Hypertension" OR TI hypertension OR AB hypertension OR DE "Coronary Heart Disease" OR TI “coronary artery disease” OR TI “coronary atherosclerosis” OR TI “coronary arteriosclerosis” OR TI “coronary artery diseases” OR AB “coronary artery disease” OR AB “coronary atherosclerosis” OR AB “coronary arteriosclerosis” OR AB “coronary artery diseases” OR DE "Arthritis" OR DE "Rheumatoid Arthritis" OR TI arthritis OR TI arthritic OR TI osteoarthritis OR TI osteoarthritic OR TI periarthritis OR TI periarthritic OR AB arthritis OR AB arthritic OR AB osteoarthritis OR AB osteoarthritic OR AB periarthritis OR AB periarthritic OR DE "Osteoporosis" OR TI osteoporosis OR TI osteoporotic OR AB osteoporosis OR AB osteoporotic OR DE "Neoplasms" OR DE "Breast Neoplasms" OR DE "Endocrine Neoplasms" OR DE "Leukemias" OR DE "Melanoma" OR DE "Metastasis" OR DE "Nervous System Neoplasms" OR TI neoplas* OR TI cancer* OR TI tumor* OR TI tumour* OR TI carcinoma* OR TI oncolog* OR AB neoplas* OR AB cancer* OR AB tumor* OR AB tumour* OR AB carcinoma* OR AB oncolog* OR TI “chronic renal insufficiency” OR TI “chronic kidney insufficiency” OR TI “chronic renal disease” OR TI “chronic kidney disease” OR TI “chronic renal insufficiencies” OR TI “chronic renal diseases” OR TI “chronic kidney failure” OR TI “chronic renal failure” OR AB “chronic renal insufficiency” OR AB “chronic kidney insufficiency” OR AB “chronic renal disease” OR AB “chronic kidney disease” OR AB “chronic renal insufficiencies” OR AB “chronic renal diseases” OR AB “chronic kidney failure” OR AB “chronic renal failure” OR DE "Hemodialysis" OR TI “renal dialysis” OR TI “kidney dialysis” OR TI hemodialysis OR TI haemodialysis OR AB “renal dialysis” OR AB “kidney dialysis” OR AB hemodialysis OR AB haemodialysis OR TI hyperlipidemia OR TI hyperlipidemias OR TI hyperlipidemic OR TI hyperlipemia OR TI hyperlipemias OR TI hyperlipemic OR TI lipidemia OR TI lipidemias OR TI lipidemic OR TI lipemia OR TI lipemias OR TI lipemic OR AB hyperlipidemia OR AB hyperlipidemias OR AB hyperlipidemic OR AB hyperlipemia OR

	AB hyperlipemias OR AB hyperlipemic OR AB lipidemia OR AB lipidemias OR AB lipidemic OR AB lipemia OR AB lipemias OR AB lipemic OR DE "Cholesterol" OR TI hypercholesterolemia OR TI hyper-cholesterolemia OR TI hypercholesterolemic OR TI hyper-cholesterolemic OR TI cholesterol OR TI hypertriglyceridemia OR TI hyper-triglyceridemia OR TI hypertriglyceridemic OR TI hyper-triglyceridemic OR AB hypercholesterolemia OR AB hyper-cholesterolemia OR AB hypercholesterolemic OR AB hyper-cholesterolemic OR AB cholesterol OR AB hypertriglyceridemia OR AB hyper-triglyceridemia OR AB hypertriglyceridemic OR AB hyper-triglyceridemic OR TI triglycerid OR TI triglycerids OR AB triglycerid OR AB triglycerids)
S5	(**DE "Asthma" OR TI asthma OR TI asthmatic OR AB asthma OR AB asthmatic**) AND (TI "heart failure" OR TI "heart failures" OR AB "heart failure" OR AB "heart failures" OR DE "Chronic Obstructive Pulmonary Disease" OR TI "chronic obstructive pulmonary disease" OR TI "chronic obstructive lung disease" OR TI "chronic obstructive pulmonary diseases" OR TI "chronic obstructive lung diseases" OR TI "COPD" OR AB "chronic obstructive pulmonary disease" OR AB "chronic obstructive lung disease" OR AB "chronic obstructive pulmonary diseases" OR AB "chronic obstructive lung diseases" OR AB "COPD" OR DE "Diabetes Mellitus" OR DE "Type 2 Diabetes" OR TI diabetes OR TI diabetic OR AB diabetes OR AB diabetic OR DE "Hypertension" OR TI hypertension OR AB hypertension OR DE "Coronary Heart Disease" OR TI "coronary artery disease" OR TI "coronary atherosclerosis" OR TI "coronary arteriosclerosis" OR TI "coronary artery diseases" OR AB "coronary artery disease" OR AB "coronary atherosclerosis" OR AB "coronary arteriosclerosis" OR AB "coronary artery diseases" OR DE "Arthritis" OR DE "Rheumatoid Arthritis" OR TI arthritis OR TI arthritic OR TI osteoarthritis OR TI osteoarthritic OR TI periarthritis OR TI periarthritic OR AB arthritis OR AB arthritic OR AB osteoarthritis OR AB osteoarthritic OR AB periarthritis OR AB periarthritic OR DE "Osteoporosis" OR TI osteoporosis OR TI osteoporotic OR AB osteoporosis OR AB osteoporotic OR DE "Neoplasms" OR DE "Breast Neoplasms" OR DE "Endocrine Neoplasms" OR DE "Leukemias" OR DE "Melanoma" OR DE "Metastasis" OR DE "Nervous System Neoplasms" OR TI neoplas* OR TI cancer* OR TI tumor* OR TI tumour* OR TI carcinoma* OR TI oncolog* OR AB neoplas* OR AB cancer* OR AB tumor* OR AB tumour* OR AB carcinoma* OR AB oncolog* OR TI "chronic renal insufficiency" OR TI "chronic kidney insufficiency" OR TI "chronic renal disease" OR TI "chronic kidney disease" OR TI "chronic renal insufficiencies" OR TI "chronic renal diseases" OR TI "chronic kidney failure" OR TI "chronic renal failure" OR AB "chronic renal insufficiency" OR AB "chronic kidney insufficiency" OR AB "chronic renal disease" OR AB "chronic kidney disease" OR AB "chronic renal insufficiencies" OR AB "chronic renal diseases" OR AB "chronic kidney failure" OR AB "chronic renal failure" OR DE "Hemodialysis" OR TI "renal dialysis" OR TI "kidney dialysis" OR TI hemodialysis OR TI haemodialysis OR AB "renal dialysis" OR AB "kidney dialysis" OR AB hemodialysis OR AB haemodialysis OR TI hyperlipidemia OR TI hyperlipidemias OR TI hyperlipidemic OR TI hyperlipemia OR TI hyperlipemias OR TI hyperlipemic OR TI lipidemia OR TI lipidemias OR TI lipidemic OR TI lipemia OR TI lipemias OR TI lipemic OR AB hyperlipidemia OR AB hyperlipidemias OR AB hyperlipidemic OR AB hyperlipemia OR AB hyperlipemias OR AB hyperlipemic OR AB lipidemia OR AB lipidemias OR AB lipidemic OR AB lipemia OR AB lipemias OR AB lipemic OR DE "Cholesterol" OR TI hypercholesterolemia OR TI hyper-cholesterolemia OR TI hypercholesterolemic OR TI hyper-cholesterolemic OR TI cholesterol OR TI hypertriglyceridemia OR TI hyper-triglyceridemia OR TI hypertriglyceridemic OR TI hyper-triglyceridemic OR AB hypercholesterolemia OR AB hyper-cholesterolemia OR AB hypercholesterolemic OR AB hyper-cholesterolemic OR AB cholesterol OR AB hypertriglyceridemia OR AB hyper-triglyceridemia OR AB hypertriglyceridemic OR AB

	hyper-triglyceridemic OR TI triglycerid OR TI triglycerids OR AB triglycerid OR AB triglycerids)
S6	(**DE "Diabetes Mellitus" OR DE "Type 2 Diabetes" OR TI diabetes OR TI diabetic OR AB diabetes OR AB diabetic**) AND (TI “heart failure” OR TI “heart failures” OR AB “heart failure” OR AB “heart failures” OR DE "Chronic Obstructive Pulmonary Disease" OR TI “chronic obstructive pulmonary disease” OR TI “chronic obstructive lung disease” OR TI “chronic obstructive pulmonary diseases” OR TI “chronic obstructive lung diseases” OR TI "COPD" OR AB “chronic obstructive pulmonary disease” OR AB “chronic obstructive lung disease” OR AB “chronic obstructive pulmonary diseases” OR AB “chronic obstructive lung diseases” OR AB "COPD" OR DE "Asthma" OR TI asthma OR TI asthmatic OR AB asthma OR AB asthmatic OR DE "Hypertension" OR TI hypertension OR AB hypertension OR DE "Coronary Heart Disease" OR TI “coronary artery disease” OR TI “coronary atherosclerosis” OR TI “coronary arteriosclerosis” OR TI “coronary artery diseases” OR AB “coronary artery disease” OR AB “coronary atherosclerosis” OR AB “coronary arteriosclerosis” OR AB “coronary artery diseases” OR DE "Arthritis" OR DE "Rheumatoid Arthritis" OR TI arthritis OR TI arthritic OR TI osteoarthritis OR TI osteoarthritic OR TI periarthritis OR TI periarthritic OR AB arthritis OR AB arthritic OR AB osteoarthritis OR AB osteoarthritic OR AB periarthritis OR AB periarthritic OR DE "Osteoporosis" OR TI osteoporosis OR TI osteoporotic OR AB osteoporosis OR AB osteoporotic OR DE "Neoplasms" OR DE "Breast Neoplasms" OR DE "Endocrine Neoplasms" OR DE "Leukemias" OR DE "Melanoma" OR DE "Metastasis" OR DE "Nervous System Neoplasms" OR TI neoplas* OR TI cancer* OR TI tumor* OR TI tumour* OR TI carcinoma* OR TI oncolog* OR AB neoplas* OR AB cancer* OR AB tumor* OR AB tumour* OR AB carcinoma* OR AB oncolog* OR TI “chronic renal insufficiency” OR TI “chronic kidney insufficiency” OR TI “chronic renal disease” OR TI “chronic kidney disease” OR TI “chronic renal insufficiencies” OR TI “chronic renal diseases” OR TI “chronic kidney failure” OR TI “chronic renal failure” OR AB “chronic renal insufficiency” OR AB “chronic kidney insufficiency” OR AB “chronic renal disease” OR AB “chronic kidney disease” OR AB “chronic renal insufficiencies” OR AB “chronic renal diseases” OR AB “chronic kidney failure” OR AB “chronic renal failure” OR DE "Hemodialysis" OR TI “renal dialysis” OR TI “kidney dialysis” OR TI hemodialysis OR TI haemodialysis OR AB “renal dialysis” OR AB “kidney dialysis” OR AB hemodialysis OR AB haemodialysis OR TI hyperlipidemia OR TI hyperlipidemias OR TI hyperlipidemic OR TI hyperlipemia OR TI hyperlipemias OR TI hyperlipemic OR TI lipidemia OR TI lipidemias OR TI lipidemic OR TI lipemia OR TI lipemias OR TI lipemic OR AB hyperlipidemia OR AB hyperlipidemias OR AB hyperlipidemic OR AB hyperlipemia OR AB hyperlipemias OR AB hyperlipemic OR AB lipidemia OR AB lipidemias OR AB lipidemic OR AB lipemia OR AB lipemias OR AB lipemic OR DE "Cholesterol" OR TI hypercholesterolemia OR TI hyper-cholesterolemia OR TI hypercholesterolemic OR TI hyper-cholesterolemic OR TI cholesterol OR TI hypertriglyceridemia OR TI hyper-triglyceridemia OR TI hypertriglyceridemic OR TI hyper-triglyceridemic OR AB hypercholesterolemia OR AB hyper-cholesterolemia OR AB hypercholesterolemic OR AB hyper-cholesterolemic OR AB cholesterol OR AB hypertriglyceridemia OR AB hyper-triglyceridemia OR AB hypertriglyceridemic OR AB hyper-triglyceridemic OR TI triglycerid OR TI triglycerids OR AB triglycerid OR AB triglycerids)
S7	(**DE "Hypertension" OR TI hypertension OR AB hypertension**) AND (TI “heart failure” OR TI “heart failures” OR AB “heart failure” OR AB “heart failures” OR DE "Chronic Obstructive Pulmonary Disease" OR TI “chronic obstructive pulmonary disease” OR TI “chronic obstructive lung disease” OR TI “chronic obstructive pulmonary diseases” OR TI “chronic obstructive lung diseases” OR TI "COPD" OR AB “chronic obstructive pulmonary disease” OR AB “chronic obstructive lung disease” OR AB “chronic obstructive pulmonary

	diseases” OR AB “chronic obstructive lung diseases” OR AB "COPD" OR DE "Asthma" OR TI asthma OR TI asthmatic OR AB asthma OR AB asthmatic OR DE "Diabetes Mellitus" OR DE "Type 2 Diabetes" OR TI diabetes OR TI diabetic OR AB diabetes OR AB diabetic OR DE "Coronary Heart Disease" OR TI “coronary artery disease” OR TI “coronary atherosclerosis” OR TI “coronary arteriosclerosis” OR TI “coronary artery diseases” OR AB “coronary artery disease” OR AB “coronary atherosclerosis” OR AB “coronary arteriosclerosis” OR AB “coronary artery diseases” OR DE "Arthritis" OR DE "Rheumatoid Arthritis" OR TI arthritis OR TI arthritic OR TI osteoarthritis OR TI osteoarthritic OR TI periarthritis OR TI periarthritic OR AB arthritis OR AB arthritic OR AB osteoarthritis OR AB osteoarthritic OR AB periarthritis OR AB periarthritic OR DE "Osteoporosis" OR TI osteoporosis OR TI osteoporotic OR AB osteoporosis OR AB osteoporotic OR DE "Neoplasms" OR DE "Breast Neoplasms" OR DE "Endocrine Neoplasms" OR DE "Leukemias" OR DE "Melanoma" OR DE "Metastasis" OR DE "Nervous System Neoplasms" OR TI neoplas* OR TI cancer* OR TI tumor* OR TI tumour* OR TI carcinoma* OR TI oncolog* OR AB neoplas* OR AB cancer* OR AB tumor* OR AB tumour* OR AB carcinoma* OR AB oncolog* OR TI “chronic renal insufficiency” OR TI “chronic kidney insufficiency” OR TI “chronic renal disease” OR TI “chronic kidney disease” OR TI “chronic renal insufficiencies” OR TI “chronic renal diseases” OR TI “chronic kidney failure” OR TI “chronic renal failure” OR AB “chronic renal insufficiency” OR AB “chronic kidney insufficiency” OR AB “chronic renal disease” OR AB “chronic kidney disease” OR AB “chronic renal insufficiencies” OR AB “chronic renal diseases” OR AB “chronic kidney failure” OR AB “chronic renal failure” OR DE "Hemodialysis" OR TI “renal dialysis” OR TI “kidney dialysis” OR TI hemodialysis OR TI haemodialysis OR AB “renal dialysis” OR AB “kidney dialysis” OR AB hemodialysis OR AB haemodialysis OR TI hyperlipidemia OR TI hyperlipidemias OR TI hyperlipidemic OR TI hyperlipemia OR TI hyperlipemias OR TI hyperlipemic OR TI lipidemia OR TI lipidemias OR TI lipidemic OR TI lipemia OR TI lipemias OR TI lipemic OR AB hyperlipidemia OR AB hyperlipidemias OR AB hyperlipidemic OR AB hyperlipemia OR AB hyperlipemias OR AB hyperlipemic OR AB lipidemia OR AB lipidemias OR AB lipidemic OR AB lipemia OR AB lipemias OR AB lipemic OR DE "Cholesterol" OR TI hypercholesterolemia OR TI hyper-cholesterolemia OR TI hypercholesterolemic OR TI hyper-cholesterolemic OR TI cholesterol OR TI hypertriglyceridemia OR TI hyper-triglyceridemia OR TI hypertriglyceridemic OR TI hyper-triglyceridemic OR AB hypercholesterolemia OR AB hyper-cholesterolemia OR AB hypercholesterolemic OR AB hyper-cholesterolemic OR AB cholesterol OR AB hypertriglyceridemia OR AB hyper-triglyceridemia OR AB hypertriglyceridemic OR AB hyper-triglyceridemic OR TI triglycerid OR TI triglycerids OR AB triglycerid OR AB triglycerids)
S8	(**DE "Coronary Heart Disease" OR TI “coronary artery disease” OR TI “coronary atherosclerosis” OR TI “coronary arteriosclerosis” OR TI “coronary artery diseases” OR AB “coronary artery disease” OR AB “coronary atherosclerosis” OR AB “coronary arteriosclerosis” OR AB “coronary artery diseases”**) AND (TI “heart failure” OR TI “heart failures” OR AB “heart failure” OR AB “heart failures” OR DE "Chronic Obstructive Pulmonary Disease" OR TI “chronic obstructive pulmonary disease” OR TI “chronic obstructive lung disease” OR TI “chronic obstructive pulmonary diseases” OR TI “chronic obstructive lung diseases” OR TI "COPD" OR AB “chronic obstructive pulmonary disease” OR AB “chronic obstructive lung disease” OR AB “chronic obstructive pulmonary diseases” OR AB “chronic obstructive lung diseases” OR AB "COPD" OR DE "Asthma" OR TI asthma OR TI asthmatic OR AB asthma OR AB asthmatic OR DE "Diabetes Mellitus" OR DE "Type 2 Diabetes" OR TI diabetes OR TI diabetic OR AB diabetes OR AB diabetic OR DE "Hypertension" OR TI hypertension OR AB hypertension OR DE "Arthritis" OR DE "Rheumatoid Arthritis" OR TI arthritis OR TI arthritic OR TI osteoarthritis OR TI osteoarthritic OR TI periarthritis OR TI periarthritic OR AB arthritis

	OR AB arthritic OR AB osteoarthritis OR AB osteoarthritic OR AB periarthritis OR AB periarthritic OR DE "Osteoporosis" OR TI osteoporosis OR TI osteoporotic OR AB osteoporosis OR AB osteoporotic OR DE "Neoplasms" OR DE "Breast Neoplasms" OR DE "Endocrine Neoplasms" OR DE "Leukemias" OR DE "Melanoma" OR DE "Metastasis" OR DE "Nervous System Neoplasms" OR TI neoplas* OR TI cancer* OR TI tumor* OR TI tumour* OR TI carcinoma* OR TI oncolog* OR AB neoplas* OR AB cancer* OR AB tumor* OR AB tumour* OR AB carcinoma* OR AB oncolog* OR TI "chronic renal insufficiency" OR TI "chronic kidney insufficiency" OR TI "chronic renal disease" OR TI "chronic kidney disease" OR TI "chronic renal insufficiencies" OR TI "chronic renal diseases" OR TI "chronic kidney failure" OR TI "chronic renal failure" OR AB "chronic renal insufficiency" OR AB "chronic kidney insufficiency" OR AB "chronic renal disease" OR AB "chronic kidney disease" OR AB "chronic renal insufficiencies" OR AB "chronic renal diseases" OR AB "chronic kidney failure" OR AB "chronic renal failure" OR DE "Hemodialysis" OR TI "renal dialysis" OR TI "kidney dialysis" OR TI hemodialysis OR TI haemodialysis OR AB "renal dialysis" OR AB "kidney dialysis" OR AB hemodialysis OR AB haemodialysis OR TI hyperlipidemia OR TI hyperlipidemias OR TI hyperlipidemic OR TI hyperlipemia OR TI hyperlipemias OR TI hyperlipemic OR TI lipidemia OR TI lipidemias OR TI lipidemic OR TI lipemia OR TI lipemias OR TI lipemic OR AB hyperlipidemia OR AB hyperlipidemias OR AB hyperlipidemic OR AB hyperlipemia OR AB hyperlipemias OR AB hyperlipemic OR AB lipidemia OR AB lipidemias OR AB lipidemic OR AB lipemia OR AB lipemias OR AB lipemic OR DE "Cholesterol" OR TI hypercholesterolemia OR TI hyper-cholesterolemia OR TI hypercholesterolemic OR TI hyper-cholesterolemic OR TI cholesterol OR TI hypertriglyceridemia OR TI hyper-triglyceridemia OR TI hypertriglyceridemic OR TI hyper-triglyceridemic OR AB hypercholesterolemia OR AB hyper-cholesterolemia OR AB hypercholesterolemic OR AB hyper-cholesterolemic OR AB cholesterol OR AB hypertriglyceridemia OR AB hyper-triglyceridemia OR AB hypertriglyceridemic OR AB hyper-triglyceridemic OR TI triglycerid OR TI triglycerids OR AB triglycerid OR AB triglycerids)
S9	(**DE "Arthritis" OR DE "Rheumatoid Arthritis" OR TI arthritis OR TI arthritic OR TI osteoarthritis OR TI osteoarthritic OR TI periarthritis OR TI periarthritic OR AB arthritis OR AB arthritic OR AB osteoarthritis OR AB osteoarthritic OR AB periarthritis OR AB periarthritic**) AND (TI "heart failure" OR TI "heart failures" OR AB "heart failure" OR AB "heart failures" OR DE "Chronic Obstructive Pulmonary Disease" OR TI "chronic obstructive pulmonary disease" OR TI "chronic obstructive lung disease" OR TI "chronic obstructive pulmonary diseases" OR TI "chronic obstructive lung diseases" OR TI "COPD" OR AB "chronic obstructive pulmonary disease" OR AB "chronic obstructive lung disease" OR AB "chronic obstructive pulmonary diseases" OR AB "chronic obstructive lung diseases" OR AB "COPD" OR DE "Asthma" OR TI asthma OR TI asthmatic OR AB asthma OR AB asthmatic OR DE "Diabetes Mellitus" OR DE "Type 2 Diabetes" OR TI diabetes OR TI diabetic OR AB diabetes OR AB diabetic OR DE "Hypertension" OR TI hypertension OR AB hypertension OR DE "Coronary Heart Disease" OR TI "coronary artery disease" OR TI "coronary atherosclerosis" OR TI "coronary arteriosclerosis" OR TI "coronary artery diseases" OR AB "coronary artery disease" OR AB "coronary atherosclerosis" OR AB "coronary arteriosclerosis" OR AB "coronary artery diseases" OR DE "Osteoporosis" OR TI osteoporosis OR TI osteoporotic OR AB osteoporosis OR AB osteoporotic OR DE "Neoplasms" OR DE "Breast Neoplasms" OR DE "Endocrine Neoplasms" OR DE "Leukemias" OR DE "Melanoma" OR DE "Metastasis" OR DE "Nervous System Neoplasms" OR TI neoplas* OR TI cancer* OR TI tumor* OR TI tumour* OR TI carcinoma* OR TI oncolog* OR AB neoplas* OR AB cancer* OR AB tumor* OR AB tumour* OR AB carcinoma* OR AB oncolog* OR TI "chronic renal insufficiency" OR TI "chronic kidney insufficiency" OR TI "chronic renal disease" OR TI "chronic kidney disease" OR TI "chronic renal insufficiencies" OR TI "chronic renal

	diseases" OR TI "chronic kidney failure" OR TI "chronic renal failure" OR AB "chronic renal insufficiency" OR AB "chronic kidney insufficiency" OR AB "chronic renal disease" OR AB "chronic kidney disease" OR AB "chronic renal insufficiencies" OR AB "chronic renal diseases" OR AB "chronic kidney failure" OR AB "chronic renal failure" OR DE "Hemodialysis" OR TI "renal dialysis" OR TI "kidney dialysis" OR TI hemodialysis OR TI haemodialysis OR AB "renal dialysis" OR AB "kidney dialysis" OR AB hemodialysis OR AB haemodialysis OR TI hyperlipidemia OR TI hyperlipidemias OR TI hyperlipidemic OR TI hyperlipemia OR TI hyperlipemias OR TI hyperlipemic OR TI lipidemia OR TI lipidemias OR TI lipidemic OR TI lipemia OR TI lipemias OR TI lipemic OR AB hyperlipidemia OR AB hyperlipidemias OR AB hyperlipidemic OR AB hyperlipemia OR AB hyperlipemias OR AB hyperlipemic OR AB lipidemia OR AB lipidemias OR AB lipidemic OR AB lipemia OR AB lipemias OR AB lipemic OR DE "Cholesterol" OR TI hypercholesterolemia OR TI hyper-cholesterolemia OR TI hypercholesterolemic OR TI hyper-cholesterolemic OR TI cholesterol OR TI hypertriglyceridemia OR TI hyper-triglyceridemia OR TI hypertriglyceridemic OR TI hyper-triglyceridemic OR AB hypercholesterolemia OR AB hyper-cholesterolemia OR AB hypercholesterolemic OR AB hyper-cholesterolemic OR AB cholesterol OR AB hypertriglyceridemia OR AB hyper-triglyceridemia OR AB hypertriglyceridemic OR AB hyper-triglyceridemic OR TI triglycerid OR TI triglycerids OR AB triglycerid OR AB triglycerids)
S10	(**DE "Osteoporosis" OR TI osteoporosis OR TI osteoporotic OR AB osteoporosis OR AB osteoporotic**) AND (TI "heart failure" OR TI "heart failures" OR AB "heart failure" OR AB "heart failures" OR DE "Chronic Obstructive Pulmonary Disease" OR TI "chronic obstructive pulmonary disease" OR TI "chronic obstructive lung disease" OR TI "chronic obstructive pulmonary diseases" OR TI "chronic obstructive lung diseases" OR TI "COPD" OR AB "chronic obstructive pulmonary disease" OR AB "chronic obstructive lung disease" OR AB "chronic obstructive pulmonary diseases" OR AB "chronic obstructive lung diseases" OR AB "COPD" OR DE "Asthma" OR TI asthma OR TI asthmatic OR AB asthma OR AB asthmatic OR DE "Diabetes Mellitus" OR DE "Type 2 Diabetes" OR TI diabetes OR TI diabetic OR AB diabetes OR AB diabetic OR DE "Hypertension" OR TI hypertension OR AB hypertension OR DE "Coronary Heart Disease" OR TI "coronary artery disease" OR TI "coronary atherosclerosis" OR TI "coronary arteriosclerosis" OR TI "coronary artery diseases" OR AB "coronary artery disease" OR AB "coronary atherosclerosis" OR AB "coronary arteriosclerosis" OR AB "coronary artery diseases" OR DE "Arthritis" OR DE "Rheumatoid Arthritis" OR TI arthritis OR TI arthritic OR TI osteoarthritis OR TI osteoarthritic OR TI periarthritis OR TI periarthritic OR AB arthritis OR AB arthritic OR AB osteoarthritis OR AB osteoarthritic OR AB periarthritis OR AB periarthritic OR DE "Neoplasms" OR DE "Breast Neoplasms" OR DE "Endocrine Neoplasms" OR DE "Leukemias" OR DE "Melanoma" OR DE "Metastasis" OR DE "Nervous System Neoplasms" OR TI neoplas* OR TI cancer* OR TI tumor* OR TI tumour* OR TI carcinoma* OR TI oncolog* OR AB neoplas* OR AB cancer* OR AB tumor* OR AB tumour* OR AB carcinoma* OR AB oncolog* OR TI "chronic renal insufficiency" OR TI "chronic kidney insufficiency" OR TI "chronic renal disease" OR TI "chronic kidney disease" OR TI "chronic renal insufficiencies" OR TI "chronic renal diseases" OR TI "chronic kidney failure" OR TI "chronic renal failure" OR AB "chronic renal insufficiency" OR AB "chronic kidney insufficiency" OR AB "chronic renal disease" OR AB "chronic kidney disease" OR AB "chronic renal insufficiencies" OR AB "chronic renal diseases" OR AB "chronic kidney failure" OR AB "chronic renal failure" OR DE "Hemodialysis" OR TI "renal dialysis" OR TI "kidney dialysis" OR TI hemodialysis OR TI haemodialysis OR AB "renal dialysis" OR AB "kidney dialysis" OR AB hemodialysis OR AB haemodialysis OR TI hyperlipidemia OR TI hyperlipidemias OR TI hyperlipidemic OR TI hyperlipemia OR TI hyperlipemias OR TI hyperlipemic OR TI lipidemia OR TI lipidemias OR TI lipidemic OR TI lipemia OR TI lipemias OR TI lipemic OR AB hyperlipidemia OR AB hyperlipidemias

	OR AB hyperlipidemic OR AB hyperlipemia OR AB hyperlipemias OR AB hyperlipemic OR AB lipidemia OR AB lipidemias OR AB lipidemic OR AB lipemia OR AB lipemias OR AB lipemic OR DE "Cholesterol" OR TI hypercholesterolemia OR TI hyper-cholesterolemia OR TI hypercholesterolemic OR TI hyper-cholesterolemic OR TI cholesterol OR TI hypertriglyceridemia OR TI hyper-triglyceridemia OR TI hypertriglyceridemic OR TI hyper-triglyceridemic OR AB hypercholesterolemia OR AB hyper-cholesterolemia OR AB hypercholesterolemic OR AB hyper-cholesterolemic OR AB cholesterol OR AB hypertriglyceridemia OR AB hyper-triglyceridemia OR AB hypertriglyceridemic OR AB hyper-triglyceridemic OR TI triglycerid OR TI triglycerids OR AB triglycerid OR AB triglycerids)
S11	(**DE "Neoplasms" OR DE "Breast Neoplasms" OR DE "Endocrine Neoplasms" OR DE "Leukemias" OR DE "Melanoma" OR DE "Metastasis" OR DE "Nervous System Neoplasms" OR TI neoplas* OR TI cancer* OR TI tumor* OR TI tumour* OR TI carcinoma* OR TI oncolog* OR AB neoplas* OR AB cancer* OR AB tumor* OR AB tumour* OR AB carcinoma* OR AB oncolog***) AND (TI “heart failure” OR TI “heart failures” OR AB “heart failure” OR AB “heart failures” OR DE "Chronic Obstructive Pulmonary Disease" OR TI “chronic obstructive pulmonary disease” OR TI “chronic obstructive lung disease” OR TI “chronic obstructive pulmonary diseases” OR TI “chronic obstructive lung diseases” OR TI "COPD" OR AB “chronic obstructive pulmonary disease” OR AB “chronic obstructive lung disease” OR AB “chronic obstructive pulmonary diseases” OR AB “chronic obstructive lung diseases” OR AB "COPD" OR DE "Asthma" OR TI asthma OR TI asthmatic OR AB asthma OR AB asthmatic OR DE "Diabetes Mellitus" OR DE "Type 2 Diabetes" OR TI diabetes OR TI diabetic OR AB diabetes OR AB diabetic OR DE "Hypertension" OR TI hypertension OR AB hypertension OR DE "Coronary Heart Disease" OR TI “coronary artery disease” OR TI “coronary atherosclerosis” OR TI “coronary arteriosclerosis” OR TI “coronary artery diseases” OR AB “coronary artery disease” OR AB “coronary atherosclerosis” OR AB “coronary arteriosclerosis” OR AB “coronary artery diseases” OR DE "Arthritis" OR DE "Rheumatoid Arthritis" OR TI arthritis OR TI arthritic OR TI osteoarthritis OR TI osteoarthritic OR TI periarthritis OR TI periarthritic OR AB arthritis OR AB arthritic OR AB osteoarthritis OR AB osteoarthritic OR AB periarthritis OR AB periarthritic OR DE "Osteoporosis" OR TI osteoporosis OR TI osteoporotic OR AB osteoporosis OR AB osteoporotic OR TI “chronic renal insufficiency” OR TI “chronic kidney insufficiency” OR TI “chronic renal disease” OR TI “chronic kidney disease” OR TI “chronic renal insufficiencies” OR TI “chronic renal diseases” OR TI “chronic kidney failure” OR TI “chronic renal failure” OR AB “chronic renal insufficiency” OR AB “chronic kidney insufficiency” OR AB “chronic renal disease” OR AB “chronic kidney disease” OR AB “chronic renal insufficiencies” OR AB “chronic renal diseases” OR AB “chronic kidney failure” OR AB “chronic renal failure” OR DE "Hemodialysis" OR TI “renal dialysis” OR TI “kidney dialysis” OR TI hemodialysis OR TI haemodialysis OR AB “renal dialysis” OR AB “kidney dialysis” OR AB hemodialysis OR AB haemodialysis OR TI hyperlipidemia OR TI hyperlipidemias OR TI hyperlipidemic OR TI hyperlipemia OR TI hyperlipemias OR TI hyperlipemic OR TI lipidemia OR TI lipidemias OR TI lipidemic OR TI lipemia OR TI lipemias OR TI lipemic OR AB hyperlipidemia OR AB hyperlipidemias OR AB hyperlipidemic OR AB hyperlipemia OR AB hyperlipemias OR AB hyperlipemic OR AB lipidemia OR AB lipidemias OR AB lipidemic OR AB lipemia OR AB lipemias OR AB lipemic OR DE "Cholesterol" OR TI hypercholesterolemia OR TI hyper-cholesterolemia OR TI hypercholesterolemic OR TI hyper-cholesterolemic OR TI cholesterol OR TI hypertriglyceridemia OR TI hyper-triglyceridemia OR TI hypertriglyceridemic OR TI hyper-triglyceridemic OR AB hypercholesterolemia OR AB hyper-cholesterolemia OR AB hypercholesterolemic OR AB hyper-cholesterolemic OR AB cholesterol OR AB hypertriglyceridemia OR AB hyper-triglyceridemia OR AB hypertriglyceridemic OR AB

	hyper-triglyceridemic OR TI triglycerid OR TI triglycerids OR AB triglycerid OR AB triglycerids)
S12	(**TI “chronic renal insufficiency” OR TI “chronic kidney insufficiency” OR TI “chronic renal disease” OR TI “chronic kidney disease” OR TI “chronic renal insufficiencies” OR TI “chronic renal diseases” OR TI “chronic kidney failure” OR TI “chronic renal failure” OR AB “chronic renal insufficiency” OR AB “chronic kidney insufficiency” OR AB “chronic renal disease” OR AB “chronic kidney disease” OR AB “chronic renal insufficiencies” OR AB “chronic renal diseases” OR AB “chronic kidney failure” OR AB “chronic renal failure” OR DE "Hemodialysis" OR TI “renal dialysis” OR TI “kidney dialysis” OR TI hemodialysis OR TI haemodialysis OR AB “renal dialysis” OR AB “kidney dialysis” OR AB hemodialysis OR AB haemodialysis**) AND (TI “heart failure” OR TI “heart failures” OR AB “heart failure” OR AB “heart failures” OR DE "Chronic Obstructive Pulmonary Disease" OR TI “chronic obstructive pulmonary disease” OR TI “chronic obstructive lung disease” OR TI “chronic obstructive pulmonary diseases” OR TI “chronic obstructive lung diseases” OR TI "COPD" OR AB “chronic obstructive pulmonary disease” OR AB “chronic obstructive lung disease” OR AB “chronic obstructive pulmonary diseases” OR AB “chronic obstructive lung diseases” OR AB "COPD" OR DE "Asthma" OR TI asthma OR TI asthmatic OR AB asthma OR AB asthmatic OR DE "Diabetes Mellitus" OR DE "Type 2 Diabetes" OR TI diabetes OR TI diabetic OR AB diabetes OR AB diabetic OR DE "Hypertension" OR TI hypertension OR AB hypertension OR DE "Coronary Heart Disease" OR TI “coronary artery disease” OR TI “coronary atherosclerosis” OR TI “coronary arteriosclerosis” OR TI “coronary artery diseases” OR AB “coronary artery disease” OR AB “coronary atherosclerosis” OR AB “coronary arteriosclerosis” OR AB “coronary artery diseases” OR DE "Arthritis" OR DE "Rheumatoid Arthritis" OR TI arthritis OR TI arthritic OR TI osteoarthritis OR TI osteoarthritic OR TI periarthritis OR TI periarthritic OR AB arthritis OR AB arthritic OR AB osteoarthritis OR AB osteoarthritic OR AB periarthritis OR AB periarthritic OR DE "Osteoporosis" OR TI osteoporosis OR TI osteoporotic OR AB osteoporosis OR AB osteoporotic OR DE "Neoplasms" OR DE "Breast Neoplasms" OR DE "Endocrine Neoplasms" OR DE "Leukemias" OR DE "Melanoma" OR DE "Metastasis" OR DE "Nervous System Neoplasms" OR TI neoplas* OR TI cancer* OR TI tumor* OR TI tumour* OR TI carcinoma* OR TI oncolog* OR AB neoplas* OR AB cancer* OR AB tumor* OR AB tumour* OR AB carcinoma* OR AB oncolog* OR TI hyperlipidemia OR TI hyperlipidemias OR TI hyperlipidemic OR TI hyperlipemia OR TI hyperlipemias OR TI hyperlipemic OR TI lipidemia OR TI lipidemias OR TI lipidemic OR TI lipemia OR TI lipemias OR TI lipemic OR AB hyperlipidemia OR AB hyperlipidemias OR AB hyperlipidemic OR AB hyperlipemia OR AB hyperlipemias OR AB hyperlipemic OR AB lipidemia OR AB lipidemias OR AB lipidemic OR AB lipemia OR AB lipemias OR AB lipemic OR DE "Cholesterol" OR TI hypercholesterolemia OR TI hyper-cholesterolemia OR TI hypercholesterolemic OR TI hyper-cholesterolemic OR TI cholesterol OR TI hypertriglyceridemia OR TI hyper-triglyceridemia OR TI hypertriglyceridemic OR TI hyper-triglyceridemic OR AB hypercholesterolemia OR AB hyper-cholesterolemia OR AB hypercholesterolemic OR AB hyper-cholesterolemic OR AB cholesterol OR AB hypertriglyceridemia OR AB hyper-triglyceridemia OR AB hypertriglyceridemic OR AB hyper-triglyceridemic OR TI triglycerid OR TI triglycerids OR AB triglycerid OR AB triglycerids)
S13	(**TI hyperlipidemia OR TI hyperlipidemias OR TI hyperlipidemic OR TI hyperlipemia OR TI hyperlipemias OR TI hyperlipemic OR TI lipidemia OR TI lipidemias OR TI lipidemic OR TI lipemia OR TI lipemias OR TI lipemic OR AB hyperlipidemia OR AB hyperlipidemias OR AB hyperlipidemic OR AB hyperlipemia OR AB hyperlipemias OR AB hyperlipemic OR AB lipidemia OR AB lipidemias OR AB lipidemic OR AB**

	**lipemia OR AB lipemias OR AB lipemic OR DE "Cholesterol" OR TI hypercholesterolemia OR TI hyper-cholesterolemia OR TI hypercholesterolemic OR TI hyper-cholesterolemic OR TI cholesterol OR TI hypertriglyceridemia OR TI hyper-triglyceridemia OR TI hypertriglyceridemic OR TI hyper-triglyceridemic OR AB hypercholesterolemia OR AB hyper-cholesterolemia OR AB hypercholesterolemic OR AB hyper-cholesterolemic OR AB cholesterol OR AB hypertriglyceridemia OR AB hyper-triglyceridemia OR AB hypertriglyceridemic OR AB hyper-triglyceridemic OR TI triglycerid OR TI triglycerids OR AB triglycerid OR AB triglycerids**) AND (TI "heart failure" OR TI "heart failures" OR AB "heart failure" OR AB "heart failures" OR DE "Chronic Obstructive Pulmonary Disease" OR TI "chronic obstructive pulmonary disease" OR TI "chronic obstructive lung disease" OR TI "chronic obstructive pulmonary diseases" OR TI "chronic obstructive lung diseases" OR TI "COPD" OR AB "chronic obstructive pulmonary disease" OR AB "chronic obstructive lung disease" OR AB "chronic obstructive pulmonary diseases" OR AB "chronic obstructive lung diseases" OR AB "COPD" OR DE "Asthma" OR TI asthma OR TI asthmatic OR AB asthma OR AB asthmatic OR DE "Diabetes Mellitus" OR DE "Type 2 Diabetes" OR TI diabetes OR TI diabetic OR AB diabetes OR AB diabetic OR DE "Hypertension" OR TI hypertension OR AB hypertension OR DE "Coronary Heart Disease" OR TI "coronary artery disease" OR TI "coronary atherosclerosis" OR TI "coronary arteriosclerosis" OR TI "coronary artery diseases" OR AB "coronary artery disease" OR AB "coronary atherosclerosis" OR AB "coronary arteriosclerosis" OR AB "coronary artery diseases" OR DE "Arthritis" OR DE "Rheumatoid Arthritis" OR TI arthritis OR TI arthritic OR TI osteoarthritis OR TI osteoarthritic OR TI periarthritis OR TI periarthritic OR AB arthritis OR AB arthritic OR AB osteoarthritis OR AB osteoarthritic OR AB periarthritis OR AB periarthriticOR DE "Osteoporosis" OR TI osteoporosis OR TI osteoporotic OR AB osteoporosis OR AB osteoporotic OR DE "Neoplasms" OR DE "Breast Neoplasms" OR DE "Endocrine Neoplasms" OR DE "Leukemias" OR DE "Melanoma" OR DE "Metastasis" OR DE "Nervous System Neoplasms" OR TI neoplas* OR TI cancer* OR TI tumor* OR TI tumour* OR TI carcinoma* OR TI oncolog* OR AB neoplas* OR AB cancer* OR AB tumor* OR AB tumour* OR AB carcinoma* OR AB oncolog* OR TI "chronic renal insufficiency" OR TI "chronic kidney insufficiency" OR TI "chronic renal disease" OR TI "chronic kidney disease" OR TI "chronic renal insufficiencies" OR TI "chronic renal diseases" OR TI "chronic kidney failure" OR TI "chronic renal failure" OR AB "chronic renal insufficiency" OR AB "chronic kidney insufficiency" OR AB "chronic renal disease" OR AB "chronic kidney disease" OR AB "chronic renal insufficiencies" OR AB "chronic renal diseases" OR AB "chronic kidney failure" OR AB "chronic renal failure" OR DE "Hemodialysis" OR TI "renal dialysis" OR TI "kidney dialysis" OR TI hemodialysis OR TI haemodialysis OR AB "renal dialysis" OR AB "kidney dialysis" OR AB hemodialysis OR AB haemodialysis)
**S14**	**S2 OR S3 OR S4 OR S5 OR S6 OR S7 OR S8 OR S9 OR S10 OR S11 OR S12 OR S13**
S15	ZG "aged (65 yrs & older)" OR ZG "very old (85 yrs & older)" OR DE "Geriatric Patients" OR TI aging OR TI older OR TI elder* OR TI geriatr* OR AB aging OR AB older OR AB elder* OR AB geriatr*
**S16**	**S1 AND S14 AND S15**

Scopus

#1	TITLE-ABS-KEY("motivational interview*" OR "motivational technique*" OR "motivational counsel*" OR "motivational enhancement therap*" OR "motivational therap*" OR "motivational intervention*" OR "motivational strateg*" OR "motivational approach*" OR "motivational communicat*" OR "motivational language*")
#2	TITLE-ABS-KEY(polypharma* OR polymedicat* OR poly-pharma* OR poly-medicat* OR multimedicat* OR multi-medicat* OR plurimedicat* OR polypatholog* OR poly-patholog* OR pluripatholog* OR pluri-patholog* OR multipatholog* OR multi-patholog* OR comorbid* OR multimorbid* OR multi-morbid* OR plurimorbid* OR polymorbid* OR poly-morbid* OR concurrent* OR concomitant* OR coexist* OR co-exist*)
#3	TITLE-ABS-KEY(diseases OR conditions OR disorders OR illnesses OR "health problems" AND multiple)
#4	TITLE-ABS-KEY(**("heart failure" OR "heart failures")** AND ("chronic obstructive pulmonary disease" OR "chronic obstructive lung disease" OR "chronic obstructive pulmonary diseases" OR "chronic obstructive lung diseases" OR COPD OR asthma OR asthmatic OR diabetes OR diabetic OR hypertension OR "coronary artery disease" OR "coronary atherosclerosis" OR "coronary arteriosclerosis" OR "coronary artery diseases" OR arthritis OR arthritic OR osteoarthritis OR osteoarthritic OR periarthritis OR periarthritic OR osteoporosis OR osteoporotic OR neoplas* OR cancer* OR tumor* OR tumour* OR carcinoma* OR oncolog* OR "chronic renal insufficiency" OR "chronic kidney insufficiency" OR "chronic renal disease" OR "chronic kidney disease" OR "chronic renal insufficiencies" OR "chronic renal diseases" OR "chronic kidney failure" OR "chronic renal failure" OR "renal dialysis" OR "kidney dialysis" OR hemodialysis OR haemodialysis OR hyperlipidemia OR hyperlipidemias OR hyperlipidemic OR hyperlipemia OR hyperlipemias OR hyperlipemic OR lipidemia OR lipidemias OR lipidemic OR lipemia OR lipemias OR lipemic OR hypercholesterolemia OR hyper-cholesterolemia OR hypercholesterolemic OR hyper-cholesterolemic OR cholesterol OR hypertriglyceridemia OR hyper-triglyceridemia OR hypertriglyceridemic OR hyper-triglyceridemic OR triglycerid OR triglycerids))
#5	TITLE-ABS-KEY((**"chronic obstructive pulmonary disease" OR "chronic obstructive lung disease" OR "chronic obstructive pulmonary diseases" OR "chronic obstructive lung diseases" OR COPD**) AND ("heart failure" OR "heart failures" OR asthma OR asthmatic OR diabetes OR diabetic OR hypertension OR "coronary artery disease" OR "coronary atherosclerosis" OR "coronary arteriosclerosis" OR "coronary artery diseases" OR arthritis OR arthritic OR osteoarthritis OR osteoarthritic OR periarthritis OR periarthritic OR osteoporosis OR osteoporotic OR neoplas* OR cancer* OR tumor* OR tumour* OR carcinoma* OR oncolog* OR "chronic renal insufficiency" OR "chronic kidney insufficiency" OR "chronic renal disease" OR "chronic kidney disease" OR "chronic renal insufficiencies" OR "chronic renal diseases" OR "chronic kidney failure" OR "chronic renal failure" OR "renal dialysis" OR "kidney dialysis" OR hemodialysis OR haemodialysis OR hyperlipidemia OR hyperlipidemias OR hyperlipidemic OR hyperlipemia OR hyperlipemias OR hyperlipemic OR lipidemia OR lipidemias OR lipidemic OR lipemia OR lipemias OR lipemic OR hypercholesterolemia OR hyper-cholesterolemia OR hypercholesterolemic OR hyper-cholesterolemic OR cholesterol OR hypertriglyceridemia OR hyper-triglyceridemia OR hypertriglyceridemic OR hyper-triglyceridemic OR triglycerid OR triglycerids))
#6	TITLE-ABS-KEY((**asthma OR asthmatic**) AND ("heart failure" OR "heart failures" OR "chronic obstructive pulmonary disease" OR "chronic obstructive lung disease" OR "chronic obstructive pulmonary diseases" OR "chronic obstructive lung diseases" OR COPD OR diabetes OR diabetic OR hypertension OR "coronary artery disease" OR "coronary atherosclerosis" OR "coronary arteriosclerosis" OR "coronary artery diseases" OR arthritis

	OR arthritic OR osteoarthritis OR osteoarthritic OR periarthritis OR periarthritic OR osteoporosis OR osteoporotic OR neoplas* OR cancer* OR tumor* OR tumour* OR carcinoma* OR oncolog* OR “chronic renal insufficiency” OR “chronic kidney insufficiency” OR “chronic renal disease” OR “chronic kidney disease” OR “chronic renal insufficiencies” OR “chronic renal diseases” OR “chronic kidney failure” OR “chronic renal failure” OR “renal dialysis” OR “kidney dialysis” OR hemodialysis OR haemodialysis OR hyperlipidemia OR hyperlipidemias OR hyperlipidemic OR hyperlipemia OR hyperlipemias OR hyperlipemic OR lipidemia OR lipidemias OR lipidemic OR lipemia OR lipemias OR lipemic OR hypercholesterolemia OR hyper-cholesterolemia OR hypercholesterolemic OR hyper-cholesterolemic OR cholesterol OR hypertriglyceridemia OR hyper-triglyceridemia OR hypertriglyceridemic OR hyper-triglyceridemic OR triglycerid OR triglycerids))
#7	TITLE-ABS-KEY((**diabetes OR diabetic**) AND (“heart failure” OR “heart failures” OR “chronic obstructive pulmonary disease” OR “chronic obstructive lung disease” OR “chronic obstructive pulmonary diseases” OR “chronic obstructive lung diseases” OR COPD OR asthma OR asthmatic OR hypertension OR “coronary artery disease” OR “coronary atherosclerosis” OR “coronary arteriosclerosis” OR “coronary artery diseases” OR arthritis OR arthritic OR osteoarthritis OR osteoarthritic OR periarthritis OR periarthritic OR osteoporosis OR osteoporotic OR neoplas* OR cancer* OR tumor* OR tumour* OR carcinoma* OR oncolog* OR “chronic renal insufficiency” OR “chronic kidney insufficiency” OR “chronic renal disease” OR “chronic kidney disease” OR “chronic renal insufficiencies” OR “chronic renal diseases” OR “chronic kidney failure” OR “chronic renal failure” OR “renal dialysis” OR “kidney dialysis” OR hemodialysis OR haemodialysis OR hyperlipidemia OR hyperlipidemias OR hyperlipidemic OR hyperlipemia OR hyperlipemias OR hyperlipemic OR lipidemia OR lipidemias OR lipidemic OR lipemia OR lipemias OR lipemic OR hypercholesterolemia OR hyper-cholesterolemia OR hypercholesterolemic OR hyper-cholesterolemic OR cholesterol OR hypertriglyceridemia OR hyper-triglyceridemia OR hypertriglyceridemic OR hyper-triglyceridemic OR triglycerid OR triglycerids))
#8	TITLE-ABS-KEY(**hypertension** AND (“heart failure” OR “heart failures” OR “chronic obstructive pulmonary disease” OR “chronic obstructive lung disease” OR “chronic obstructive pulmonary diseases” OR “chronic obstructive lung diseases” OR COPD OR asthma OR asthmatic OR diabetes OR diabetic OR “coronary artery disease” OR “coronary atherosclerosis” OR “coronary arteriosclerosis” OR “coronary artery diseases” OR arthritis OR arthritic OR osteoarthritis OR osteoarthritic OR periarthritis OR periarthritic OR osteoporosis OR osteoporotic OR neoplas* OR cancer* OR tumor* OR tumour* OR carcinoma* OR oncolog* OR “chronic renal insufficiency” OR “chronic kidney insufficiency” OR “chronic renal disease” OR “chronic kidney disease” OR “chronic renal insufficiencies” OR “chronic renal diseases” OR “chronic kidney failure” OR “chronic renal failure” OR “renal dialysis” OR “kidney dialysis” OR hemodialysis OR haemodialysis OR hyperlipidemia OR hyperlipidemias OR hyperlipidemic OR hyperlipemia OR hyperlipemias OR hyperlipemic OR lipidemia OR lipidemias OR lipidemic OR lipemia OR lipemias OR lipemic OR hypercholesterolemia OR hyper-cholesterolemia OR hypercholesterolemic OR hyper-cholesterolemic OR cholesterol OR hypertriglyceridemia OR hyper-triglyceridemia OR hypertriglyceridemic OR hyper-triglyceridemic OR triglycerid OR triglycerids))
#9	TITLE-ABS-KEY((**“coronary artery disease” OR “coronary atherosclerosis” OR “coronary arteriosclerosis” OR “coronary artery diseases”**) AND (“heart failure” OR “heart failures” OR “chronic obstructive pulmonary disease” OR “chronic obstructive lung disease” OR “chronic obstructive pulmonary diseases” OR “chronic obstructive lung diseases” OR COPD OR asthma OR asthmatic OR diabetes OR diabetic OR hypertension OR arthritis OR arthritic OR osteoarthritis OR osteoarthritic OR periarthritis OR periarthritic OR osteoporosis OR osteoporotic OR neoplas* OR cancer* OR tumor* OR tumour* OR carcinoma* OR oncolog* OR “chronic renal insufficiency” OR “chronic kidney

	insufficiency" OR "chronic renal disease" OR "chronic kidney disease" OR "chronic renal insufficiencies" OR "chronic renal diseases" OR "chronic kidney failure" OR "chronic renal failure" OR "renal dialysis" OR "kidney dialysis" OR hemodialysis OR haemodialysis OR hyperlipidemia OR hyperlipidemias OR hyperlipidemic OR hyperlipemia OR hyperlipemias OR hyperlipemic OR lipidemia OR lipidemias OR lipidemic OR lipemia OR lipemias OR lipemic OR hypercholesterolemia OR hyper-cholesterolemia OR hypercholesterolemic OR hyper-cholesterolemic OR cholesterol OR hypertriglyceridemia OR hyper-triglyceridemia OR hypertriglyceridemic OR hyper-triglyceridemic OR triglycerid OR triglycerids))
#10	TITLE-ABS-KEY((**arthritis OR arthritic OR osteoarthritis OR osteoarthritic OR periarthritis OR periarthritic**) AND ("heart failure" OR "heart failures" OR "chronic obstructive pulmonary disease" OR "chronic obstructive lung disease" OR "chronic obstructive pulmonary diseases" OR "chronic obstructive lung diseases" OR COPD OR asthma OR asthmatic OR diabetes OR diabetic OR hypertension OR "coronary artery disease" OR "coronary atherosclerosis" OR "coronary arteriosclerosis" OR "coronary artery diseases" OR osteoporosis OR osteoporotic OR neoplas* OR cancer* OR tumor* OR tumour* OR carcinoma* OR oncolog* OR "chronic renal insufficiency" OR "chronic kidney insufficiency" OR "chronic renal disease" OR "chronic kidney disease" OR "chronic renal insufficiencies" OR "chronic renal diseases" OR "chronic kidney failure" OR "chronic renal failure" OR "renal dialysis" OR "kidney dialysis" OR hemodialysis OR haemodialysis OR hyperlipidemia OR hyperlipidemias OR hyperlipidemic OR hyperlipemia OR hyperlipemias OR hyperlipemic OR lipidemia OR lipidemias OR lipidemic OR lipemia OR lipemias OR lipemic OR hypercholesterolemia OR hyper-cholesterolemia OR hypercholesterolemic OR hyper-cholesterolemic OR cholesterol OR hypertriglyceridemia OR hyper-triglyceridemia OR hypertriglyceridemic OR hyper-triglyceridemic OR triglycerid OR triglycerids))
#11	TITLE-ABS-KEY((**osteoporosis OR osteoporotic**) AND ("heart failure" OR "heart failures" OR "chronic obstructive pulmonary disease" OR "chronic obstructive lung disease" OR "chronic obstructive pulmonary diseases" OR "chronic obstructive lung diseases" OR COPD OR asthma OR asthmatic OR diabetes OR diabetic OR hypertension OR "coronary artery disease" OR "coronary atherosclerosis" OR "coronary arteriosclerosis" OR "coronary artery diseases" OR arthritis OR arthritic OR osteoarthritis OR osteoarthritic OR periarthritis OR periarthritic OR neoplas* OR cancer* OR tumor* OR tumour* OR carcinoma* OR oncolog* OR "chronic renal insufficiency" OR "chronic kidney insufficiency" OR "chronic renal disease" OR "chronic kidney disease" OR "chronic renal insufficiencies" OR "chronic renal diseases" OR "chronic kidney failure" OR "chronic renal failure" OR "renal dialysis" OR "kidney dialysis" OR hemodialysis OR haemodialysis OR hyperlipidemia OR hyperlipidemias OR hyperlipidemic OR hyperlipemia OR hyperlipemias OR hyperlipemic OR lipidemia OR lipidemias OR lipidemic OR lipemia OR lipemias OR lipemic OR hypercholesterolemia OR hyper-cholesterolemia OR hypercholesterolemic OR hyper-cholesterolemic OR cholesterol OR hypertriglyceridemia OR hyper-triglyceridemia OR hypertriglyceridemic OR hyper-triglyceridemic OR triglycerid OR triglycerids))
#12	TITLE-ABS-KEY((**neoplas* OR cancer* OR tumor* OR tumour* OR carcinoma* OR oncolog***) AND ("heart failure" OR "heart failures" OR "chronic obstructive pulmonary disease" OR "chronic obstructive lung disease" OR "chronic obstructive pulmonary diseases" OR "chronic obstructive lung diseases" OR COPD OR asthma OR asthmatic OR diabetes OR diabetic OR hypertension OR "coronary artery disease" OR "coronary atherosclerosis" OR "coronary arteriosclerosis" OR "coronary artery diseases" OR arthritis OR arthritic OR osteoarthritis OR osteoarthritic OR periarthritis OR periarthritic OR osteoporosis OR osteoporotic OR "chronic renal insufficiency" OR "chronic kidney insufficiency" OR "chronic renal disease" OR "chronic kidney disease" OR "chronic renal insufficiencies" OR "chronic renal diseases" OR "chronic kidney failure" OR "chronic renal failure" OR "renal dialysis" OR "kidney dialysis" OR hemodialysis OR haemodialysis OR

	hyperlipidemia OR hyperlipidemias OR hyperlipidemic OR hyperlipemia OR hyperlipemias OR hyperlipemic OR lipidemia OR lipidemias OR lipidemic OR lipemia OR lipemias OR lipemic OR hypercholesterolemia OR hyper-cholesterolemia OR hypercholesterolemic OR hyper-cholesterolemic OR cholesterol OR hypertriglyceridemia OR hyper-triglyceridemia OR hypertriglyceridemic OR hyper-triglyceridemic OR triglycerid OR triglycerids))
#13	TITLE-ABS-KEY((**“chronic renal insufficiency” OR “chronic kidney insufficiency” OR “chronic renal disease” OR “chronic kidney disease” OR “chronic renal insufficiencies” OR “chronic renal diseases” OR “chronic kidney failure” OR “chronic renal failure” OR “renal dialysis” OR “kidney dialysis” OR hemodialysis OR haemodialysis**) AND (“heart failure” OR “heart failures” OR “chronic obstructive pulmonary disease” OR “chronic obstructive lung disease” OR “chronic obstructive pulmonary diseases” OR “chronic obstructive lung diseases” OR COPD OR asthma OR asthmatic OR diabetes OR diabetic OR hypertension OR “coronary artery disease” OR “coronary atherosclerosis” OR “coronary arteriosclerosis” OR “coronary artery diseases” OR arthritis OR arthritic OR osteoarthritis OR osteoarthritic OR periarthritis OR periarthritic OR osteoporosis OR osteoporotic OR neoplas* OR cancer* OR tumor* OR tumour* OR carcinoma* OR oncolog* OR hyperlipidemia OR hyperlipidemias OR hyperlipidemic OR hyperlipemia OR hyperlipemias OR hyperlipemic OR lipidemia OR lipidemias OR lipidemic OR lipemia OR lipemias OR lipemic OR hypercholesterolemia OR hyper-cholesterolemia OR hypercholesterolemic OR hyper-cholesterolemic OR cholesterol OR hypertriglyceridemia OR hyper-triglyceridemia OR hypertriglyceridemic OR hyper-triglyceridemic OR triglycerid OR triglycerids))
#14	TITLE-ABS-KEY((**hyperlipidemia OR hyperlipidemias OR hyperlipidemic OR hyperlipemia OR hyperlipemias OR hyperlipemic OR lipidemia OR lipidemias OR lipidemic OR lipemia OR lipemias OR lipemic OR hypercholesterolemia OR hyper-cholesterolemia OR hypercholesterolemic OR hyper-cholesterolemic OR cholesterol OR hypertriglyceridemia OR hyper-triglyceridemia OR hypertriglyceridemic OR hyper-triglyceridemic OR triglycerid OR triglycerids**) AND (“heart failure” OR “heart failures” OR “chronic obstructive pulmonary disease” OR “chronic obstructive lung disease” OR “chronic obstructive pulmonary diseases” OR “chronic obstructive lung diseases” OR COPD OR asthma OR asthmatic OR diabetes OR diabetic OR hypertension OR “coronary artery disease” OR “coronary atherosclerosis” OR “coronary arteriosclerosis” OR “coronary artery diseases” OR arthritis OR arthritic OR osteoarthritis OR osteoarthritic OR periarthritis OR periarthritic OR osteoporosis OR osteoporotic OR neoplas* OR cancer* OR tumor* OR tumour* OR carcinoma* OR oncolog* OR “chronic renal insufficiency” OR “chronic kidney insufficiency” OR “chronic renal disease” OR “chronic kidney disease” OR “chronic renal insufficiencies” OR “chronic renal diseases” OR “chronic kidney failure” OR “chronic renal failure” OR “renal dialysis” OR “kidney dialysis” OR hemodialysis OR haemodialysis))
**#15**	**#2 OR #3 OR #4 OR #5 OR #6 OR #7 OR #8 OR #9 OR #10 OR #11 OR #12 OR #13 OR #14**
#16	TITLE-ABS-KEY(aging OR older OR elder* OR geriatr*)
**#17**	**#1 AND #15 AND #16**

Web of Science

#1	TS=("motivational interview*" OR "motivational technique*" OR "motivational counsel*" OR "motivational enhancement therap*" OR "motivational therap*" OR "motivational intervention*" OR "motivational strateg*" OR "motivational approach*" OR "motivational communicat*" OR "motivational language*")
#2	TS=(polypharma* OR polymedicat* OR poly-pharma* OR poly-medicat* OR multimedicat* OR multi-medicat* OR plurimedicat* OR polypatholog* OR poly-patholog* OR pluripatholog* OR pluri-patholog* OR multipatholog* OR multi-patholog* OR comorbid* OR multimorbid* OR multi-morbid* OR plurimorbid* OR polymorbid* OR poly-morbid* OR concurrent* OR concomitant* OR coexist* OR co-exist*)
#3	TS=(diseases OR conditions OR disorders OR illnesses OR "health problems" AND multiple)
#4	TS=((**"heart failure" OR "heart failures")** AND ("chronic obstructive pulmonary disease" OR "chronic obstructive lung disease" OR "chronic obstructive pulmonary diseases" OR "chronic obstructive lung diseases" OR COPD OR asthma OR asthmatic OR diabetes OR diabetic OR hypertension OR "coronary artery disease" OR "coronary atherosclerosis" OR "coronary arteriosclerosis" OR "coronary artery diseases" OR arthritis OR arthritic OR osteoarthritis OR osteoarthritic OR periarthritis OR periarthritic OR osteoporosis OR osteoporotic OR neoplas* OR cancer* OR tumor* OR tumour* OR carcinoma* OR oncolog* OR "chronic renal insufficiency" OR "chronic kidney insufficiency" OR "chronic renal disease" OR "chronic kidney disease" OR "chronic renal insufficiencies" OR "chronic renal diseases" OR "chronic kidney failure" OR "chronic renal failure" OR "renal dialysis" OR "kidney dialysis" OR hemodialysis OR haemodialysis OR hyperlipidemia OR hyperlipidemias OR hyperlipidemic OR hyperlipemia OR hyperlipemias OR hyperlipemic OR lipidemia OR lipidemias OR lipidemic OR lipemia OR lipemias OR lipemic OR hypercholesterolemia OR hyper-cholesterolemia OR hypercholesterolemic OR hyper-cholesterolemic OR cholesterol OR hypertriglyceridemia OR hyper-triglyceridemia OR hypertriglyceridemic OR hyper-triglyceridemic OR triglycerid OR triglycerids))
#5	TS=((**"chronic obstructive pulmonary disease" OR "chronic obstructive lung disease" OR "chronic obstructive pulmonary diseases" OR "chronic obstructive lung diseases" OR COPD**) AND ("heart failure" OR "heart failures" OR asthma OR asthmatic OR diabetes OR diabetic OR hypertension OR "coronary artery disease" OR "coronary atherosclerosis" OR "coronary arteriosclerosis" OR "coronary artery diseases" OR arthritis OR arthritic OR osteoarthritis OR osteoarthritic OR periarthritis OR periarthritic OR osteoporosis OR osteoporotic OR neoplas* OR cancer* OR tumor* OR tumour* OR carcinoma* OR oncolog* OR "chronic renal insufficiency" OR "chronic kidney insufficiency" OR "chronic renal disease" OR "chronic kidney disease" OR "chronic renal insufficiencies" OR "chronic renal diseases" OR "chronic kidney failure" OR "chronic renal failure" OR "renal dialysis" OR "kidney dialysis" OR hemodialysis OR haemodialysis OR hyperlipidemia OR hyperlipidemias OR hyperlipidemic OR hyperlipemia OR hyperlipemias OR hyperlipemic OR lipidemia OR lipidemias OR lipidemic OR lipemia OR lipemias OR lipemic OR hypercholesterolemia OR hyper-cholesterolemia OR hypercholesterolemic OR hyper-cholesterolemic OR cholesterol OR hypertriglyceridemia OR hyper-triglyceridemia OR hypertriglyceridemic OR hyper-triglyceridemic OR triglycerid OR triglycerids))
#6	TS=((**asthma OR asthmatic**) AND ("heart failure" OR "heart failures" OR "chronic obstructive pulmonary disease" OR "chronic obstructive lung disease" OR "chronic obstructive pulmonary diseases" OR "chronic obstructive lung diseases" OR COPD OR diabetes OR diabetic OR hypertension OR "coronary artery disease" OR "coronary atherosclerosis" OR "coronary arteriosclerosis" OR "coronary artery diseases" OR arthritis

	OR arthritic OR osteoarthritis OR osteoarthritic OR periarthritis OR periarthritic OR osteoporosis OR osteoporotic OR neoplas* OR cancer* OR tumor* OR tumour* OR carcinoma* OR oncolog* OR "chronic renal insufficiency" OR "chronic kidney insufficiency" OR "chronic renal disease" OR "chronic kidney disease" OR "chronic renal insufficiencies" OR "chronic renal diseases" OR "chronic kidney failure" OR "chronic renal failure" OR "renal dialysis" OR "kidney dialysis" OR hemodialysis OR haemodialysis OR hyperlipidemia OR hyperlipidemias OR hyperlipidemic OR hyperlipemia OR hyperlipemias OR hyperlipemic OR lipidemia OR lipidemias OR lipidemic OR lipemia OR lipemias OR lipemic OR hypercholesterolemia OR hyper-cholesterolemia OR hypercholesterolemic OR hyper-cholesterolemic OR cholesterol OR hypertriglyceridemia OR hyper-triglyceridemia OR hypertriglyceridemic OR hyper-triglyceridemic OR triglycerid OR triglycerids))
#7	TS=((**diabetes OR diabetic**) AND ("heart failure" OR "heart failures" OR "chronic obstructive pulmonary disease" OR "chronic obstructive lung disease" OR "chronic obstructive pulmonary diseases" OR "chronic obstructive lung diseases" OR COPD OR asthma OR asthmatic OR hypertension OR "coronary artery disease" OR "coronary atherosclerosis" OR "coronary arteriosclerosis" OR "coronary artery diseases" OR arthritis OR arthritic OR osteoarthritis OR osteoarthritic OR periarthritis OR periarthritic OR osteoporosis OR osteoporotic OR neoplas* OR cancer* OR tumor* OR tumour* OR carcinoma* OR oncolog* OR "chronic renal insufficiency" OR "chronic kidney insufficiency" OR "chronic renal disease" OR "chronic kidney disease" OR "chronic renal insufficiencies" OR "chronic renal diseases" OR "chronic kidney failure" OR "chronic renal failure" OR "renal dialysis" OR "kidney dialysis" OR hemodialysis OR haemodialysis OR hyperlipidemia OR hyperlipidemias OR hyperlipidemic OR hyperlipemia OR hyperlipemias OR hyperlipemic OR lipidemia OR lipidemias OR lipidemic OR lipemia OR lipemias OR lipemic OR hypercholesterolemia OR hyper-cholesterolemia OR hypercholesterolemic OR hyper-cholesterolemic OR cholesterol OR hypertriglyceridemia OR hyper-triglyceridemia OR hypertriglyceridemic OR hyper-triglyceridemic OR triglycerid OR triglycerids))
#8	TS=(**hypertension** AND ("heart failure" OR "heart failures" OR "chronic obstructive pulmonary disease" OR "chronic obstructive lung disease" OR "chronic obstructive pulmonary diseases" OR "chronic obstructive lung diseases" OR COPD OR asthma OR asthmatic OR diabetes OR diabetic OR "coronary artery disease" OR "coronary atherosclerosis" OR "coronary arteriosclerosis" OR "coronary artery diseases" OR arthritis OR arthritic OR osteoarthritis OR osteoarthritic OR periarthritis OR periarthritic OR osteoporosis OR osteoporotic OR neoplas* OR cancer* OR tumor* OR tumour* OR carcinoma* OR oncolog* OR "chronic renal insufficiency" OR "chronic kidney insufficiency" OR "chronic renal disease" OR "chronic kidney disease" OR "chronic renal insufficiencies" OR "chronic renal diseases" OR "chronic kidney failure" OR "chronic renal failure" OR "renal dialysis" OR "kidney dialysis" OR hemodialysis OR haemodialysis OR hyperlipidemia OR hyperlipidemias OR hyperlipidemic OR hyperlipemia OR hyperlipemias OR hyperlipemic OR lipidemia OR lipidemias OR lipidemic OR lipemia OR lipemias OR lipemic OR hypercholesterolemia OR hyper-cholesterolemia OR hypercholesterolemic OR hyper-cholesterolemic OR cholesterol OR hypertriglyceridemia OR hyper-triglyceridemia OR hypertriglyceridemic OR hyper-triglyceridemic OR triglycerid OR triglycerids))
#9	TS=((**"coronary artery disease" OR "coronary atherosclerosis" OR "coronary arteriosclerosis" OR "coronary artery diseases"**) AND ("heart failure" OR "heart failures" OR "chronic obstructive pulmonary disease" OR "chronic obstructive lung disease" OR "chronic obstructive pulmonary diseases" OR "chronic obstructive lung diseases" OR COPD OR asthma OR asthmatic OR diabetes OR diabetic OR hypertension

	OR arthritis OR arthritic OR osteoarthritis OR osteoarthritic OR periarthritis OR periarthritic OR osteoporosis OR osteoporotic OR neoplas* OR cancer* OR tumor* OR tumour* OR carcinoma* OR oncolog* OR “chronic renal insufficiency” OR “chronic kidney insufficiency” OR “chronic renal disease” OR “chronic kidney disease” OR “chronic renal insufficiencies” OR “chronic renal diseases” OR “chronic kidney failure” OR “chronic renal failure” OR “renal dialysis” OR “kidney dialysis” OR hemodialysis OR haemodialysis OR hyperlipidemia OR hyperlipidemias OR hyperlipidemic OR hyperlipemia OR hyperlipemias OR hyperlipemic OR lipidemia OR lipidemias OR lipidemic OR lipemia OR lipemias OR lipemic OR hypercholesterolemia OR hyper-cholesterolemia OR hypercholesterolemic OR hyper-cholesterolemic OR cholesterol OR hypertriglyceridemia OR hyper-triglyceridemia OR hypertriglyceridemic OR hyper-triglyceridemic OR triglycerid OR triglycerids))
#10	TS=((**arthritis OR arthritic OR osteoarthritis OR osteoarthritic OR periarthritis OR periarthritic**) AND (“heart failure” OR “heart failures” OR “chronic obstructive pulmonary disease” OR “chronic obstructive lung disease” OR “chronic obstructive pulmonary diseases” OR “chronic obstructive lung diseases” OR COPD OR asthma OR asthmatic OR diabetes OR diabetic OR hypertension OR “coronary artery disease” OR “coronary atherosclerosis” OR “coronary arteriosclerosis” OR “coronary artery diseases” OR osteoporosis OR osteoporotic OR neoplas* OR cancer* OR tumor* OR tumour* OR carcinoma* OR oncolog* OR “chronic renal insufficiency” OR “chronic kidney insufficiency” OR “chronic renal disease” OR “chronic kidney disease” OR “chronic renal insufficiencies” OR “chronic renal diseases” OR “chronic kidney failure” OR “chronic renal failure” OR “renal dialysis” OR “kidney dialysis” OR hemodialysis OR haemodialysis OR hyperlipidemia OR hyperlipidemias OR hyperlipidemic OR hyperlipemia OR hyperlipemias OR hyperlipemic OR lipidemia OR lipidemias OR lipidemic OR lipemia OR lipemias OR lipemic OR hypercholesterolemia OR hyper-cholesterolemia OR hypercholesterolemic OR hyper-cholesterolemic OR cholesterol OR hypertriglyceridemia OR hyper-triglyceridemia OR hypertriglyceridemic OR hyper-triglyceridemic OR triglycerid OR triglycerids))
#11	TS=((**osteoporosis OR osteoporotic**) AND (“heart failure” OR “heart failures” OR “chronic obstructive pulmonary disease” OR “chronic obstructive lung disease” OR “chronic obstructive pulmonary diseases” OR “chronic obstructive lung diseases” OR COPD OR asthma OR asthmatic OR diabetes OR diabetic OR hypertension OR “coronary artery disease” OR “coronary atherosclerosis” OR “coronary arteriosclerosis” OR “coronary artery diseases” OR arthritis OR arthritic OR osteoarthritis OR osteoarthritic OR periarthritis OR periarthritic OR neoplas* OR cancer* OR tumor* OR tumour* OR carcinoma* OR oncolog* OR “chronic renal insufficiency” OR “chronic kidney insufficiency” OR “chronic renal disease” OR “chronic kidney disease” OR “chronic renal insufficiencies” OR “chronic renal diseases” OR “chronic kidney failure” OR “chronic renal failure” OR “renal dialysis” OR “kidney dialysis” OR hemodialysis OR haemodialysis OR hyperlipidemia OR hyperlipidemias OR hyperlipidemic OR hyperlipemia OR hyperlipemias OR hyperlipemic OR lipidemia OR lipidemias OR lipidemic OR lipemia OR lipemias OR lipemic OR hypercholesterolemia OR hyper-cholesterolemia OR hypercholesterolemic OR hyper-cholesterolemic OR cholesterol OR hypertriglyceridemia OR hyper-triglyceridemia OR hypertriglyceridemic OR hyper-triglyceridemic OR triglycerid OR triglycerids))
#12	TS=((**neoplas* OR cancer* OR tumor* OR tumour* OR carcinoma* OR oncolog***) AND (“heart failure” OR “heart failures” OR “chronic obstructive pulmonary disease” OR “chronic obstructive lung disease” OR “chronic obstructive pulmonary diseases” OR “chronic obstructive lung diseases” OR COPD OR asthma OR asthmatic OR diabetes OR diabetic OR hypertension OR “coronary artery disease” OR “coronary atherosclerosis” OR

	“coronary arteriosclerosis” OR “coronary artery diseases” OR arthritis OR arthritic OR osteoarthritis OR osteoarthritic OR periarthritis OR periarthritic OR osteoporosis OR osteoporotic OR “chronic renal insufficiency” OR “chronic kidney insufficiency” OR “chronic renal disease” OR “chronic kidney disease” OR “chronic renal insufficiencies” OR “chronic renal diseases” OR “chronic kidney failure” OR “chronic renal failure” OR “renal dialysis” OR “kidney dialysis” OR hemodialysis OR haemodialysis OR hyperlipidemia OR hyperlipidemias OR hyperlipidemic OR hyperlipemia OR hyperlipemias OR hyperlipemic OR lipidemia OR lipidemias OR lipidemic OR lipemia OR lipemias OR lipemic OR hypercholesterolemia OR hyper-cholesterolemia OR hypercholesterolemic OR hyper-cholesterolemic OR cholesterol OR hypertriglyceridemia OR hyper-triglyceridemia OR hypertriglyceridemic OR hyper-triglyceridemic OR triglycerid OR triglycerids))
#13	TS=((**“chronic renal insufficiency” OR “chronic kidney insufficiency” OR “chronic renal disease” OR “chronic kidney disease” OR “chronic renal insufficiencies” OR “chronic renal diseases” OR “chronic kidney failure” OR “chronic renal failure” OR “renal dialysis” OR “kidney dialysis” OR hemodialysis OR haemodialysis**) AND (“heart failure” OR “heart failures” OR “chronic obstructive pulmonary disease” OR “chronic obstructive lung disease” OR “chronic obstructive pulmonary diseases” OR “chronic obstructive lung diseases” OR COPD OR asthma OR asthmatic OR diabetes OR diabetic OR hypertension OR “coronary artery disease” OR “coronary atherosclerosis” OR “coronary arteriosclerosis” OR “coronary artery diseases” OR arthritis OR arthritic OR osteoarthritis OR osteoarthritic OR periarthritis OR periarthritic OR osteoporosis OR osteoporotic OR neoplas* OR cancer* OR tumor* OR tumour* OR carcinoma* OR oncolog* OR hyperlipidemia OR hyperlipidemias OR hyperlipidemic OR hyperlipemia OR hyperlipemias OR hyperlipemic OR lipidemia OR lipidemias OR lipidemic OR lipemia OR lipemias OR lipemic OR hypercholesterolemia OR hyper-cholesterolemia OR hypercholesterolemic OR hyper-cholesterolemic OR cholesterol OR hypertriglyceridemia OR hyper-triglyceridemia OR hypertriglyceridemic OR hyper-triglyceridemic OR triglycerid OR triglycerids))
#14	TS=((**hyperlipidemia OR hyperlipidemias OR hyperlipidemic OR hyperlipemia OR hyperlipemias OR hyperlipemic OR lipidemia OR lipidemias OR lipidemic OR lipemia OR lipemias OR lipemic OR hypercholesterolemia OR hyper-cholesterolemia OR hypercholesterolemic OR hyper-cholesterolemic OR cholesterol OR hypertriglyceridemia OR hyper-triglyceridemia OR hypertriglyceridemic OR hyper-triglyceridemic OR triglycerid OR triglycerids**) AND (“heart failure” OR “heart failures” OR “chronic obstructive pulmonary disease” OR “chronic obstructive lung disease” OR “chronic obstructive pulmonary diseases” OR “chronic obstructive lung diseases” OR COPD OR asthma OR asthmatic OR diabetes OR diabetic OR hypertension OR “coronary artery disease” OR “coronary atherosclerosis” OR “coronary arteriosclerosis” OR “coronary artery diseases” OR arthritis OR arthritic OR osteoarthritis OR osteoarthritic OR periarthritis OR periarthritic OR osteoporosis OR osteoporotic OR neoplas* OR cancer* OR tumor* OR tumour* OR carcinoma* OR oncolog* OR “chronic renal insufficiency” OR “chronic kidney insufficiency” OR “chronic renal disease” OR “chronic kidney disease” OR “chronic renal insufficiencies” OR “chronic renal diseases” OR “chronic kidney failure” OR “chronic renal failure” OR “renal dialysis” OR “kidney dialysis” OR hemodialysis OR haemodialysis))
**#15**	**#2 OR #3 OR #4 OR #5 OR #6 OR #7 OR #8 OR #9 OR #10 OR #11 OR #12 OR #13 OR #14**
#16	TS=(aging OR older OR elder* OR geriatr*)
**#17**	**#1 AND #15 AND #16**

Cochrane CENTRAL Register of Controlled Trials

#1	MeSH descriptor: [Motivational Interviewing] explode all trees
#2	MeSH descriptor: [Motivation] explode all trees
#3	MeSH descriptor: [Patient Education as Topic] explode all trees
#4	#2 AND #3
#5	#1 OR #4
#6	((motivational NEXT interview*) OR (motivational NEXT technique*) OR (motivational NEXT counsel*) OR (motivational NEXT enhancement NEXT therap*) OR (motivational NEXT therap*) OR (motivational NEXT intervention*) OR (motivational NEXT strateg*) OR (motivational NEXT approach*) OR (motivational NEXT communicat*) OR (motivational NEXT language*)):ti,ab,kw
**#7**	**#5 OR #6**
#8	MeSH descriptor: [Polypharmacy] explode all trees
#9	MeSH descriptor: [Comorbidity] explode all trees
#10	MeSH descriptor: [Multiple Chronic Conditions] explode all trees
#11	(polypharma* OR polymedicat* OR poly-pharma* OR poly-medicat* OR multimedicat* OR multi-medicat* OR plurimedicat* OR polypatholog* OR poly-patholog* OR pluripatholog* OR pluri-patholog* OR multipatholog* OR multi-patholog* OR comorbid* OR multimorbid* OR multi-morbid* OR plurimorbid* OR polymorbid* OR poly-morbid* OR concurrent* OR concomitant* OR coexist* OR co-exist*):ti,ab,kw
#12	MeSH descriptor: [Chronic Disease] this term only
#13	(diseases OR conditions OR disorders OR illnesses OR “health problems”):ti,ab,kw
#14	#12 OR #13
#15	(multiple):ti,ab,kw
#16	#15 AND #14
#17	MeSH descriptor: [Heart Failure] explode all trees
#18	MeSH descriptor: [Pulmonary Disease, Chronic Obstructive] explode all trees
#19	MeSH descriptor: [Asthma] explode all trees
#20	MeSH descriptor: [Diabetes Mellitus] explode all trees
#21	MeSH descriptor: [Hypertension] explode all trees
#22	MeSH descriptor: [Coronary Artery Disease] explode all trees
#23	MeSH descriptor: [Arthritis] explode all trees
#24	MeSH descriptor: [Osteoporosis] explode all trees
#25	MeSH descriptor: [Neoplasms] explode all trees
#26	MeSH descriptor: [Renal Insufficiency, Chronic] explode all trees
#27	MeSH descriptor: [Renal Dialysis] explode all trees
#28	MeSH descriptor: [Hyperlipidemias] explode all trees

#29	MeSH descriptor: [Cholesterol] explode all trees
#30	MeSH descriptor: [Triglycerides] explode all trees
#31	#17 AND (#18 OR #19 OR #20 OR #21 OR #22 OR #23 OR #24 OR #25 OR #26 OR #27 OR #28 OR #29 OR #30)
#32	#18 AND (#17 OR #19 OR #20 OR #21 OR #22 OR #23 OR #24 OR #25 OR #26 OR #27 OR #28 OR #29 OR #30)
#33	#19 AND (#17 OR #18 OR #20 OR #21 OR #22 OR #23 OR #24 OR #25 OR #26 OR #27 OR #28 OR #29 OR #30)
#34	#20 AND (#17 OR #18 OR #19 OR #21 OR #22 OR #23 OR #24 OR #25 OR #26 OR #27 OR #28 OR #29 OR #30)
#35	#21 AND (#17 OR #18 OR #19 OR #20 OR #22 OR #23 OR #24 OR #25 OR #26 OR #27 OR #28 OR #29 OR #30)
#36	#22 AND (#17 OR #18 OR #19 OR #20 OR #21 OR #23 OR #24 OR #25 OR #26 OR #27 OR #28 OR #29 OR #30)
#37	#23 AND (#17 OR #18 OR #19 OR #20 OR #21 OR #22 OR #24 OR #25 OR #26 OR #27 OR #28 OR #29 OR #30)
#38	#24 AND (#17 OR #18 OR #19 OR #20 OR #21 OR #22 OR #23 OR #25 OR #26 OR #27 OR #28 OR #29 OR #30)
#39	#25 AND (#17 OR #18 OR #19 OR #20 OR #21 OR #22 OR #23 OR #24 OR #26 OR #27 OR #28 OR #29 OR #30)
#40	(#26 OR #27) AND (#17 OR #18 OR #19 OR #20 OR #21 OR #22 OR #23 OR #24 OR #25 OR #28 OR #29 OR #30)
#41	(#28 OR #29 OR #30) AND (#17 OR #18 OR #19 OR #20 OR #21 OR #22 OR #23 OR #24 OR #25 OR #26 OR #27)
#42	((“heart failure” OR “heart failures”) AND (“chronic obstructive pulmonary disease” OR “chronic obstructive lung disease” OR “chronic obstructive pulmonary diseases” OR “chronic obstructive lung diseases” OR COPD OR asthma OR asthmatic OR diabetes OR diabetic OR hypertension OR “coronary artery disease” OR “coronary atherosclerosis” OR “coronary arteriosclerosis” OR “coronary artery diseases” OR arthritis OR arthritic OR osteoarthritis OR osteoarthritic OR periarthritis OR periarthritic OR osteoporosis OR osteoporotic OR neoplas* OR cancer* OR tumor* OR tumour* OR carcinoma* OR oncolog* OR “chronic renal insufficiency” OR “chronic kidney insufficiency” OR “chronic renal disease” OR “chronic kidney disease” OR “chronic renal insufficiencies” OR “chronic renal diseases” OR “chronic kidney failure” OR “chronic renal failure” OR “renal dialysis” OR “kidney dialysis” OR hemodialysis OR haemodialysis OR hyperlipidemia OR hyperlipidemias OR hyperlipidemic OR hyperlipemia OR hyperlipemias OR hyperlipemic OR lipidemia OR lipidemias OR lipidemic OR lipemia OR lipemias OR lipemic OR hypercholesterolemia OR hyper-cholesterolemia OR hypercholesterolemic OR hyper-cholesterolemic OR cholesterol OR hypertriglyceridemia OR hyper-triglyceridemia OR hypertriglyceridemic OR hyper-triglyceridemic OR triglycerid OR triglycerids)):ti,ab,kw
#43	((“chronic obstructive pulmonary disease” OR “chronic obstructive lung disease” OR “chronic obstructive pulmonary diseases” OR “chronic obstructive lung diseases” OR COPD) AND (“heart failure” OR “heart failures” OR asthma OR asthmatic OR diabetes OR diabetic OR hypertension OR “coronary artery disease” OR “coronary

	atherosclerosis" OR "coronary arteriosclerosis" OR "coronary artery diseases" OR arthritis OR arthritic OR osteoarthritis OR osteoarthritic OR periarthritis OR periarthritic OR osteoporosis OR osteoporotic OR neoplas* OR cancer* OR tumor* OR tumour* OR carcinoma* OR oncolog* OR "chronic renal insufficiency" OR "chronic kidney insufficiency" OR "chronic renal disease" OR "chronic kidney disease" OR "chronic renal insufficiencies" OR "chronic renal diseases" OR "chronic kidney failure" OR "chronic renal failure" OR "renal dialysis" OR "kidney dialysis" OR hemodialysis OR haemodialysis OR hyperlipidemia OR hyperlipidemias OR hyperlipidemic OR hyperlipemia OR hyperlipemias OR hyperlipemic OR lipidemia OR lipidemias OR lipidemic OR lipemia OR lipemias OR lipemic OR hypercholesterolemia OR hyper-cholesterolemia OR hypercholesterolemic OR hyper-cholesterolemic OR cholesterol OR hypertriglyceridemia OR hyper-triglyceridemia OR hypertriglyceridemic OR hyper-triglyceridemic OR triglycerid OR triglycerids)):ti,ab,kw
#44	((asthma OR asthmatic) AND ("heart failure" OR "heart failures" OR "chronic obstructive pulmonary disease" OR "chronic obstructive lung disease" OR "chronic obstructive pulmonary diseases" OR "chronic obstructive lung diseases" OR COPD OR diabetes OR diabetic OR hypertension OR "coronary artery disease" OR "coronary atherosclerosis" OR "coronary arteriosclerosis" OR "coronary artery diseases" OR arthritis OR arthritic OR osteoarthritis OR osteoarthritic OR periarthritis OR periarthritic OR osteoporosis OR osteoporotic OR neoplas* OR cancer* OR tumor* OR tumour* OR carcinoma* OR oncolog* OR "chronic renal insufficiency" OR "chronic kidney insufficiency" OR "chronic renal disease" OR "chronic kidney disease" OR "chronic renal insufficiencies" OR "chronic renal diseases" OR "chronic kidney failure" OR "chronic renal failure" OR "renal dialysis" OR "kidney dialysis" OR hemodialysis OR haemodialysis OR hyperlipidemia OR hyperlipidemias OR hyperlipidemic OR hyperlipemia OR hyperlipemias OR hyperlipemic OR lipidemia OR lipidemias OR lipidemic OR lipemia OR lipemias OR lipemic OR hypercholesterolemia OR hyper-cholesterolemia OR hypercholesterolemic OR hyper-cholesterolemic OR cholesterol OR hypertriglyceridemia OR hyper-triglyceridemia OR hypertriglyceridemic OR hyper-triglyceridemic OR triglycerid OR triglycerids)):ti,ab,kw
#45	((diabetes OR diabetic) AND ("heart failure" OR "heart failures" OR "chronic obstructive pulmonary disease" OR "chronic obstructive lung disease" OR "chronic obstructive pulmonary diseases" OR "chronic obstructive lung diseases" OR COPD OR asthma OR asthmatic OR hypertension OR "coronary artery disease" OR "coronary atherosclerosis" OR "coronary arteriosclerosis" OR "coronary artery diseases" OR arthritis OR arthritic OR osteoarthritis OR osteoarthritic OR periarthritis OR periarthritic OR osteoporosis OR osteoporotic OR neoplas* OR cancer* OR tumor* OR tumour* OR carcinoma* OR oncolog* OR "chronic renal insufficiency" OR "chronic kidney insufficiency" OR "chronic renal disease" OR "chronic kidney disease" OR "chronic renal insufficiencies" OR "chronic renal diseases" OR "chronic kidney failure" OR "chronic renal failure" OR "renal dialysis" OR "kidney dialysis" OR hemodialysis OR haemodialysis OR hyperlipidemia OR hyperlipidemias OR hyperlipidemic OR hyperlipemia OR hyperlipemias OR hyperlipemic OR lipidemia OR lipidemias OR lipidemic OR lipemia OR lipemias OR lipemic OR hypercholesterolemia OR hyper-cholesterolemia OR hypercholesterolemic OR hyper-cholesterolemic OR cholesterol OR hypertriglyceridemia OR hyper-triglyceridemia OR hypertriglyceridemic OR hyper-triglyceridemic OR triglycerid OR triglycerids)):ti,ab,kw

#46	(hypertension AND ("heart failure" OR "heart failures" OR "chronic obstructive pulmonary disease" OR "chronic obstructive lung disease" OR "chronic obstructive pulmonary diseases" OR "chronic obstructive lung diseases" OR COPD OR asthma OR asthmatic OR diabetes OR diabetic OR "coronary artery disease" OR "coronary atherosclerosis" OR "coronary arteriosclerosis" OR "coronary artery diseases" OR arthritis OR arthritic OR osteoarthritis OR osteoarthritic OR periarthritis OR periarthritic OR osteoporosis OR osteoporotic OR neoplas* OR cancer* OR tumor* OR tumour* OR carcinoma* OR oncolog* OR "chronic renal insufficiency" OR "chronic kidney insufficiency" OR "chronic renal disease" OR "chronic kidney disease" OR "chronic renal insufficiencies" OR "chronic renal diseases" OR "chronic kidney failure" OR "chronic renal failure" OR "renal dialysis" OR "kidney dialysis" OR hemodialysis OR haemodialysis OR hyperlipidemia OR hyperlipidemias OR hyperlipidemic OR hyperlipemia OR hyperlipemias OR hyperlipemic OR lipidemia OR lipidemias OR lipidemic OR lipemia OR lipemias OR lipemic OR hypercholesterolemia OR hyper-cholesterolemia OR hypercholesterolemic OR hyper-cholesterolemic OR cholesterol OR hypertriglyceridemia OR hyper-triglyceridemia OR hypertriglyceridemic OR hyper-triglyceridemic OR triglycerid OR triglycerids)):ti,ab,kw
#47	(("coronary artery disease" OR "coronary atherosclerosis" OR "coronary arteriosclerosis" OR "coronary artery diseases") AND ("heart failure" OR "heart failures" OR "chronic obstructive pulmonary disease" OR "chronic obstructive lung disease" OR "chronic obstructive pulmonary diseases" OR "chronic obstructive lung diseases" OR COPD OR asthma OR asthmatic OR diabetes OR diabetic OR hypertension OR arthritis OR arthritic OR osteoarthritis OR osteoarthritic OR periarthritis OR periarthritic OR osteoporosis OR osteoporotic OR neoplas* OR cancer* OR tumor* OR tumour* OR carcinoma* OR oncolog* OR "chronic renal insufficiency" OR "chronic kidney insufficiency" OR "chronic renal disease" OR "chronic kidney disease" OR "chronic renal insufficiencies" OR "chronic renal diseases" OR "chronic kidney failure" OR "chronic renal failure" OR "renal dialysis" OR "kidney dialysis" OR hemodialysis OR haemodialysis OR hyperlipidemia OR hyperlipidemias OR hyperlipidemic OR hyperlipemia OR hyperlipemias OR hyperlipemic OR lipidemia OR lipidemias OR lipidemic OR lipemia OR lipemias OR lipemic OR hypercholesterolemia OR hyper-cholesterolemia OR hypercholesterolemic OR hyper-cholesterolemic OR cholesterol OR hypertriglyceridemia OR hyper-triglyceridemia OR hypertriglyceridemic OR hyper-triglyceridemic OR triglycerid OR triglycerids)):ti,ab,kw
#48	((arthritis OR arthritic OR osteoarthritis OR osteoarthritic OR periarthritis OR periarthritic) AND ("heart failure" OR "heart failures" OR "chronic obstructive pulmonary disease" OR "chronic obstructive lung disease" OR "chronic obstructive pulmonary diseases" OR "chronic obstructive lung diseases" OR COPD OR asthma OR asthmatic OR diabetes OR diabetic OR hypertension OR "coronary artery disease" OR "coronary atherosclerosis" OR "coronary arteriosclerosis" OR "coronary artery diseases" OR osteoporosis OR osteoporotic OR neoplas* OR cancer* OR tumor* OR tumour* OR carcinoma* OR oncolog* OR "chronic renal insufficiency" OR "chronic kidney insufficiency" OR "chronic renal disease" OR "chronic kidney disease" OR "chronic renal insufficiencies" OR "chronic renal diseases" OR "chronic kidney failure" OR "chronic renal failure" OR "renal dialysis" OR "kidney dialysis" OR hemodialysis OR haemodialysis OR hyperlipidemia OR hyperlipidemias OR hyperlipidemic OR hyperlipemia OR hyperlipemias OR hyperlipemic OR lipidemia OR lipidemias OR lipidemic OR lipemia OR lipemias OR lipemic OR hypercholesterolemia OR hyper-cholesterolemia OR hypercholesterolemic OR hyper-

	cholesterolemic OR cholesterol OR hypertriglyceridemia OR hyper-triglyceridemia OR hypertriglyceridemic OR hyper-triglyceridemic OR triglycerid OR triglycerids)):ti,ab,kw
#49	((osteoporosis OR osteoporotic) AND (“heart failure” OR “heart failures” OR “chronic obstructive pulmonary disease” OR “chronic obstructive lung disease” OR “chronic obstructive pulmonary diseases” OR “chronic obstructive lung diseases” OR COPD OR asthma OR asthmatic OR diabetes OR diabetic OR hypertension OR “coronary artery disease” OR “coronary atherosclerosis” OR “coronary arteriosclerosis” OR “coronary artery diseases” OR arthritis OR arthritic OR osteoarthritis OR osteoarthritic OR periarthritis OR periarthritic OR neoplas* OR cancer* OR tumor* OR tumour* OR carcinoma* OR oncolog* OR “chronic renal insufficiency” OR “chronic kidney insufficiency” OR “chronic renal disease” OR “chronic kidney disease” OR “chronic renal insufficiencies” OR “chronic renal diseases” OR “chronic kidney failure” OR “chronic renal failure” OR “renal dialysis” OR “kidney dialysis” OR hemodialysis OR haemodialysis OR hyperlipidemia OR hyperlipidemias OR hyperlipidemic OR hyperlipemia OR hyperlipemias OR hyperlipemic OR lipidemia OR lipidemias OR lipidemic OR lipemia OR lipemias OR lipemic OR hypercholesterolemia OR hyper-cholesterolemia OR hypercholesterolemic OR hyper-cholesterolemic OR cholesterol OR hypertriglyceridemia OR hyper-triglyceridemia OR hypertriglyceridemic OR hyper-triglyceridemic OR triglycerid OR triglycerids)):ti,ab,kw
#50	((neoplas* OR cancer* OR tumor* OR tumour* OR carcinoma* OR oncolog*) AND (“heart failure” OR “heart failures” OR “chronic obstructive pulmonary disease” OR “chronic obstructive lung disease” OR “chronic obstructive pulmonary diseases” OR “chronic obstructive lung diseases” OR COPD OR asthma OR asthmatic OR diabetes OR diabetic OR hypertension OR “coronary artery disease” OR “coronary atherosclerosis” OR “coronary arteriosclerosis” OR “coronary artery diseases” OR arthritis OR arthritic OR osteoarthritis OR osteoarthritic OR periarthritis OR periarthritic OR osteoporosis OR osteoporotic OR “chronic renal insufficiency” OR “chronic kidney insufficiency” OR “chronic renal disease” OR “chronic kidney disease” OR “chronic renal insufficiencies” OR “chronic renal diseases” OR “chronic kidney failure” OR “chronic renal failure” OR “renal dialysis” OR “kidney dialysis” OR hemodialysis OR haemodialysis OR hyperlipidemia OR hyperlipidemias OR hyperlipidemic OR hyperlipemia OR hyperlipemias OR hyperlipemic OR lipidemia OR lipidemias OR lipidemic OR lipemia OR lipemias OR lipemic OR hypercholesterolemia OR hyper-cholesterolemia OR hypercholesterolemic OR hyper-cholesterolemic OR cholesterol OR hypertriglyceridemia OR hyper-triglyceridemia OR hypertriglyceridemic OR hyper-triglyceridemic OR triglycerid OR triglycerids)):ti,ab,kw
#51	((“chronic renal insufficiency” OR “chronic kidney insufficiency” OR “chronic renal disease” OR “chronic kidney disease” OR “chronic renal insufficiencies” OR “chronic renal diseases” OR “chronic kidney failure” OR “chronic renal failure” OR “renal dialysis” OR “kidney dialysis” OR hemodialysis OR haemodialysis) AND (“heart failure” OR “heart failures” OR “chronic obstructive pulmonary disease” OR “chronic obstructive lung disease” OR “chronic obstructive pulmonary diseases” OR “chronic obstructive lung diseases” OR COPD OR asthma OR asthmatic OR diabetes OR diabetic OR hypertension OR “coronary artery disease” OR “coronary atherosclerosis” OR “coronary arteriosclerosis” OR “coronary artery diseases” OR arthritis OR arthritic OR osteoarthritis OR osteoarthritic OR periarthritis OR periarthritic OR osteoporosis OR osteoporotic OR neoplas* OR cancer* OR tumor* OR tumour* OR carcinoma* OR oncolog* OR hyperlipidemia OR hyperlipidemias OR hyperlipidemic OR hyperlipemia OR hyperlipemias OR hyperlipemic OR lipidemia OR lipidemias OR

	lipidemic OR lipemia OR lipemias OR lipemic OR hypercholesterolemia OR hyper-cholesterolemia OR hypercholesterolemic OR hyper-cholesterolemic OR cholesterol OR hypertriglyceridemia OR hyper-triglyceridemia OR hypertriglyceridemic OR hyper-triglyceridemic OR triglycerid OR triglycerids)):ti,ab,kw
#52	((hyperlipidemia OR hyperlipidemias OR hyperlipidemic OR hyperlipemia OR hyperlipemias OR hyperlipemic OR lipidemia OR lipidemias OR lipidemic OR lipemia OR lipemias OR lipemic OR hypercholesterolemia OR hyper-cholesterolemia OR hypercholesterolemic OR hyper-cholesterolemic OR cholesterol OR hypertriglyceridemia OR hyper-triglyceridemia OR hypertriglyceridemic OR hyper-triglyceridemic OR triglycerid OR triglycerids) AND ("heart failure" OR "heart failures" OR "chronic obstructive pulmonary disease" OR "chronic obstructive lung disease" OR "chronic obstructive pulmonary diseases" OR "chronic obstructive lung diseases" OR COPD OR asthma OR asthmatic OR diabetes OR diabetic OR hypertension OR "coronary artery disease" OR "coronary atherosclerosis" OR "coronary arteriosclerosis" OR "coronary artery diseases" OR arthritis OR arthritic OR osteoarthritis OR osteoarthritic OR periarthritis OR periarthritic OR osteoporosis OR osteoporotic OR neoplas* OR cancer* OR tumor* OR tumour* OR carcinoma* OR oncolog* OR "chronic renal insufficiency" OR "chronic kidney insufficiency" OR "chronic renal disease" OR "chronic kidney disease" OR "chronic renal insufficiencies" OR "chronic renal diseases" OR "chronic kidney failure" OR "chronic renal failure" OR "renal dialysis" OR "kidney dialysis" OR hemodialysis OR haemodialysis)):ti,ab,kw
**#53**	**#8 OR #9 OR #10 OR #11 OR #16 OR #31 OR #32 OR #33 OR #34 OR #35 OR #36 OR #37 OR #38 OR #39 OR #40 OR #41 OR #42 OR #43 OR #44 OR #45 OR #46 OR #47 OR #48 OR #49 OR #50 OR #51 OR #52**
#54	MeSH descriptor: [Aged] explode all trees
#55	(aging OR older OR elder* OR geriatr*):ti,ab,kw
**#56**	**#54 OR #55**
**#57**	**#7 AND #53 AND #56**
