# Supplementary material for: Use of Motivational Interviewing in Older Patients with Multiple Chronic Conditions and Their Informal Caregivers: A Scoping Review
Source: Healthcare (Basel). 2023 Jun 7;11(12):1681. doi: 10.3390/healthcare11121681 (PMC10297925; doi:10.3390/healthcare11121681)
Supplement: Supplementary file 1 [file healthcare-11-01681-s001.zip › Supplementary File 3.pdf]

Supplementary File 3. List of excluded articles with reasons

Articles	Reasons
Abughosh, S. M., Vadhariya, A., Johnson, M. L., Essien, E. J., Esse, T. W., Serna, O., Gallardo, E., Boklage, S. H., Choi, J., Holstad, M. M., Fleming, M. L., 2019. Enhancing Statin Adherence Using a Motivational Interviewing Intervention and Past Adherence Trajectories in Patients with Suboptimal Adherence. J. Manag. Care Spec. Pharm. 25(10), 1053–1062. https://doi.org/10.18553/jmcp.2019.25.10.1053	Age under 65
Bennett, J. A., Perrin, N. A., Hanson, G., Bennett, D., Gaynor, W., Flaherty-Robb, M., Joseph, C., Butterworth, S., Potempa, K., 2005. Healthy aging demonstration project: nurse coaching for behavior change in older adults. Res. Nurs. Health. 28(3), 187–197. https://doi.org/10.1002/nur.20077	No MCCs
Härter, M., Dirmaier, J., Dwinger, S., Kriston, L., Herbarth, L., Siegmund-Schultze, E., Bermejo, I., Matschinger, H., Heider, D., König, H.H., 2016. Effectiveness of telephone-based health coaching for patients with chronic conditions: a Randomised Controlled Trial. PLoS One. 15;11(9):e0161269. https://doi.org/10.1371/journal.pone.0161269.	Age under 65
Hochhalter, A.K. Song J., Rush J., Sklar L, Stevens A., 2020. Making the most of your healthcare intervention for older adults with multiple chronic illnesses. Patient. Educ. Counsel. 81, 207-213. https://doi.org/10.1016/j.pec.2010.01.018	No motivational interviewing
Huang, B., Li, Z., Wang, Y., Xia, J., Shi, T., Jiang, J., Nolan, M. T., Li, X., Nigwekar, S. U., Chen, L., 2018. Effectiveness of self-management support in maintenance haemodialysis patients with hypertension: A pilot cluster randomized controlled trial. Nephrology. 23(8), 755–763. https://doi.org/10.1111/nep.13098	Age under 65
Kolt, G. S., Schofield, G. M., Kerse, N., Garrett, N., Oliver, M., 2007. Effect of telephone counseling on physical activity for low-active older people in primary care: A randomized, controlled trial. J. Am. Geriatr. Soc. 55, 986-992. https://doi.org/10.1111/j.1532-5415.2007.01203.x	No MCCs
Kolt, G. S., Oliver, M., Schofield, G. M., Kerse, N., Garrett, N., & Latham, N. K., 2006. An overview and process evaluation of TeleWalk: a telephone-based counseling intervention to encourage walking in older adults. Health Promot. Int. 21(3): 201-208. https://doi.org/10.1093/heapro/dal015	No MCCs
Kuipers, S. J., Nieboer, A. P., Cramm, J. M., 2020. The Need for Co-Creation of Care with Multi-Morbidity Patients-A Longitudinal Perspective. Int. J. Environ. Res. Public Health. 17(9), 3201. https://doi.org/10.3390/ijerph17093201	Age under 65
Lawrence, D. B., Allison, W., Chen, J. C., Demand, M., 2008. Improving medication adherence with a targeted, technology-driven disease management intervention. Dis. Manag. 11(3), 141–144. https://doi.org/10.1089/dis.2007.0013	Age under 65
Lo, Y. P., Chiang, S. L., Lin, C. H., Liu, H. C., Chiang, L. C., 2020. Effects of individualized aerobic exercise training on physical activity and health-related physical fitness among middle-aged and older adults with multimorbidity: A Randomized Controlled Trial. Int. J. Environ. Res. Public. Health. 18(1), 101. https://doi.org/10.3390/ijerph18010101	Age under 65

Articles	Reasons
McKenzie, K. J., Pierce, D., Mercer, S. W., Gunn, J. M., 2021. Do GPs use motivational interviewing skills in routine consultations with patients living with mental-physical multimorbidity? An observational study of primary care in Scotland. Chronic Illn. 17(1), 29–40. https://doi.org/10.1177/1742395318815960	Age under 65
McKenzie, K. J., Pierce, D., Gunn, J. M., 2018. Guiding patients through complexity: motivational interviewing for patients with multimorbidity. Aust. J. Gen. Pract. 47(1-2), 8–13. https://doi.org/10.31128/AFP-08-17-4325	Discussion article
Mohan, A., Vadhariya, A., Majd, Z., Esse, T. W., Serna, O., Abughosh, S. M., 2021. Impact of a motivational interviewing intervention targeting statins on adherence to concurrent hypertension or diabetes medications. Patient Educ. Counsel. 104(7), 1756–1764. https://doi.org/10.1016/j.pec.2020.12.009	Age under 65
Naik, A. D., Dindo, L. N., Van Liew, J. R., Hundt, N. E., Vo, L., Hernandez-Bigos, K., Esterson, J., Geda, M., Rosen, J., Blaum, C. S., Tinetti, M. E., 2018. Development of a clinically feasible process for identifying individual health priorities. J. Am. Geriatr. Soc. 66(10), 1872–1879. https://doi.org/10.1111/jgs.15437	No motivational interviewing
Pladevall, M., Divine, G., Wells, K. E., Resnicow, K., Williams, L. K., 2015. A randomized controlled trial to provide adherence information and motivational interviewing to improve diabetes and lipid control. Diabetes Educ. 41(1), 136–146. https://doi.org/10.1177/0145721714561031	Age under 65
Riegel, B., Masterson Creber, R., Hill, J., Chittams, J., Hoke, L., 2016. Effectiveness of motivational interviewing in decreasing hospital readmission in adults with heart failure and multimorbidity. Clin. Nurs. Res. 25(4), 362–377. https://doi.org/10.1177/1054773815623252	Age under 65
Salisbury, C., Man, M.S., Bower, P., Guthrie, B., Chaplin, K., Gaunt, D.M., Brookes, S., Fitzpatrick, B., Gardner, C., Hollinghurst, S., Lee, V., McLeod, J., Mann, C., Moffat, K.R., Mercer, S.W., 2018. Management of multimorbidity using a patient-centred care model: a pragmatic cluster-randomised trial of the 3D approach. Lancet, 392, 41-50. https://doi.org/10.1016/S0140-6736(18)31308-4	No motivational interviewing
Sanders, J., Fitzpatrick, J. M., 2017. Improving service user self-management: development and implementation of a strategy for the Richmond Response and Rehabilitation Team. BMJ Open Qualit. 6(2), e000126. https://doi.org/10.1136/bmjoq-2017-000126	No motivational interviewing
Schrauben, S. J., Inamdar, A., Yule, C., Kwiecien, S., Krekel, C., Collins, C., Anderson, C., Bailey-Davis, L., Chang, A. R., 2022. Effects of dietary app-supported tele-counseling on sodium intake, diet quality, and blood pressure in patients with diabetes and kidney disease. J. Ren. Nutr. 32(1), 39–50. https://doi.org/10.1053/j.jrn.2021.08.006	Age under 65
Scott, S. E., Breckon, J. D., Copeland, R. J., 2019. An integrated motivational interviewing and cognitive-behavioural intervention promoting physical activity maintenance for adults with chronic health conditions: a feasibility study. Chronic Illn. 15(4), 276–292. https://doi.org/10.1177/1742395318769370	Age under 65
Sims, J., Smith, F., Duffy, A., Hilton, S., 1998. Can practice nurses increase physical activity in the over 65s? Methodological considerations from a pilot study. Br. J. Gen. Pract. 48(430), 1249–1250.	No MCCs

MMCs: multiple chronic conditions
