# Supplementary material for: Use of Motivational Interviewing in Older Patients with Multiple Chronic Conditions and Their Informal Caregivers: A Scoping Review
Source: Healthcare (Basel). 2023 Jun 7;11(12):1681. doi: 10.3390/healthcare11121681 (PMC10297925; doi:10.3390/healthcare11121681)
Supplement: Supplementary file 1 [file healthcare-11-01681-s001.zip › Supplementary File 4_R1.pdf]

Supplementary File 4. Characteristics of motivational interviewing interventions in the identified studies (first author alphabetical order)

Article	Type intervention	Provider	Training	Training evaluation	Delivery mode	Location	Session number	Intervention duration	Exposure time per session	Fidelity treatment assessment
Abughosh et al., 2017	MI	Pharmacist students	3-day course by MINT trainer Methodology: lecture, discussion, demonstration, videos, role-playing, simulated phone conversation with trained standardized patients	Evaluation of recorded simulation calls by trainer using a non-standardized instrument	Phone Individual	Home	6	6 months	5-7 min.	ns
Brandberg et al., 2021	MI	Social workers	5-week course by MINT trainer§		Face-to-face or phone Individual	Home, clinic	4-5	4 weeks	10-90 min.	ns
Gonzalez-Bueno et al., 2022	Multi-component	Hospital pharmacists	ns	ns	ns	ns	ns	ns	ns	ns
Halloway et al., 2020	Multi-component	Professional counselor specialized in MI	ns	ns	Phone Individual	Home	9	24 weeks	15 min.	ns
Moral et al., 2015 & Perula-de Torres et al., 2014	MI	Nurses and physicians	20 hrs. workshop by experts in communication skills. Methodology: trigger videos, discussions, role-playing, feed-back and rehearsal	Evaluation of recorded simulated encounters using CICAA and EVEM scales	Face-to-face Individual	Home, healthcare office	3	6 months	Home: 45-60 min. Office: 15 min.	ns
Kim et al., 2020	Multi-component	Primary care providers	ns	ns	Face-to-face Individual	Clinic	ns	ns	ns	ns

Article	Type intervention	Provider	Training	Training evaluation	Delivery mode	Location	Session number	Intervention duration	Exposure time per session	Fidelity treatment assessment
Okuyan et al., 2021	Multi-component	Community pharmacists	4 hrs. virtual training Methodology: Lectures, interactive case presentation, role playing video		Phone and/or face-to-face Individual	ns	ns	3 months	ns	ns
Reed et al., 2018	Multi-component	Nurses and psychologists	ns	ns	Face-to-face, phone Individual	Home	3 (home visits), 4 (calls)	6 months	ns	ns
Steffen et al., 2021	Multi-component	Nurses	20 hrs. workshop by a psychologist expert in MI Methodology: didactic presentations and experiential exercises (role playing)	Using standardized instrument (Importance and confidence ruler to use MI, the conversational interview exercise, helpful responses questionnaire, MISC)	Face-to-face Individual	Clinic	2	2 months	30-50 min.	Post-training using validated instruments (MISC) No during or post intervention
Tiozzo et al., 2019	Multi-component	Care manager nurses	ns	ns	Face-to-face, phone Individual	Home, clinic	ns	ns	ns	ns
Williams et al., 2012a & Williams and Manias, 2014	Multi-component	Nurses	ns	ns	Phone	Home	4	3 months	2-29 min. (mean: 11.75 min.)	ns
Williams et al., 2012b & Williams et al., 2015	Multi-component	Nurses	ns	ns	Phone	Home	4	3 months	3-23 min. (mean: 9.5 min.)	ns

Legend: CICAA: connect (conectar), identify and understand the problems (identificar, comprender), agree (acordar) and help (ayudar); EVEM: Escala para la Valoración de la Entrevista Motivacional-Measuring Motivational Interviewing Skills Scale (MIAS) in English; MI: motivational interviewing; MINT: Motivational interviewing network of trainers; MISC: motivational interviewing skill code; ns: not specified § information derived from the published study protocol (Flink, M., Lindblad, M., Frykholm, O., Kneck, Å., Nilsen, P., Årestedt, K., Ekstedt, M., 2017. The Supporting Patient Activation in Transition to Home (sPATH) intervention: a study protocol of a randomised controlled trial using motivational interviewing to decrease re-hospitalisation for patients with COPD or heart failure. BMJ open, 7(7), e014178. https://doi.org/10.1136/bmjopen-2016-014178)
